# Fragmented imaginary-time evolution for early-stage quantum signal processors

**DOI:** 10.1038/s41598-023-45540-2

**Published:** 2023-10-25

**Authors:** Thais L. Silva, Márcio M. Taddei, Stefano Carrazza, Leandro Aolita

**Affiliations:** 1 Quantum Research Centre, Technology Innovation Institute, Abu Dhabi, UAE; 2 Federal University of Rio de Janeiro, Caixa Postal 68528, Rio de Janeiro, RJ 21941-972 Brazil; 3 ICFO - Institut de Ciencies Fotòniques, The Barcelona Institute of Science and Technology, 08860 Castelldefels, Barcelona Spain; 4 TIF Lab, Dipartimento di Fisica, Università degli Studi di Milano and INFN Sezione di Milano, Milan, Italy

**Keywords:** Information theory and computation, Qubits

## Abstract

Simulating quantum imaginary-time evolution (QITE) is a significant promise of quantum computation. However, the known algorithms are either probabilistic (repeat until success) with unpractically small success probabilities or coherent (quantum amplitude amplification) with circuit depths and ancillary-qubit numbers unrealistically large in the mid-term. Our main contribution is a new generation of deterministic, high-precision QITE algorithms that are significantly more amenable experimentally. A surprisingly simple idea is behind them: partitioning the evolution into a sequence of fragments that are run probabilistically. It causes a considerable reduction in wasted circuit depth every time a run fails. Remarkably, the resulting overall runtime is asymptotically better than in coherent approaches, and the hardware requirements are even milder than in probabilistic ones. Our findings are especially relevant for the early fault-tolerance stages of quantum hardware.

## Introduction

Given a Hamiltonian *H* and an inverse temperature $\beta \ge 0$, QITE is the task of evolving quantum states according to the non-unitary propagator $e^{-\beta H}$. QITE is central not only to ground-state optimisations^1–5^ but also to partition-function estimation and quantum Gibbs-state sampling^6–18^, i.e. the task of preparing thermal quantum states at tunable inverse temperature $\beta$. This is both fundamentally relevant and useful for notable algorithmic applications. For instance, even though approximating ground states of generic Hamiltonians is not expected to be efficient even on a quantum computer—as it can solve QMA-complete problems^19^—, significant speed-ups over classical simulations are possible. This has motivated several ground-state cooling algorithms (with and without QITE), especially for combinatorial optimisations^2,20–23^ or molecular electronic structures^1,24–26^. On the other hand, Gibbs-state samplers are used as main sub-routines for quantum semi-definite program solvers^12–14^ or for training^27–29^ quantum machine-learning models^30,31^, e.g. Moreover, QITE also enables quantizations^2^ of the METTS or Lanczos algorithms, which directly simulate certain thermal properties without Gibbs-state sampling.

Quantum Gibbs states can be approximated by quantum Metropolis Markov-chains^8,9^ or by variational circuits trained to minimise the free energy^16^, e.g. However, the former involve deep and complex circuits, whereas the latter are highly limited by the variational Ansatz. In turn, heuristic QITE algorithms for ground-state optimisations exist^1–5,32–34^. There, one simulates pure-state QITE with a unitary circuit that depends on the input state, the Hamitonian, and $\beta$. For small-$\beta$ steps, one can determine the circuits by measurements on the input state at each step and classical post-processing. One possibility is to optimise a variational circuit on the measured data^1^, but this is again limited by the expressivity of the Ansatz. Another possibility is to invert a linear system generated from the measurements^2–5^, but the size of such system (as well as the number of measurements required) is exponential in the number of qubits, unless restrictive locality assumptions are made.

**Figure 1:**
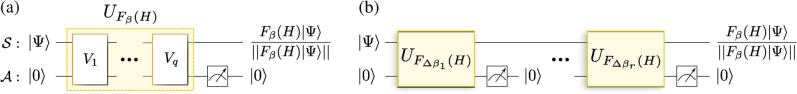
High-level schematics of our algorithms. (**a**) QITE primitives: A system register ${\mathcal {S}}$ carries the input state $|\Psi \rangle$, whereas an ancillary register ${\mathcal {A}}$ is initialised in a computational-basis state $|0\rangle$. A unitary transformation $U_{F_{\beta }(H)}$, composed of a sequence $\{V_k\}_{\in [q]}$ of *q* gates, with $[q]=\{1, \ldots q\}$, is applied and then the ancillas are measured. Each gate makes one query to the Hamiltonian oracle (not shown). The specific choice of gates in the sequence is such that, conditioned on detecting $|0\rangle$ on the ancillas, the desired state $\frac{F_{\beta }(H)|\Psi \rangle }{\left\| F_{\beta }(H)|\Psi \rangle \right\| }$ is output up to controllable error. We refer to the circuit generating $U_{F_{\beta }(H)}$ as a QITE primitive. (**b**) Master QITE algorithms: The post-selection probability—given approximately by $p_\Psi (\beta )=\left\| F_{\beta }(H)|\Psi \rangle \right\| ^{2}$—can decrease with $\beta$ very fast. Hence, for high $\beta$, probabilistic approaches based on repeat-until-success fail for the vast majority of trials. In turn, coherent approaches based on quantum amplitude amplification provide a close-to-quadratic runtime speed-up, but at the expense of enormous circuit depths. In contrast, we introduce a master algorithm that concatenates *r* QITE fragments of inverse temperatures $\{\Delta \beta _l\}_{l\in [r]}$, with $\sum _{l\in [r]}\Delta \beta _l=\beta$ and $\beta _l<\beta$ for all $l\in [r]$. Each fragment is successively run probabilistically and has both a success probability significantly higher and a query complexity significantly lower than that of the entire evolution run at once. This ends up yielding an enormous saving in overall runtime (even beating coherent approaches for high $\beta$) while at the same time preserving all the practical advantages of probabilistic approaches for experimental implementations.

The most general, guaranteed-precision QITE algorithms are based on unitary circuits followed by ancillary-qubit post-selection^6,11,14,15,18^. These circuits—to which we refer as *QITE primitives*—are efficient in $\beta$ as well as in the target precision. However, due to the intrinsically probabilistic post-selection, they must be applied multiple times – by what we refer to as *master QITE algorithms*—to obtain a deterministic output. Repeat-until-success master algorithms apply the primitive in parallel (i.e. in independent probabilistic runs), thereby not inducing any increase in circuit depth. However, their overall complexity is inversely proportional to the post-selection probability. Instead, coherent master algorithms^6,11,14,15^, based on amplitude amplification^35^, have close-to-quadratically smaller overall complexity. However, they require enormous circuit depths and significantly more ancillas. In addition, no fundamental efficiency limit for generic QITE algorithms is known.

### Overview

Here, we introduce two efficient QITE primitives based on the quantum signal processing (QSP) framework^14,15,36,37^ as well as a practical master QITE algorithm (see Fig. 1); and prove a universal lower bound for the complexity of QITE primitives that can be seen as an imaginary-time counterpart of the no fast-forwarding theorem for RTE^38–40^. The first primitive is designed for Hamiltonians given in the well-known block-encoding oracle model, whereas the second one for a simplified model of real-time evolution oracles involving a single time. Both primitives feature excellent query complexity (number of oracle calls) and ancillary-qubit overhead. In fact, for the first primitive the complexity is sub-additive in $\beta$ and $\log (\varepsilon ^{-1})$, with $\varepsilon$ the tolerated error. This scaling saturates our universal bound when $\beta \ll \log (\varepsilon ^{-1})$. Hence Primitive 1 is optimal in that regime, which, interestingly, turns out crucial for our master algorithm. In contrast, Primitive 2’s complexity is multiplicative in $\beta$ and $\log (\varepsilon ^{-1})$, but it requires a single ancilla throughout and its oracle significantly fewer gates. This is appealing for intermediate-scale quantum hardware. In turn, our master QITE algorithm breaks the evolution into small-$\beta$ fragments and runs each fragment’s primitive probabilistically. Surprisingly, this yields an overall runtime competitive with – and, in the relevant regime of high $\beta$, even better than – that of coherent approaches while, at the same time, preserving all the advantages of probabilistic ones for experimental feasibility.

Finally, the complexity of our master algorithm depends on the *fragmentation schedule*, i.e. number *r* of fragments and their relative sizes. On one hand, for Primitive 1, we rigorously prove that, from a critical inverse temperature $\beta _{\text{c}}= {\mathcal {O}}(2^{N/2}\, N)$ on, the runtime is lower than that with coherent QITE. This is shown by explicitly constructing schedules with only $r=2$ fragments that do the job, remarkably. On the other hand, that fragmented QITE outperforms coherent QITE is also observed for both primitives through extensive numerical evidence. More precisely, we study the overall runtime as a function of $\beta$ and $\varepsilon$, up to $N=15$ qubits, and for numerically-optimized schedules. These experiments involve random instances of Hamiltonians encoding four computationally hard classes of problems: Ising models associated to the *i*) MaxCut and *ii*) weighted MaxCut problems^20–22^; *iii*) restricted quantum Boltzmann machines (transverse-field Ising models)^30,31^; and *iv*) a quantum generalization (fully-connected Heisenberg models) of the Sherrington-Kirkpatrick model^41,42^ for spin glasses. We see a clear trend whereby, from $\beta _{\text{c}}= {\mathcal {O}}\big (2^{N/2}\big )$ on, fragmentation outperforms coherent QITE for both primitives, for an optimal number of fragments $r\lesssim 6$. The obtained values for $\beta _{\text{c}}$ imply that our algorithm outperforms coherent QITE in the computationally hardest range of $\beta$, particularly relevant for Hamiltonians with an exponentially small spectral gap^43,44^. Moreover, impressively, such advantages are attained at no cost in circuit depth or number of ancillas, which are identical to those of probabilistic QITE. It is worth noting that, although we prove that fragmented QITE can outperform the coherent algorithm, it does not mean that its scaling is better (see the Supplementary Material^45^, Sec. VII).

## Results

We consider an *N*-qubit system ${\mathcal {S}}$, of Hilbert space ${\mathbb {H}}_{\mathcal {S}}$. We denote by ${\mathbb {H}}_{\mathcal {A}}$ the Hilbert space of an ancillary register ${\mathcal {A}}$. We first discuss the primitives, then the universal complexity lower bound, and the master algorithm at last. Formal definitions and proofs of theorems are found in Methods.

### Quantum imaginary-time evolution primitives

We use the notation $(\beta ,\varepsilon ',\alpha )$-QITE-primitive to refer to a circuit that implements a block-encoding of the QITE propagator, i.e. a unitary $U_{F_\beta (H)}$ acting on ${\mathcal {S}}$ and ${\mathcal {A}}$ containing an $\varepsilon '$-approximation of $\alpha F_\beta (H)$ as one of its matrix blocks, with $0\le \alpha \le 1$ a subnormalization factor, $F_\beta (H):=e^{-\beta (H-\lambda _{\text {min}})}$, and $\lambda _{\text {min}}$ the minimal eigenvalue of *H*. When applied to a state $|\Psi \rangle _{\mathcal {S}} |0\rangle _{\mathcal {A}}$, the primitive (approximatelly) produces the target state $\frac{F_{\beta }(H)|\Psi \rangle }{\left\| F_{\beta }(H)|\Psi \rangle \right\| }$ on the system after postselecting the ancillas in $|0\rangle _{\mathcal {A}}$. The postselection success probability is given by $p_{\Psi }(\beta , \alpha )=\alpha ^2\, \left\| F_{\beta }(H)|\Psi \rangle \right\| ^2$. The trace-distance error in the output-state is ${\mathcal {O}}(\varepsilon )$ if the spectral error in the primitive is $\varepsilon ^{\prime }\le \,\varepsilon \, \sqrt{p_{\Psi }(\beta , \alpha )}/2$^45^, Sec. II.

We introduce two QITE primitives. Both of them possess the basic structure shown in Fig. 1a, where a sequence of gates $\{V_k\}_{\in [q]}$, with $[q]=\{1, 2, \ldots q\}$, generates an approximate block-encoding of $F_\beta (H)$. The circuit acts on the system, block-encoding ancillas and at most one extra qubit ancilla. The approximation consists of truncating an expansion of the exponential function at finite order *q*. Each gate $V_k$ makes one call to the oracle of *H* (or its inverse) and contains ${\mathcal {O}}(1)$ parameterized single qubit rotations. The parameters of this gates are determined by the function expansion using quantum signal processing^14,15,36,37^. Conceptually, the two primitives differ in the kind of expansion and the type of oracle. Their circuit descriptions are given in the Methods, especially in Fig. 6.

The first primitive implements a Chebyshev expansion using a block-encoding oracle $O_1$, i.e a unitary that has *H* as one of its blocks. We denoted by $|{\mathcal {A}}_{O_1}|$ the ancillary-register size and by $g_{O_1}$ the gate complexity of $O_1$. In Methods, we prove the following.

#### Theorem 1

(QITE primitive using Chebyshev approximation and block-encoding oracles). Given $0<\varepsilon '<1$ and $\beta >0$, there is a circuit $P_{1}$ that is a $(\beta ,\varepsilon ',1)$-QITE-primitive using

1 $$\begin{aligned} q_{{1}}(\beta ,\varepsilon ') = {\mathcal {O}}\left( \frac{e\,\beta }{2}+\frac{\ln (1/\varepsilon ')}{\ln \left[ e+2\ln (1/\varepsilon ')/(e\,\beta )\right] }\right) \end{aligned}$$

queries to $O_1$ and $O_1^\dagger$, $|{\mathcal {A}}_{1}|=|{\mathcal {A}}_{O_1}|+1$ total ancillary qubits, and gate complexity $g_{P_1}={\mathcal {O}}(g_{O_1}+|{\mathcal {A}}_{O_1}|)$ per query. Moreover, the classical run-time to calculate the gates of $P_1$ is ${\mathcal {O}}(\text{poly}\big (q_1(\beta ,\varepsilon ')\big )$.

A nice feature of Eq. (1) is its sub-additivity in $\beta$ and $\ln (1/\varepsilon ')$. We note that a QITE primitive was obtained in^15^ that works for the same oracle model and has complexity upper-bounded by ${\mathcal {O}}\big (\sqrt{2\,\max [e^2\,\beta , \ln (2/\varepsilon ')]\,\ln (4/\varepsilon ')}\big )$. This is asymptotically better in $\beta$ than Eq. (1), but it underperforms it for all $\beta \lesssim 8\ln (4/\varepsilon ')$. In particular, while Eq. (1) tends to zero for $\beta \rightarrow 0$, the bound from Ref.^15^ tends to ${\mathcal {O}}\big (\ln (1/\varepsilon ')\big )$. Interestingly, the strict upper bound that we obtain in Methods is the expression within ${\mathcal {O}}( )$ in Eq. (1) up to a modest factor: 8. Moreover, in^45^, Sec. V, we numerically verify that that expression is itself a valid bound (no extra factor), even for low $\beta$. Most importantly, in section "Cooling-speed limits for oracle-based QITE algorithms" we show that it approaches the optimal scaling as $\beta$ decreases relative to $\ln (1/\varepsilon ')$. We stress that the latter regime is crucial for the master algorithm of section "Fragmented master QITE algorithm", whose first fragments require, precisely, low inverse temperatures and high precisions. In turn, in the opposite regime of high $\beta$, preliminary numerical observations^46^ suggest that the asymptotic scaling of the exact value of $q_1$ could actually be as good as $q_1(\beta ,\varepsilon ')={\mathcal {O}}\big (\sqrt{\beta \,\ln (1/\varepsilon ')}\big )$, i.e. similar to that from^15^.

The second primitive implements a Fourier expansion assuming access to a unitary oracle $O_2$, with gate complexity $g_{O_2}$, that contains the time evolution $e^{-iHt}$ at time $t=\frac{\pi }{2}\big (1+\frac{\gamma }{\beta }\big )^{-1}$. In Methods, we prove the following.

#### Theorem 2

(QITE primitive using Fourier approximation nd single real-time evolution oracles). Given $0<\varepsilon '<1$ and $\beta >0$, there is a $(\beta ,\varepsilon ',\alpha )$-QITE-primitive $P_{2}$ with $\alpha =e^{-\beta (1+\lambda _{\text{min}})-\gamma }$, it uses

2 $$\begin{aligned} q_{{2}}(\beta ,\varepsilon ',\alpha ) = {\mathcal {O}}\big ((\beta /\gamma +1)\ln (4/\varepsilon ')\big ), \end{aligned}$$

queries to $O_2$ and $O_2^\dagger$, $|{\mathcal {A}}_{2}|=1$ ancilla, and $g_{P_2}=g_{O_2}+{\mathcal {O}}(1)$ gates per query. Moreover, the gates of $P_2$ are obtained in classical runtime ${\mathcal {O}}(\text{poly}\big (q_2(\beta ,\varepsilon ',\alpha )\big )$.

As shown in Methods, the “${\mathcal {O}}( \cdot )$” in Eq. (2) also hides only a modest global factor: 4. In contrast to Eq. (1), the relation between $\beta$ and $\ln (1/\varepsilon ')$ in Eq. (2) is multiplicative. However, in return, $P_2$ requires $|{\mathcal {A}}_2|=1$ ancillary qubit throughout, remarkably. This is a drastic reduction relative to block-encoded oracle algorithms, and also to other algorithms based on real-time evolution. The latter is due to the use of a single real-time instead of an error-dependent number of them^6,14^. In fact, $|{\mathcal {A}}_2|=1$ is the minimum possible, because, since $F_\beta (H)$ is non-unitary, at least 1 ancilla is needed to block-encode it. Moreover, the scaling of $g_{P_2}$ is optimal too. Since it is based on real-time evolution oracles, it requires no qubitization^37^. Consequently, it adds only a small, constant number of gates per query to the intrinsic gate complexity $g_{O_2}$ of the oracle. These features make $P_2$ specially well-suited for near-term devices. Importantly, rather than a peculiarity of $P_2$, the favourable scalings of $|{\mathcal {A}}_{2}|$ and $g_{P_2}$ are generic features of the type of operator-function design behind it: An optimised Fourier-approximation algorithm for arbitrary analytical real functions of Hermitian operators^47^.

Our algorithms support any $\lambda _{\text{min}}\in [-1,1]$. For $P_2$, this is reflected by the sub-normalization factor $e^{-\beta (1+\lambda _{\text{min}})}$, which decreases as $\lambda _{\text{min}}$ departs from $-1$. In turn, the other factor, $e^{-\gamma }$, arises from the Gibbs phenomenon of Fourier series. The theorem holds for all $\gamma \ge 0$, allowing one to trade success probability for query complexity. For $\varepsilon ^{\prime }\ll 1$, the optimal value of $\gamma$ depends only on $\beta$ for both coherent and probabilistic algorithms^45^, Sec. III].

Finally, Theorems 1 and 2 can be straightforwardly extended to the realistic case of approximate oracles: In^45^, Sec. I, we show (for generic analytical operator functions) that it suffices to take the oracle error (deviation from an ideal oracle) as $\varepsilon '_O=O(\varepsilon '/q)$ to keep the primitive’s error in $O(\varepsilon ')$.

### Cooling-speed limits for oracle-based QITE algorithms

The most challenging applications of QITE involve small post-selection probabilities, decreasing exponentially in *N* in the worst cases. In an effort to reduce the overall complexity [see Eq. (4)], this has fueled a long race^6,7,11,12,14,15^ to improve $q(\beta ,\varepsilon ')$, going from the seminal ${\mathcal {O}}\big (\beta \,\text{poly}(1/\varepsilon ')\big )$ of^6^ to the recent ${\mathcal {O}}\big (\sqrt{2\max [e^2\,\beta , \ln (2/\varepsilon ')]\,\ln (4/\varepsilon ')}\big )$ of^15^ or the additive scaling of Eq. (1). However, to our knowledge, no runtime limit for QITE simulations has been established. This contrasts with real-time evolution (RTE), where fundamental runtime lower bounds are given by the “no-fast-forwarding theorem”^38–40^. These are saturated by optimal RTE algorithms^15,36,37^. Here we derive an analogous bound for imaginary time, which we call cooling-speed limit in allusion to the use of QITE to cool systems down to their ground state.

More precisely, we prove a universal efficiency limit for QITE primitives based on block-encoded oracles. This is convenient as it directly applies to our primitive with lowest query complexity, i.e. $P_1$.

#### Theorem 3

(Imaginary-time no-fast-forwarding theorem) Let $\beta >0$ and $0<\varepsilon '<\alpha /2$. Then, any $(\beta ,\varepsilon ',\alpha )$-QITE-primitive querying block-encoding Hamiltonian oracles has query complexity at least $q_{\min }(\beta ,\varepsilon ',\alpha )\ge {{\tilde{q}}}$, where ${{\tilde{q}}}\in {\mathbb {R}}_{>0}$ is the unique solution to the equation

3 $$\begin{aligned} \left| \frac{1-e^{-\frac{\beta }{4{{\tilde{q}}}}}}{2}\right| ^{2\,{{\tilde{q}}}} = \frac{2\,\varepsilon '}{\alpha }. \end{aligned}$$

Even though the bound is only given implicitly, interesting conclusions can readily be drawn. First, for any fixed $\beta$, the left-hand-side of Eq. (3) decreases monotonically with ${{\tilde{q}}}$ (therefore the uniqueness of the solution). Second, for any fixed $\varepsilon '$ and $\alpha$, ${{\tilde{q}}}$ grows monotonically with $\beta$. Third, and most important, Eq. (3) is approximated by $\big (\frac{\beta }{{8}\,{{\tilde{q}}}}\big )^{2{{\tilde{q}}}}=2\,\varepsilon '/\alpha$ for $\beta \ll {{\tilde{q}}}$, as a Taylor expansion shows. The latter equation has a known explicit solution^15^, which, for $\alpha =1$, is given by Eq. (1). Hence, for $\beta /{{\tilde{q}}}\rightarrow 0$, Eq. (1) tends to the optimal scaling. Note that $\beta \ll {{\tilde{q}}}$ is equivalent to the first term Eq. (1) being much smaller than its second term, which in turn implies that $\varepsilon '$ should be exponentially small in $\beta$. Thus, $P_1$ is close to optimal for small inverse temperatures or high precisions. Interestingly, this is the regime at which the first fragments of our master algorithm operate, as we see next.

### Fragmented master QITE algorithm

We call master QITE algorithm a procedure which incorporates the primitives to attain deterministic QITE. It means that these algorithms deterministically produce the state $\frac{F_{\beta }(H)|\Psi \rangle }{\left\| F_{\beta }(H)|\Psi \rangle \right\| }$, up to trace-distance error $\varepsilon$, if they are given an input state $|\Psi \rangle \in {\mathbb {H}}_{\mathcal {S}}$.

Until now, two variants of master QITE algorithms had been reported, probabilistic and coherent (see Fig. 7). The former leverage repeat-until-success: apply $P_{\beta ,\varepsilon ^{\prime },\alpha }$ (on independent systems) until getting the desired output. Every time the postselection on the ancillas is not successful the resulting system state is discarded and system and ancillas are reinitialized for a new trial. The average number of trials until one gets one success is given as ${\mathcal {O}}(1/p_{\Psi }(\beta , \alpha ))$. In contrast, the latter are based on quantum amplitude amplification^35^. There, $P_{\beta ,\varepsilon ^{\prime },\alpha }$ is incorporated into a unitary amplification engine that is sequentially applied (on the same system) ${\mathcal {O}}\big (\sqrt{1/p_{\Psi }(\beta , \alpha )}\big )$ times. Hence, the overall query complexity of both variants is given by the unified expression

4 $$\begin{aligned} Q_\kappa (\beta ,\varepsilon , \alpha )={\mathcal {O}}\bigg (\frac{1}{{\big (p_{\Psi }(\beta , \alpha )}\big )^{ {\mu _{\kappa }}}}\,q(\beta ,\varepsilon ^{\prime }, \alpha )\bigg ), \end{aligned}$$

where $\kappa =\,$prob/coh for probabilistic or coherent schemes, respectively, $\mu _{\text{prob}}=1$, $\mu _{\text{coh}}=1/2$, and $\varepsilon ^{\prime }=\varepsilon \sqrt{p_{\Psi }(\beta , \alpha )}/2$. Since $p_{\Psi }(\beta , \alpha )$ can decrease with *N* exponentially, the quadratic advantage in $1/p_{\Psi }(\beta , \alpha )$ of coherent approaches is highly significant. However, coherent algorithms have a circuit depth ${\mathcal {O}}(\sqrt{1/p_{\Psi }(\beta , \alpha )})$ times greater than in probabilistic ones and require ${\mathcal {O}}(N)$ extra ancillas. This makes coherent schemes impractical for intermediate-scale quantum devices.

Our master algorithm relies on the basic identity $F_\beta (H)=\prod _{l=1}^{r} F_{\Delta \beta _l}(H)$ to partition the evolution into $r\in {\mathbb {N}}$ fragments of inverse temperatures $S_r=\{\Delta \beta _l>0\}_{l\in [r]}$, such that $\sum _{l\in [r]}\Delta \beta _l=\beta$. We refer to $S_r$ as the *fragmentation schedule*. For each *l*, the algorithm repeats until success a $(\Delta \beta _l,\varepsilon ^{\prime }_l,\alpha _l)$-QITE-primitive $P_{\Delta \beta _l,\varepsilon ^{\prime }_l,\alpha _l}$ on the output state $|\Psi _{l-1}\rangle$ of the $(l-1)$-th step, with $\varepsilon ^{\prime }_l$ given in Eq. (5). That is, if the ancillas ${\mathcal {A}}$ are successfully post-selected in state $|0\rangle$, the system’s output state $|\Psi _{l}\rangle$ is input into the $(l+1)$-th fragment. Else, the algorithm starts all over from the first fragment on $|\Psi _{0}\rangle =|\Psi \rangle$, until $|\Psi _{l-1}\rangle$ is prepared and the *l*-th fragment can be run again. Alternatively, the measurement on ${\mathcal {A}}$ after each fragment can be seen as monitoring that the correct block of $U_{F_{\Delta \beta _l}(H)}$ is applied on each $|\Psi _{l-1}\rangle$, in contrast to the single error detection after $U_{F_{\beta }(H)}$ in the probabilistic master algorithm (see Fig. 1). Note that the total number of trials (i.e. preparations of $|\Psi \rangle$) coincides with the number of repetitions of the first fragment. We also note that our method resembles the discrete formulation of the Zeno effect applied in the quantization of the Metropolis-Hastings walk for classical Hamiltonians^48^. However, here we cannot apply the rewind technique, i.e iterate between two consecutive steps of inverse temperature instead of rebooting in case of a failure in the postselection^49^. Rewind applied to fragmented QITE would not produce the right output state. The following pseudocode summarizes all the algorithm:

**Algorithm 1:**
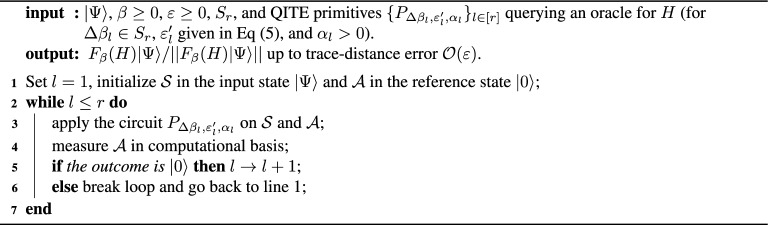
Fragmented QITE.

The correctness and complexity of Algorithm 1 are established by the following theorem, proven in the Suplementary Material^45^, Sec. IV.

#### Theorem 4

(Fragmented master QITE algorithm). If

5 $$\begin{aligned} \varepsilon '_l\le \left\{ \begin{array}{lr} \frac{\varepsilon \,\prod _{k=1}^r\alpha _k}{2\times 4^{r-1}}\sqrt{p_{\Psi }(\beta )} &{} \text { if } l=1,\\ \frac{\varepsilon \,\prod _{k=l}^r\alpha _k}{4^{r-l+1}}\sqrt{\frac{p_{\Psi }(\beta )}{p_{\Psi }(\beta _{l-1})}} &{} \text { if } l>1,\\ \end{array} \right. \end{aligned}$$

for all $l\in [r]$, Algorithm 1 is a master QITE algorithm for *H* on $|\Psi \rangle$ with error ${\mathcal {O}}(\varepsilon )$ and average query complexity

6 $$\begin{aligned} {Q_{S_r}(\beta ,\varepsilon ) =\sum _{l=1}^{r} n_l\, q(\Delta \beta _l,\varepsilon ^{\prime }_l, \alpha _l)}, \end{aligned}$$

where $n_l=\frac{p_{\Psi }(\beta _{l-1})}{p_{\Psi }(\beta )\prod _{k=l}^r\alpha ^2_k}$ is the average number of times that $P_{\Delta \beta _l,\varepsilon ^{\prime }_l,\alpha _l}$ is run, with $\beta _0=0$, $\beta _l=\sum _{k=1}^{l}\Delta \beta _k$ for all $l\in [r]$, and $p_{\Psi }({\tilde{\beta }})=\Vert F_{{\tilde{\beta }}}(H)|\Psi \rangle \Vert ^2$ for any ${\tilde{\beta }}$.

**Figure 2:**
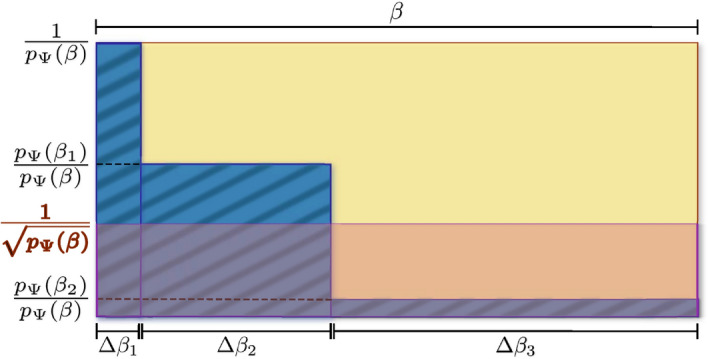
Intuition behind the complexity reduction by fragmentation. The overall complexity of the probabilistic master algorithm is dominated by the area of the yellow rectangle. In contrast, the corresponding complexity of the fragmented algorithm (here, for the exemplary case of $r=3$ fragments) is dominated by the area of the blue-shaded rectangles. Up to logarithmic corrections in the precision, the cumulative width of the blue-shaded rectangles coincides with the width of the yellow one, of order $\beta$. In contrast, while the height of the yellow rectangle is of order $1/p_{\Psi }(\beta )$, the height of the blue-shaded ones decreases from order $1/p_{\Psi }(\beta )$ till order $p_{\Psi }(\beta _{r-1})/p_{\Psi }(\beta )$, making the blue-shaded area smaller than the yellow one. For high-enough $\beta$, the reduction can be so strong that the complexity of the fragmented algorithm can reach even that of the coherent algorithm which is represented by the area of the pink rectangle with height $1/\sqrt{p_{\Psi }(\beta )}$. This intuition is rigorously proven for Primitive 1 (in Theorem 5) and numerically verified to exhaustion for both Primitives 1 and 2 (in section "Fragmented quantum Gibbs-state samplers").

We note that, for Primitive 1, the average total number of trials coincides with that of the probabilistic algorithm: $n_1=1/p_{\Psi }(\beta )=:n_{\text{prob}}$ (see Methods). This is important because the probabilistic algorithm consumes $q_1(\beta ,\varepsilon ^{\prime })$ queries per trial, successful or not. In contrast, the fragmented one consumes per trial $q_1(\Delta \beta _1,\varepsilon ^{\prime }_1)$ queries, plus $q_1(\Delta \beta _2,\varepsilon ^{\prime }_2)$ queries only if the first post-selection succeeds, plus $q_1(\Delta \beta _3,\varepsilon ^{\prime }_3)$ queries only if the second one succeeds too, and so on. Hence, the total waste in queries is lower with fragmentation (see Fig. 2). The strength of the reduction depends on how fast $p_{\Psi }(\beta _l)$ (and so $n_l$) decreases with *l*; but, in any case, it gets more drastic as $\beta$ increases. That is, the largest reductions are expected at the hardest regime of $p_{\Psi }(\beta )\ll 1$. To maximize the effect, one wishes $q_1(\Delta \beta _l,\varepsilon ^{\prime }_l)$ to decrease with *l* as fast as possible. Note that Eq. (5) implies $\varepsilon ^{\prime }_l< \varepsilon ^{\prime }_{l+1}$, which plays against the latter wish. However, fortunately, $q_1(\Delta \beta _l,\varepsilon ^{\prime }_l)$ grows approximately linearly in $\Delta \beta _l$ but sub-logarithmically in $1/\varepsilon ^{\prime }_l$. Hence, for sufficiently high $\beta$, one can make $q_1(\Delta \beta _l,\varepsilon ^{\prime }_l)$ arbitrarily smaller than $q_1(\Delta \beta _{l+1},\varepsilon ^{\prime }_{l+1})$ by choosing $\Delta \beta _l$ sufficiently smaller than $\Delta \beta _{l+1}$.

Based on these heuristics, we next prove for Primitive 1 that Algorithm 1 can not only outperform the probabilistic algorithm but also—for sufficiently high $\beta$—even the coherent one, surprisingly. The proof is constructive: we devise suitable schedules that give the desired advantage for fragmentation. Remarkably, it is enough to consider only $r=2$ fragments. The result is valid for any $|\Psi \rangle$ and *H*, under only mild assumptions on the success probability $p_{\Psi }$ as a function of $\beta$. We denote the inverse function of $p_{\Psi }$ by $p^{-1}_{\Psi }$. For simplicity, we state the theorem explicitly for the restricted case of *H* non-degenerate, with a unique ground state $|\lambda _{\text{min}}\rangle$ of overlap ${o}^2=|\langle \lambda _{\text{min}}|\Psi \rangle |^2$ with $|\Psi \rangle$. However, it can be straightforwardly generalized to the degenerate case by redefining ${o}^2$ as the overlap with the lowest-energy subspace.

#### Theorem 5

(Fragmented QITE outperforms coherent QITE) Let $|\lambda _{\text{min}}\rangle \in {\mathbb {H}}_{\mathcal {S}}$ be the unique ground state of *H* and $|\Psi \rangle \in {\mathbb {H}}_{\mathcal {S}}$ such that $0<{o}\le 1/2.2$. Define the critical inverse temperature $\beta _c=\frac{2}{{o}}\left[ \frac{2}{e}\ln \left( \frac{8}{{o}\,\varepsilon }\right) +p^{-1}_{\Psi }(\frac{{o}}{2.2})\right]$. Then, if (*H* and $|\Psi \rangle$ are such that) $p_{\Psi }(\beta _c){\le 1/4}$, there exists a two-fragment schedule $S_2$ for which, for $P_1$, it holds that $Q_{S_2}(\beta ,\varepsilon )<Q_{\text {coh}}(\beta ,\varepsilon )$ for all $\beta \ge \beta _c$ and $0<\varepsilon <1$. In particular, $S_2=\big \{\Delta \beta _1={p^{-1}_\Psi \big (\frac{{o}}{2}\frac{1}{{\ln [e+2\ln (2/{o}\,\varepsilon )/e\beta ]}}\big )},\Delta \beta _2=\beta -\Delta \beta _1\big \}$ is a valid choice of such schedules.

The proof is given in the Supplementary Information^45^, Sec. VI. The schedules constructed there have the sole purpose of proving the existence of $\beta _c$ in general and are therefore not necessarily optimal for each specific *H* and $|\Psi \rangle$. For instance, in^45^, Sec. VIII, we study Gibbs-state sampling (i.e. for the maximally-mixed state as input, with ${o}=2^{-N/2}$) for *H* describing non-interacting particles, where a closed-form expression for $p_{\Psi }(\beta )$ can be obtained. For this simple case, the theorem yields $\beta _c={\mathcal {O}}\big (2^{N/2}\, N\big )$. However, in section "Fragmented quantum Gibbs-state samplers" we numerically optimize the schedules and obtain $\beta _c={\mathcal {O}}\big (2^{N/2}\big )$ for hard-to-simulate, interacting systems. The proof exploits the additive dependence of $q_{{1}}$ on $\beta$ and the logarithmic term in Eq. (1). Its extension to the multiplicative case of $q_{2}$ is left for future work. Nevertheless, here, we do consistently observe an advantage of fragmented QITE over coherent one for $P_2$. More precisely, in section "Fragmented quantum Gibbs-state samplers", we numerically find that also for $P_2$ does fragmentation outperform coherent-QITE at Gibbs-state sampling, with $\beta _c$ scaling with *N* as in $P_1$ but with a somewhat larger pre-factor (which is expectable, as $\alpha _l<1$ gives an exponential dependance of $n_l$ on *r* that worsens the performance). Either way, that fragmentation can outperform quantum amplitude amplification at all is remarkable, since the latter requires circuits ${\mathcal {O}}(\sqrt{1/p_{\Psi }(\beta )})$ times deeper and ${\mathcal {O}}(N)$ more ancillas than the former.

Our findings would have little practical relevance if $\beta _{\text{c}}$ was unphysically high. Fortunately, $\beta _{\text{c}}={\mathcal {O}}\big (2^{N/2}\big )$ is in an intermediate regime useful for important applications: E.g., Ground-state cooling (or, more generally, Gibbs-state sampling at low temperatures) requires $\beta$ scaling inversely proportionally to the spectral gap, which can be exponentially small in *N* even for relatively simple Hamiltonians such as transverse-field Ising models^43,44^. In fact, in section "Fragmented quantum Gibbs-state samplers" we compare $\beta _{\text{c}}$ with the inverse temperatures $\beta _{0.9}$ needed for a modest ground-state fidelity 0.9. We systematically observe that $\beta _{\text{c}}$ is either greater than or close to $\beta _{0.9}$, evidencing the relevance of the regime of advantage of fragmented over coherent QITE. Finally, as mentioned, $P_1$ is particularly well-suited for fragmentation. On the one hand, it displays $\alpha _l=1$ for all $l\in [r]$. On the other hand, and most importantly, $q_1$ becomes optimal as $\beta$ decreases relative to $\ln (1/\varepsilon ')$. This is convenient to minimize Eq. (6), because the first fragments (specially the first one) operate precisely at low $\Delta \beta _l$ and $\varepsilon ^{\prime }_l$, close to that optimality regime. The latter is verified both analytically for the non-interacting case of^45^, Sec. VIII, and numerically for the examples of section "Fragmented quantum Gibbs-state samplers" in^45^, Sec. IX, where we consistently observe that $\beta _1$ is typically only a tinny fraction of $\ln (1/\varepsilon _1')$. Colloquially speaking, the widths of the first blue-shaded rectangles in Fig. 2 can be reduced more with $P_1$ than with other primitives.

**Figure 3:**
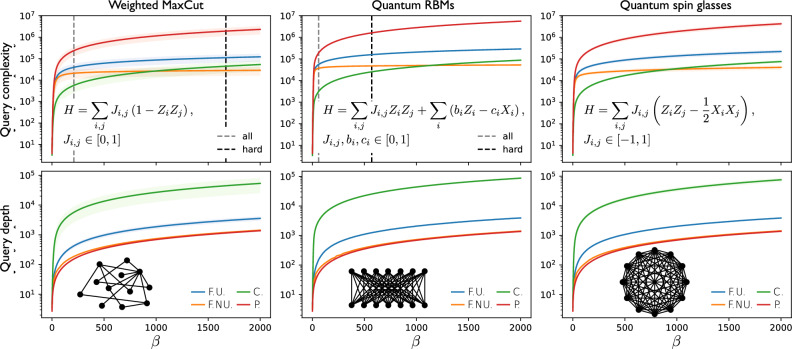
Runtimes and circuit depths of quantum Gibbs-state samplers running on Primitive 1 versus inverse temperature. Red corresponds to the probabilistic master QITE algorithm (P), green to the coherent one (C), blue to the fragmented one with uniform schedule $S_{r}$ for the best *r* (F.U. for fragmented uniform), and orange to the fragmented one with a schedule $S_{r,a}$ as in Eq. (7) for the best *r* and *a* (F.NU. for fragmented non-uniform) (see also Fig. 5). Three classes of Hamiltonians are shown (expressions in upper panels and lattice geometries in lower ones). Solid curves represent the means over 1000 random instances from each class, whereas shaded areas are the corresponding standard deviations. The examples shown correspond to $N=12$ qubits and a tolerated error of $\varepsilon =10^{-3}$, but qualitatively identical behaviors are observed for all *N* between 2 and 15 as well as for $\varepsilon =10^{-2}$ and $\varepsilon =10^{-1}$. Upper panels: average overall query complexity. Both fragmented algorithms comfortably outperform the probabilistic one already at small $\beta$. In addition, fragmentation with non-uniform schedule outperforms even coherent QITE at a critical inverse temperature $\beta _c$. The black and gray vertical dashed lines mark respectively the values $\beta ^{(\text {aver})}_{0.9}$ and $\beta ^{(\text {hard})}_{0.9}$ at which the average fidelity with the ground state (over all instances and over the $10\%$ of them with the smallest gaps) reaches a modest value of 0.9 (not shown in the third panel because they lie beyond the range of $\beta$ shown; see^45^, Sec. X. Both in the first and second panels, $\beta ^{(\text {aver})}_{0.9}$ is smaller than $\beta _\text {c}$, but the complexity of fragmented QITE at $\beta ^{(\text {aver})}_{0.9}$ is already significantly smaller than that of probabilistic QITE. These considerations imply that fragmented QITE is either competitive or directly superior to coherent QITE for ranges of $\beta$ that are highly relevant for ground state preparation, e.g. The advantage of fragmentation becomes more evident when we compare the average query depths in the lower panels. Defined as the maximum number of queries per circuit run (i.e., not taking into account independent trials), the query depth quantifies the circuit depth (relative to the depth per query) required by one successful run.

### Fragmented quantum Gibbs-state samplers

We benchmark the performance of Algorithm 1 at quantum Gibbs-state sampling by comparing Eqs. (6) and (4) for four classes of spin-1/2 systems: Ising models associated to the *i*) MaxCut and *ii*) weighted MaxCut problems^20–22^; *iii*) transverse-field Ising interactions on the restricted-Boltzmann-machine (RBM) geometry^30,31^; and *iv*) Heisenberg all-to-all interactions, corresponding to a quantum generalization of the Sherrington-Kirkpatrick model^41,42^ for spin glasses. All four classes feature long-range frustation; and classically simulating their Gibbs states (for random instances) is a computationally-hard task^50–54^.

The Gibbs state $\varrho _{\beta }=\frac{e^{-\beta (H-\lambda _{\text{min}})}}{Z_{\beta }}$ of *H* at $\beta$, with $Z_{\beta }=\text {Tr}\left[ e^{-\beta (H-\lambda _{\text{min}})}\right]$ its partition function, can be prepared by QITE at $\beta /2$ on the maximally-mixed state $\varrho _0=\frac{\mathbbm {1}}{Z_{0}}$, where $Z_{0}=2^{N}$. Hence, the post-selection probability is $p_{\Psi }(\beta /2, \alpha )=\alpha ^2\, \frac{Z_{\beta }}{Z_{0}}$, where $\alpha =1$ for $P_1$ and $e^{-\beta (1+\lambda _{\text{min}})-\gamma }$ for $P_2$. This, together with Eqs. (1) and (2), determine the overall query complexities, with respect to $P_1$ and $P_2$, respectively, for the three master algorithms: probabilistic [Eq. (4) for $\kappa =$ prob], coherent [Eq. (4) for $\kappa =$ coh], and fragmented [Eq. (6)]. More technically, rather than Eqs. (1) or (2) we use their ceiling functions, to guarantee that each fragment’s query complexity is integer.

For *N* up to 15 qubits, we draw 1000 random *H*’s within each class. For fair comparison, we re-scale all *H*’s so that $\lambda _{\text{min}}=-1$ and $\lambda _{\text{max}}=1$. For each of them, we calculate the complexities for $\beta$ between 0 and 10000 and $\varepsilon =0.1$, 0.01, or 0.001. Partition functions are evaluated by exact diagonalization of *H*. Evaluating Eq. (6) requires in addition a choice of schedule. We propose

7 $$\begin{aligned} S_{r,a}=\left\{ \left[ \left( \frac{l}{r}\right) ^a-\left( \frac{l-1}{r}\right) ^a\right] {\beta /2}\right\} _{l\in [r]}, \end{aligned}$$

for $a>1$, so that $\beta _l=\big (\frac{l}{r}\big )^a{\beta /2}$ for all $l\in [r]$. This guarantees that $\Delta \beta _1<\Delta \beta _{2} \ldots <\Delta \beta _{r}$ and allows us to control the strength of the inequalities by varying *a*. For each problem instance (*N*, *H*, and $\beta$), we sweep *r* and, for each value of *r*, we find the optimal *a* through the Broyden-Fletcher-Goldfarb-Shanno (BFGS) algorithm until minimizing $Q_{S_{r,a}}({\beta /2},\varepsilon )$^55^.

**Figure 4:**
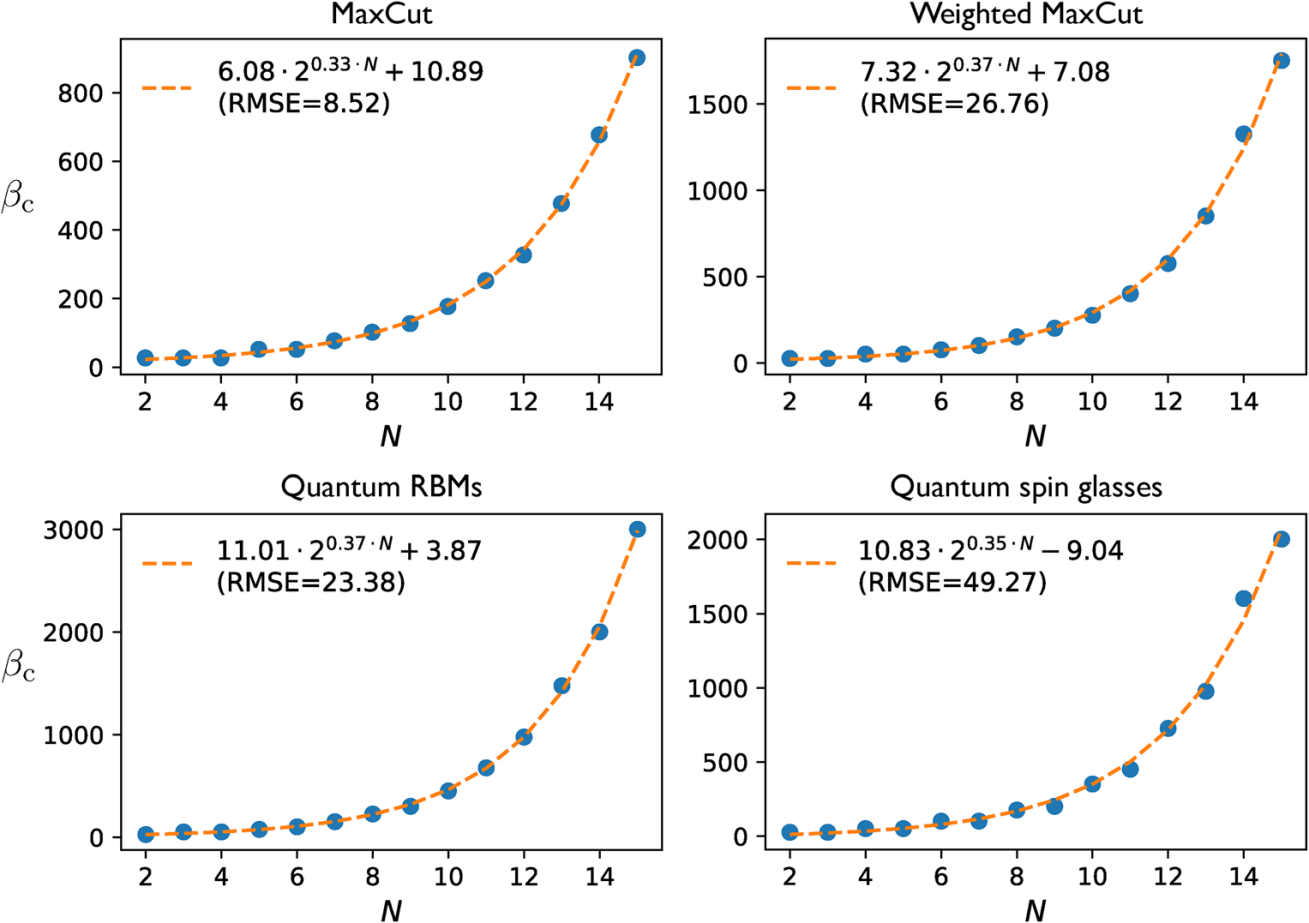
Critical inverse temperatures for $P_1$ versus number of qubits. The error and Hamiltonian classes are the same as in Fig. 3, except for MaxCut, defined as weighted MaxCut but with random $J_{i,j}\in \{0,1\}$ for all (*i*, *j*). Blue dots represent the means over 1000 instances from each class, whereas dashed orange curves their fits over the Ansatz $\beta _{\text{c}}(N)=A\, 2^{\eta \, N}+ B$, with $A, B, \eta \in {\mathbb {R}}$. The fit results, together with their root-mean-square deviations (RMSDs), are shown in the insets. Similar scalings with *N* are observed for $P_2$ (Suplementary Material^45^, Sec. XI). In all cases, $\beta _{\text{c}}={\mathcal {O}}\big (2^{N/2}\big )$ is satisfied.

**Figure 5:**
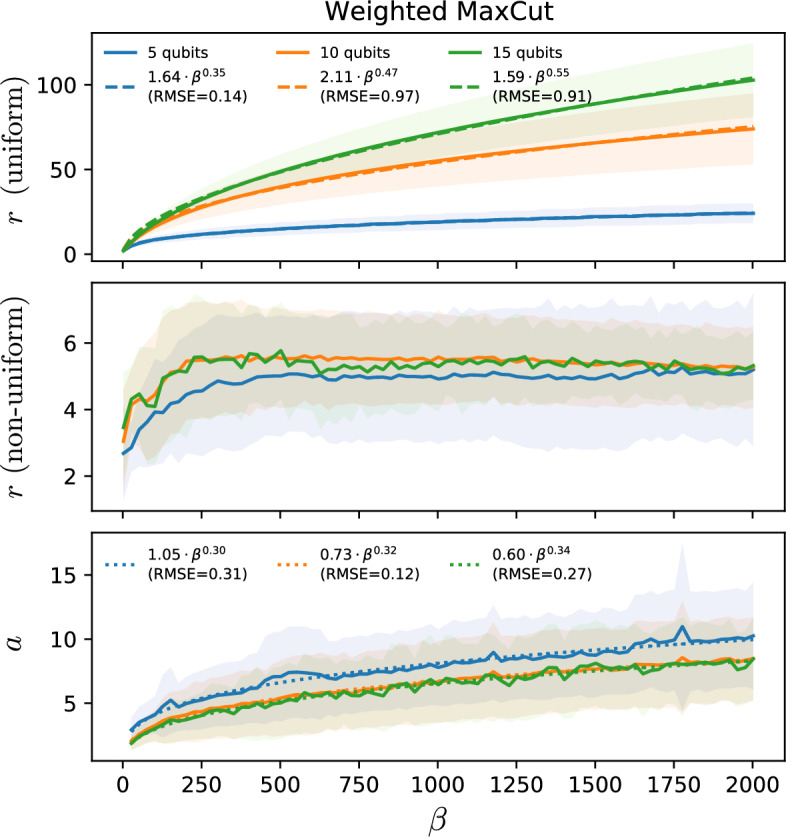
Optimal fragmentation schedules for Primitive 1 versus inverse temperature. System sizes are $N=5$ (blue), $N=10$ (orange), and $N=15$ (green). Solid curves represent the means over 1000 random weighted-MaxCut Hamiltonians, whereas (the thicknesses of) shaded curves are the standard deviations. The tolerated error is $\varepsilon =10^{-3}$. Qualitatively identical behaviors are observed for all *N* between 2 and 15 as well as for $\varepsilon =10^{-2}$ and $\varepsilon =10^{-1}$; and the same holds for the other Hamiltonian classes in Fig. 4. The upper panel shows the optimal number of fragments *r* for uniform schedules $S_{r,1}$. The central and lower panels respectively show the optimal *r* and *a* for non-uniform schedules $S_{r,a}$. The dashed and dotted curves in the upper and lower panels respectively represent fits over the ansätze $r(\beta )=A\, \beta ^{\eta }$ and $a(\beta )=A\, \beta ^{\eta }$, with *A* and $\eta \in {\mathbb {R}}$. The fit results are shown in the insets. Remarkably, for non-uniform schedules, the observed scaling for *r* is constant not only with $\beta$ but also with *N*.

The overall complexities and circuit depths obtained (together with those for uniform schedules, i.e. with fixed $a=1$) are shown in Fig. 3 for $P_1$; and the scalings with *N* of $\beta _{\text{c}}$ in Fig. 4. Similar scalings for the critical inverse temperature are obtained for $P_2$ but with somewhat higher constant pre-factors (see^45^, Sec. XI), which is expectable due to the non-unit sub-normalization factors $\alpha _k$ in $n_l$. Summarizing, our numerical experiments support the following observation.

#### Observation 6

(Gibbs-state sampling with fragmented QITE). Let the primitives be of fixed type, either $P_1$ or $P_2$. Then, for every *H* and $\varepsilon >0$ studied, there exists $\beta _{\text{c}}={\mathcal {O}}\big (2^{N/2}\big )$ such that, for all $\beta \ge \beta _{\text{c}}$, there is a schedule $S_r=S_r(\beta )$ that makes $Q_{S_r}({\beta /2},\varepsilon )\le Q_{\text{coh}}({\beta /2},\varepsilon ,\alpha )$. Moreover, the maximal circuit depth required by fragmentation is asymptotically the same as that of probabilistic QITE.

Apart from the notable fact that fragmentation outperforms coherent QITE for both primitives, it is also remarkable that, long before $Q_{S_r}$ reaches $Q_{\text{coh}}$, at $\beta _{\text{c}}$, $Q_{S_r}$ is already much smaller than $Q_{\text{prob}}$. Crucially, these advantages of fragmented QITE come at no cost in circuit depth, since the query depth of fragmentation, $\sum _{l=1}^{r}\, q(\Delta \beta _l,\varepsilon ^{\prime }_l, \alpha _l)$, is observed to almost coincide with that of repeat until success, $q({\beta /2},\varepsilon ^{\prime }, \alpha )$, specially for high $\beta$. Note that the latter needs not be the case: strictly speaking, neither $q_1$ nor $q_2$ are additive in $\Delta \beta _l$ due to the non-linear dependance of $\varepsilon ^{\prime }_l$ on $\Delta \beta _l$.

Of course, the optimal schedules as functions of $\beta$ are a priori unknown. Nevertheless, the trends we observe for the schedule proposals in Eq. (7) are so compelling that they provide a sound basis for educated guesses in general:

#### Observation 7

(Optimal schedules). For uniform and non-uniform schedules $S_{r,1}$ and $S_{r,a}$, given by Eq. (7), the overall complexity for $P_1$ is respectively minimised by $r={\mathcal {O}}(\beta ^{1/2})$ and $(r,a)=\big ({\mathcal {O}}(1),{\mathcal {O}}(\beta ^{1/3})\big )$ (see Fig. 5); whereas for $P_2$ by $r=2$ and $(r,a)=\big ({\mathcal {O}}(1),{\mathcal {O}}(\beta ^{1/6})\big )$ (see^45^, Sec. XI).

As expected from the exponential dependence on *r* in Eq. (5), a slow growth of *r* with $\beta$ is observed for each *N* to minimize $Q_{S_r}({\beta /2},\varepsilon )$. This is indeed seen for $P_1$ with uniform schedules (Fig. 5, upper panel). On the other hand, for $P_2$ with uniform schedules, $r=2$ is observed^45^ to minimize $Q_{S_r}$ but the resulting complexity does not reach $Q_{\text{coh}}$ over the scanned domain ($0\le \beta \le 10000$). However, for both $P_1$ (Fig. 5, central panel) and $P_2$ with non-uniform schedules (where fragmentation does outperform amplitude amplification), the observed scaling of *r* is constant with both $\beta$ and *N*, remarkably. In turn, that *a* grows with $\beta$ implies that each $\Delta \beta _l$ decreases relative to $\Delta \beta _{l+1}$ as $\beta$ grows. This is consistent with the intuition from section "Fragmented master QITE algorithm" that each $\Delta \beta _l$ should be smaller than $\Delta \beta _{l+1}$. In addition, we consistently observe that, for the obtained optimal schedules, $\Delta \beta _1$ is only a tinny fraction (around 0.1% to 2%) of $8\ln (4/\varepsilon _1')$ (see^45^, Sec. IX). In fact, for both primitives, inserting the obtained $a(\beta )$ into Eq. (7), one sees that all $\Delta \beta _l$’s (except the last one, $\Delta \beta _r$) also decrease in absolute terms as $\beta$ grows. Yet, that *a* grows slowly with $\beta$ guarantees that the $\Delta \beta _l$’s do not decrease too much. More precisely, comparing with Eq. (5), we see that $\Delta \beta _l>\varepsilon '_l$ for all $l\in [r]$. This is an important sanity check, because if $\Delta \beta _l<\varepsilon '_l$, the identity operator would readily provide an $(\varepsilon '_l,1)$-block-encoding of $F_{\Delta \beta _l}(H)$, hence rendering the obtained scaling for $a(\beta )$ meaningless.

## Discussion

We have presented two QITE primitives and a master QITE algorithm. The first primitive is designed for block-encoding Hamiltonian oracles and has query complexity (number of oracle calls) sub-additive in the inverse-temperature $\beta$ and $\ln (\varepsilon ^{-1})$, with $\varepsilon$ the error. This scaling is better than all previously-known bounds^11,15^ for $\beta \lesssim 8\ln (4\,\varepsilon ^{-1})$ and becomes provably optimal for $\beta \ll \ln (\varepsilon ^{-1})$. Optimality is proven by showing saturation of a universal cooling-speed limit that is an imaginary-time counterpart of the celebrated no fast-forwarding theorem for real-time simulations^38–40^. It is an open question what the optimal scaling is away from the saturation regime. Coincidentally, the first steps of our master algorithm operate precisely in that regime. On the other hand, the second primitive is designed for a simplified model of real-time evolution oracles involving a single time. Its complexity is multiplicative in $\beta$ and $\ln (\varepsilon ^{-1})$, but it requires a single ancillary qubit throughout and its oracle is experimentally-friendlier than in previous QITE primitives. Interestingly, preliminary numerical analysis^46^ suggests that the asymptotic scaling with $\beta$ of both primitives’ complexities could actually be significantly better than in the analytical bounds above, for $P_1$ even reaching levels as good as $q_1(\beta ,\varepsilon ')={\mathcal {O}}\big (\sqrt{\beta \,\ln (1/\varepsilon ')}\big )$.

Our primitives are based on two technical contributions to quantum signal processing (QSP)^14,15,36,37^ relevant on their own. The first one is a bound on the approximation error of Hermitian-operator functions by their truncated Chebyshev series, for any analytical real function. The second one is a novel, Fourier-based QSP variant for real-time evolution oracles superior to previous ones^14^ in that it requires a single real time (and therefore a single ancilla), instead of multiple ones. Moreover, it is also experimentally friendly in that it requires no qubitization^37^.

Primitive technicalities aside, the main conceptual contribution of this work is the master QITE algorithm, which is conceptually simple, yet surprisingly powerful. It is based on breaking the evolution into small-$\beta$ fragments. This gives a large reduction in wasted queries and circuit depth, yielding an overall runtime competitive with (and for high $\beta$ even better than) that of coherent approaches based on quantum amplitude amplification (QAA). This is remarkable since the latter requires in general *N* extra ancillary qubits and circuits ${\mathcal {O}}\big (1/\sqrt{p_{\Psi }(\beta , \alpha )}\big )$ times deeper than the former. To put this in perspective, it is illustrative to compare with quantum amplitude estimation (QAE). In its standard form, QAE has similar hardware requirements as QAA^35^. However, recently, interesting algorithms have appeared^56,57^ that perform partial QAE with circuit depths that can interpolate between the probabilistic and coherent cases. In contrast, here, we beat full QAA using circuit depths for most runs much lower than in the bare probabilistic approach.

That fragmented QITE outperforms coherent QITE is proven rigorously for Primitive 1 and also supported by exhaustive numerical evidence for both primitives. Namely, our numerical experiments address random instances of Ising, transverse-field Ising, and Heisenberg-like Hamiltonians encoding computationally hard problems relevant for combinatorial optimisations, generative machine learning, and statistical physics, e.g. We emphasize that our analysis of is based on the analytical upper bounds on the query complexity we obtained, instead of the complexities themselves. The corresponding analysis for the actual (numerically obtained) query complexities requires re-optimizing the fragmentation schedules. Preliminary observations^46^ in that direction are again promising, indicating that the actual overall complexities may be orders of magnitude lower than in Fig. 3, e.g. In any case, qualitatively similar interplays between fragmentation and QAA are expected even for other types of primitives (beyond QITE) whose complexity and post-selection probability have similar scalings. All these exciting prospects are being explored for future work.

Our findings open a new research direction towards mid-term high-precision quantum algorithms. In particular, the presented primitives, cooling-speed limit, QSP methods, and master algorithm constitute a powerful toolbox for quantum signal processors specially relevant for the transition from NISQ to early prototypes of fault-tolerant hardware.

## Methods

### Preliminaries

We consider an *N*-qubit system ${\mathcal {S}}$, of Hilbert space ${\mathbb {H}}_{\mathcal {S}}$. QITE with respect to a Hamiltonian *H* on ${\mathbb {H}}_{\mathcal {S}}$ and over an imaginary time $-i\,\beta$ is represented by the non-unitary operator $e^{-\beta H}$. This can be simulated via post-selection with a unitary operator *U* that encodes $e^{-\beta H}$ in one of its matrix blocks^6,11,14,15,18^. We denote by ${\mathbb {H}}_{\mathcal {A}}$ the Hilbert space of an ancillary register ${\mathcal {A}}$, by ${\mathbb {H}}_\mathcal{S}\mathcal{A}={\mathbb {H}}_{\mathcal {S}}\otimes {\mathbb {H}}_{\mathcal {A}}$ the joint Hilbert space of ${\mathcal {S}}$ and ${\mathcal {A}}$, and by $\left\| A \right\|$ the spectral norm of an operator *A*. The following formalizes the encoding.

#### Definition 1

(*Block encodings*). For sub-normalization $0\le \alpha \le 1$ and tolerated error $\varepsilon > 0$, a unitary operator $U_A$ on ${\mathbb {H}}_\mathcal{S}\mathcal{A}$ is an $(\varepsilon ,\alpha )$-block-encoding of a linear operator *A* on ${\mathbb {H}}_{\mathcal {S}}$ if $\left\| \alpha \,A-\langle 0|\,U_A\,|0\rangle \right\| \le \,\varepsilon$, for some $|0\rangle \in {\mathbb {H}}_{\mathcal {A}}$. For $\varepsilon =0$ and $(\varepsilon ,\alpha )=(0,1)$ we use the short-hand terms perfect $\alpha$-block-encoding and perfect block-encoding, respectively.

E.g., if $U_A$ is a perfect $\alpha$-block-encoding of *A*, measuring $|0\rangle \in {\mathbb {H}}_{\mathcal {A}}$ on $U_A|\Psi \rangle |0\rangle \in {\mathbb {H}}_\mathcal{S}\mathcal{A}$, for any $|\Psi \rangle \in {\mathbb {H}}_{\mathcal {S}}$, leaves ${\mathcal {S}}$ in the state $\frac{A|\Psi \rangle }{\left\| A|\Psi \rangle \right\| }$. The probability of that outcome is $\alpha ^2\left\| A|\Psi \rangle \right\| ^{2}$. Note that, since $\left\| U_A \right\| =1$, a perfect $\alpha$-block-encoding is possible only if $\alpha \left\| A\right\| \le 1$. Hence, $\alpha$ allows one to encode matrices even if their norm is greater than 1. Typically, however, one wishes $\alpha$ as high as possible, to avoid unnecessary reductions in post-selection probability.

Our algorithms admit two types of oracle as input. The first one is based on perfect block-encodings of *H* and therefore requires $\left\| H\right\| \le 1$. If $\left\| H\right\| >1$, however, the required normalisation can be enforced by a simple spectrum rescaling. More precisely, for $\lambda _{-}$ and $\lambda _{+}$ arbitrary lower and upper bounds, respectively, to the minimal and maximal eigenvalues of *H*, $\lambda _{\text{min}}$ and $\lambda _{\text{max}}$, the rescaled Hamiltonian $H^{\prime }=\frac{H-{\bar{\lambda }}\mathbbm {1}}{\Delta \lambda }$ fulfils $\left\| H^{\prime }\right\| \le 1$ by construction, with the short-hand notation ${\bar{\lambda }}=\frac{\lambda _{+}+\lambda _{-}}{2}$ and $\Delta \lambda =\frac{\lambda _{+}-\lambda _{-}}{2}$. Then, by correspondingly rescaling the inverse temperature as $\beta ^{\prime }=\Delta \lambda \,\beta$, one obtains the propagator $e^{-\beta ^{\prime } H^{\prime }}$, which induces the same physical transformation as $e^{-\beta H}$. Hence, from now on, without loss of generality we assume throughout that $\left\| H\right\| \le 1$, i.e. that $-1\le \lambda _{\text{min}}\le \lambda _{\text{max}}\le 1$.

We are now in a good position to define our first oracle, $O_1$, which is the basis of our first primitive, $P_1$. We denote by ${\mathcal {A}}_{1}$ the entire ancillary register needed for $P_1$ and by ${\mathcal {A}}_{O_1}\subset {\mathcal {A}}_{1}$ the specific ancillary qubits required to implement $O_1$.

#### Definition 2

(*Block-encoding Hamiltonian oracles*). We refer as a block-encoding oracle for a Hamiltonian *H* on ${\mathbb {H}}_{\mathcal {S}}$ to a controlled unitary operator $O_1$ on ${\mathbb {H}}_{\mathcal{S}\mathcal{A}_{O_1}}$ of the form ${O_1=U_H\otimes |0\rangle \langle 0|+\mathbbm {1}\otimes |1\rangle \langle 1|}$, where $\mathbbm {1}$ is the identity operator on ${\mathbb {H}}_{{\mathcal {S}}}$, $\{|0\rangle ,|1\rangle \}$ a computational basis for the control qubit, and $U_H$ a perfect block encoding of *H*.

This is a powerful oracle paradigm used both in QITE^11,14,15,18^ and real-time evolution^15,36,37,58^. It encompasses, e.g., Hamiltonians given by linear combinations of unitaries, *d*-sparse Hamiltonians (i.e. with at most *d* non-null matrix entries per row), and Hamiltonians given by states^37^. Its complexity depends on *H*, but highly efficient implementations are known. E.g., for *H* a linear combination of *m* unitaries, each one requiring at most *c* two-qubit gates, $O_1$ can be implemented with $|{\mathcal {A}}_{O_1}|={\mathcal {O}}(\log _{2} m)$ ancillary qubits and gate complexity (i.e. total number of two-qubit gates) $g_{O_1}={\mathcal {O}}\big (m(c+\log _{2} m)\big )$^37,58^.

The second oracle model that we consider encodes *H* through the real-time unitary evolution it generates.

#### Definition 3

(*Real-time evolution Hamiltonian oracle*). We refer as a real-time evolution oracle for a Hamiltonian *H* on ${\mathbb {H}}_{\mathcal {S}}$ at a time $t\in {\mathbb {R}}$ to a controlled-$e^{-itH}$ gate $O_2=\mathbbm {1}\otimes |0\rangle \langle 0|+e^{-itH}\otimes |1\rangle \langle 1|$.

This is a simplified version of the models of^6,14^, e.g. There, controlled real-time evolutions at multiple times are required, thus involving multiple ancillas. In contrast, $O_2$ involves a single real time, so the ancillary register ${\mathcal {A}}_{O_2}$ consists of $|{\mathcal {A}}_{O_2}|=1$ single qubit (the control). In fact, we show below that no other ancilla is needed for our second primitive, $P_2$, i.e. ${\mathcal {A}}_{2}={\mathcal {A}}_{O_2}$. This is advantageous for near-term implementations. There, one may for instance apply product formulae^59,60^ to implement $O_2$ with gate complexities $g_{O_2}$ that, for intermediate-scale systems, can be considerably smaller than for $O_1$. Furthermore, this oracle is also relevant to hybrid analogue-digital platforms, for which QSP schemes have already been studied^61^.

QITE algorithms based on post-selection rely on a unitary quantum circuit to simulate a block encoding of the QITE propagator. We refer to such circuits as QITE primitives.

#### Definition 4

(*QITE primitives*). Let $\beta \ge 0$, $\varepsilon ^{\prime }\ge 0$, and $\alpha \le 1$. A $(\beta ,\varepsilon ^{\prime },\alpha )$-QITE-primitive of query complexity $q(\beta ,\varepsilon ^{\prime },\alpha )$ is a circuit *P*, with $q(\beta ,\varepsilon ^{\prime },\alpha )$ calls to an oracle *O* for *H* or its inverse $O^{\dagger }$, that generates an $(\varepsilon ^{\prime },\alpha )$-block-encoding $U_{F_{\beta }(H)}$ of $F_{\beta }(H)=e^{-\beta (H-\lambda _{\text{min}})}$, for all *H*.

Note that *P* is Hamiltonian agnostic, i.e. it admits any *H* provided it is properly encoded in the corresponding oracle. The factor $e^{-\beta \lambda _{\text{min}}}$ implies that $\Vert F_{\beta }(H)\Vert =1$, thus maximizing the post-selection probability. However, if $\lambda _{\text{min}}$ is unknown, one can replace it by a suitable lower bound $\lambda _{-}\ge -1$ . This introduces only a constant sub-normalisation. In turn, the query complexity is the gold-standard figure of merit for efficiency of oracle-based algorithms. It quantifies the runtime of *P* relative to that of an oracle query. In fact, *P* is time-efficient if its query complexity and gate complexity per query $g_P$ are both in ${\mathcal {O}}\big (\text{poly}(N, \beta ,1/\varepsilon ^{\prime },\alpha )\big )$.

Importantly, normalisation causes the post-selection probability $p_{\Psi }(\beta ,\varepsilon ^{\prime }, \alpha )$ of *P* (on an input state $|\Psi \rangle$) to propagate onto the error $\varepsilon$ in the output state, making the latter in general greater than $\varepsilon ^{\prime }$. The exact dependence of $\varepsilon$ on $\varepsilon ^{\prime }$ is dictated by $p_{\Psi }(\beta ,\varepsilon ^{\prime }, \alpha )$. However, if $\varepsilon ^{\prime }\le \,\varepsilon \, \sqrt{p_{\Psi }(\beta , \alpha )}/2$, with $p_{\Psi }(\beta , \alpha )=p_{\Psi }(\beta ,0, \alpha )=\alpha ^2\, \left\| F_{\beta }(H)|\Psi \rangle \right\| ^2$, the output-state error is ${\mathcal {O}}(\varepsilon )$ (Sup. Mat.^45^, Sec. II), with $``{\mathcal {O}}(\cdot )''$ standing for “asymptotically upper-bounded by”. In turn, the primitives must be incorporated into master algorithms which we formaly define below.

#### Definition 5

(*Master QITE algorithms*). Given $\varepsilon \ge 0$, $\beta \ge 0$, $|\Psi \rangle \in {\mathbb {H}}_{\mathcal {S}}$, and $(\beta ^{\prime },\varepsilon ^{\prime },\alpha ^{\prime })$-QITE-primitives $P_{\beta ^{\prime },\varepsilon ^{\prime },\alpha ^{\prime }}$ querying oracles for a Hamiltonian *H*, a $(\beta ,\varepsilon )$-master-QITE-algorithm for *H* on $|\Psi \rangle$ is a procedure that outputs the state $\frac{F_{\beta }(H)|\Psi \rangle }{\left\| F_{\beta }(H)|\Psi \rangle \right\| }$ up to trace-distance error $\varepsilon$ with unit probability. Its overall query complexity $Q(\beta ,\varepsilon )$ is the sum over the query complexities of each $P_{\beta ^{\prime },\varepsilon ^{\prime },\alpha ^{\prime }}$ applied.

### Quantum signal processing

Quantum signal processing (QSP) is a powerful method to obtain an $\varepsilon ^\prime$-approximate block encoding of an operator function $f(H)=\sum _{\lambda }f(\lambda )|\lambda \rangle \langle \lambda |$, where $\{|\lambda \rangle \in {\mathbb {H}}_{\mathcal {S}}\}$ are the eigenvectors and $\{\lambda \}$ the eigenvalues of a Hamiltonian *H*, from queries to an oracle for *H*^36^. We note that QSP can also be extended to non-Hermitian operators^15^, but here we restrict to the Hermitian case for simplicity. We present two QSP methods for general functions one for each oracle model in Defs. 2 and 3. Our QITE primitives are then obtained by particularizing these methods to the case $f(H)=F_{\beta }(H)$, with $F_{\beta }(H)=e^{-\beta (H-\lambda _{\text{min}})}$.

#### Real-variable function design with single-qubit rotations

We start by reviewing how to approximate functions of one real variable with single-qubit pulses.

**Single-qubit QSP method 1.** Consider the single qubit rotation $R_1(\theta ,\phi )=e^{i\theta {X}}e^{i\phi {Z}}$, where *X* and *Z* are the first and third Pauli matrices, respectively, and $\phi \in [0,2\pi ]$. The angle $\theta \in [-\pi ,\pi ]$ is the signal to be processed and the rotation $e^{i\theta {X}}$ is called the iterate. One can show^62^ that, given $q\in {\mathbb {N}}_{\text {even}}$ and a sequence of angles ${\varvec{\Phi }_1}=\big (\phi _{1},\cdots ,\phi _{q+1}\big )\in {\mathbb {R}}^{q+1}$, the sequence of rotations ${\mathcal {R}}_1\left( \theta ,{\varvec{\Phi }_1}\right) =e^{i\phi _{q+1}{Z}}\prod _{k=1}^{q/2}R_1(-\theta ,\phi _{2k})R_1(\theta ,\phi _{2k-1})$ has matrix representation in the computational basis

8 $$\begin{aligned} {\mathcal {R}}_1\left( \theta ,{\varvec{\Phi }_1}\right) =\left( \begin{array}{cc} B(\cos \theta ) &{} i\,\sin \theta \,D(\cos \theta )\\ i\,\sin \theta \,D^*(\cos \theta ) &{} B^{*}(\cos \theta ) \end{array}\right) , \end{aligned}$$

where *B* and *D* are polynomials in $\cos \theta$ with complex coefficients determined by $\varvec{\Phi }_1$.

For target real polynomials ${\mathscr {B}}(\cos \theta )$ and ${\mathscr {D}}(\cos \theta )$, we wish to find ${\varvec{\Phi }_1}$ that generates $B(\cos \theta )$ and $D(\cos \theta )$ with ${\mathscr {B}}(\cos \theta )$ and ${\mathscr {D}}(\cos \theta )$ as either their real or imaginary parts, respectively. This can be done iff they satisfy^45^

9 $$\begin{aligned} {\mathscr {B}}^{2}(\cos \theta )+\sin ^2\theta \,{\mathscr {D}}^{2}(\cos \theta )\le 1 \end{aligned}$$

for all $\theta$, and have the form

10 $$\begin{aligned} \begin{aligned} {\mathscr {B}}(\cos \theta )&=\sum _{k=0}^{q/2}b_k\cos (2k\theta )\\ \sin \theta \,{\mathscr {D}}(\cos \theta )&=\sum _{k=1}^{q/2} d_{k}\sin \left( 2k\theta \right) , \end{aligned} \end{aligned}$$

with $b_k\in {\mathbb {R}}$ and $d_k\in {\mathbb {R}}$. Alternatively, Eq. (10) can also be expressed in terms of Chebyshev polynomials of first $T_{k}(\cos \theta )=\cos (k\theta )$ and second $U_{k}(\cos \theta )=\sin \left( (k+1)\theta \right) /\sin \theta$ kinds. This can be used to obtain either Chebyshev or Fourier series of target operator functions. If the target expansion satisfies Eqs. (9) and (10), the angles ${\varvec{\Phi }_1}$ can be computed classically in time ${\mathcal {O}}\left( \text {poly}(q)\right)$^62–65^.

**Single-qubit QSP method 2.** This method is inspired by a construction in Ref.^66^ and shown in detail in a companying paper^47^. The fundamental gate is $R_2(x,\omega ,\zeta ,\eta ,\varphi ,\kappa )=e^{i\frac{\zeta +\eta }{2} Z}e^{-i\varphi Y}e^{i\frac{\zeta -\eta }{2} Z}e^{i\omega x Z}e^{-i\kappa Y}$, which has five adjustable parameters $\{\omega ,\varvec{\xi }\}\in {\mathbb {R}}^5$, where $\varvec{\xi }=\{\zeta ,\eta ,\varphi ,\kappa \}$. Here, $x\in {\mathbb {R}}$ will play the role of the signal and $e^{i \omega x Z}$ that of the iterate. In Ref.^66^, it was observed that the gate sequence ${\mathcal {R}}_2(x,\varvec{\omega },\varvec{\Phi }_2)=\prod _{k=0}^{q}R_2(x,\omega _k,\varvec{\xi }_k)$, with $\varvec{\omega }=\{\omega _0,\cdots , \omega _q\}\in {\mathbb {R}}^{q+1}$ and $\varvec{\Phi }_2=\{\varvec{\xi }_0,\cdots ,\varvec{\xi }_{q}\}\in {\mathbb {R}}^{4(q+1)}$, can encode certain finite Fourier series into its matrix components. In^47^, not only it is formally proven that for any target series a unitary operator can be built with it as one of its matrix elements but also we provide an explicit, efficient recipe for finding the adequate choice of pulses $\varvec{\Phi }_2$. This is the content of the following lemma.

##### Lemma 8

(Single-qubit Fourier series synthesis) Given ${\tilde{g}}_q(x)=\sum _{m=-q/2}^{q/2} c_m\, e^{im x}$, with $q\in {\mathbb {N}}$ even, there exist $\varvec{\omega }$ and $\varvec{\Phi }_2$ such that $\langle 0|\,{\mathcal {R}}_2(x,\varvec{\omega },\varvec{\Phi }_2)\,|0\rangle ={\tilde{g}}_q(x)$ for all $|x|\le \pi$ iff $|{\tilde{g}}_q(x)|\le 1$ for all $|x|\le \pi$. Moreover, $\omega _0=0$ and $\omega _k=(-1)^k/2$, for all $1\le k\le q$, and $\varvec{\Phi }_2$ can be calculated classically from $\{c_m\}_{m}$ in time ${\mathcal {O}}\left( \text {poly}(q)\right)$.

#### Operator-function design from block-encoded oracles

Here, we synthesize an ($\varepsilon '$,1)-block-encoding of *f*(*H*) from queries to an oracle for *H* as in Defenition 2. The algorithm can be seen as a variant of the single-ancilla method from Ref.^37^ with slightly different pulses. The basic idea is to design a circuit, $P_1$, that generates a perfect block-encoding $V_{\varvec{\Phi }_1}$ of a target Chebyshev expansion ${\tilde{f}}_q(H)=\sum ^{q/2}_{k=0} b_k \,T_k(H)$ that $\varepsilon _\text {tr}$-approximates *f*(*H*), for some $0\le \varepsilon _\text {tr} \le \varepsilon '$. This can be done by adjusting $\varvec{\Phi }_1$ as in section "Quantum signal processing". Note that the achievability condition (9) requires that $\Vert {\tilde{f}}_q(H)\Vert \le 1$, but we only guarantee $\Vert {\tilde{f}}_q(H)\Vert \le 1+\varepsilon _\text {tr}$. However, this can be easily accounted for introducing an inoffensive sub-normalization $\alpha =(1+\varepsilon _\text {tr})^{-1}$ (see, e.g., Lemma 14 in Ref.^37^), which we neglect here throughout. Choosing ${\tilde{f}}_q$ as the truncated Chebyshev series of *f* with truncation error $\varepsilon _\text {tr} \le \varepsilon '$, we obtain the desired block-encoding of *f*(*H*). For *f* analytical, the error fulfills^67^

11 $$\begin{aligned} \varepsilon _\text {tr}\le \frac{\underset{\lambda \in [\lambda _{\text {min}},\lambda _{\text {max}}]}{\max }\left| f^{(q/2+1)}(\lambda )\right| }{2^{\frac{q}{2}}(q/2+1)!}, \end{aligned}$$

with $f^{(q/2+1)}$ the $(q/2+1)$-th derivative of *f*. This allows one to obtain the truncation order *q*/2 and Chebyshev coefficients ${\varvec{b}}=\{b_k\}_{0\le k\le q/2}$ (see^45^, Sec. XIII). Then, from ${\varvec{b}}$, one can calculate the required $\varvec{\Phi }_1$ (see^45^, Sec. XIV).

Next, we explicitly show how to generate $V_{\varvec{\Phi }_1}$. Using the short-hand notation $|0_{\lambda }\rangle =|\lambda \rangle |0\rangle \in {\mathbb {H}}_{\mathcal{S}\mathcal{A}_{O_1}}$ and Defenition 2, one writes $O_1|0_{\lambda }\rangle =\lambda |0_{\lambda }\rangle +\sqrt{1-\lambda ^{2}}|0_{\lambda }^{\perp }\rangle$ with $\langle 0_{\lambda }|0_{\lambda }^{\perp }\rangle =0$. This defines the 2-dimensional subspace ${\mathbb {H}}_\lambda =\text {span}\{|0_{\lambda }\rangle ,|0_{\lambda }^{\perp }\rangle \}$. To exploit the single-qubit formalism from section "Quantum signal processing", one needs an iterate that acts as an *SU*(2) rotation within each ${\mathbb {H}}_\lambda$. In general, $O_1$ itself is not appropriate for this due to leakage out of ${\mathbb {H}}_\lambda$ by repeated applications of $O_1$. However, there is a simple oracle transformation—qubitization—that maps $O_1$ into another block-encoding $O'_1$ of the same *H* but with the desired property^37^. The transformed oracle reads^45^

12 $$\begin{aligned} O'_1 =\bigoplus _{\lambda }e^{-i\theta _\lambda {Y}_\lambda }, \end{aligned}$$

with $$\theta _\lambda {:}=\cos ^{-1}(\lambda )$$ and $$Y_\lambda =i(|0_{\lambda }^{\perp }\rangle \langle 0_{\lambda }| - |0_{\lambda }\rangle \langle 0_{\lambda }^{\perp }|)$$.

**Figure 6:**
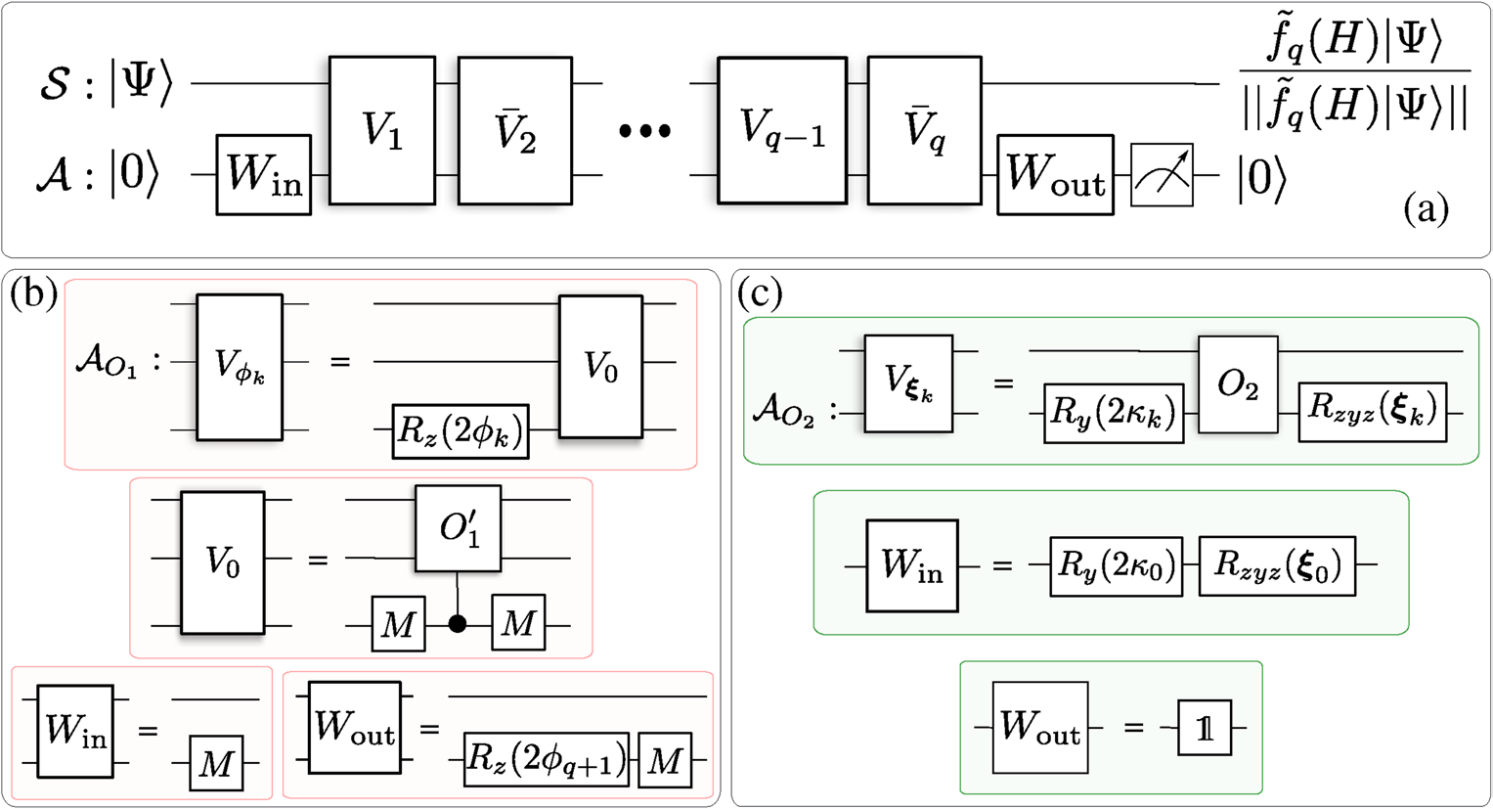
QSP primitives for generic operator function design. (**a**) Both circuits $P_1$ from Algorithm 2 and $P_2$ from Algorithm 3 have the same structure. If the ancillas are initialised and post-selected in $|0\rangle _{{\mathcal {A}}}$, the circuit prepares the system state $\frac{{\tilde{f}}_q(H)|\Psi \rangle }{||{\tilde{f}}_q(H)|\Psi \rangle ||}$, which $\varepsilon$-approximates the target output $\frac{f(H)|\Psi \rangle }{\Vert f(H)|\Psi \rangle \Vert }$. The details specific to $P_1$ and $P_2$ are respectively shown in panels (**b**) and (**c**). $W_{\text {in}}$ and $W_{\text {out}}$ are fixed ancillary unitaries, and *M* is a single-qubit Hadamard gate. The basic blocks $V_k$ in panel a) represent the gates $V_{\phi _k}$ in b) and $V_{\varvec{\xi }_k}$ in c). Each $V_{\phi _k}$ involves one query to the qubitized oracle $O'_1$, which in turn requires one query to $O_1$ and one to its inverse $O^{\dagger }_1$ (Sup. Mat.^45^, Fig. S8). Whereas each $V_{\varvec{\xi }_k}$ involves one query to the oracle $O_2$. ${\bar{V}}_k$ is defined as $V_k$ but with $O_1^{\prime \dagger }$ substituting $O'_1$ or $O_2^{\dagger }$ substituting $O_2$. Hence, the query complexities of $P_1$ and $P_2$ are respectively 2*q* and *q*. The approximating function ${\tilde{f}}_q$ is determined by the angles ${\varvec{\Phi }_1}=\big (\phi _{1},\cdots ,\phi _{q+1}\big )$ or $\varvec{\Phi }_2=\{\varvec{\xi }_0,\cdots ,\varvec{\xi }_{q}\}$ in the rotations $R_z(2\phi _k)=e^{i\phi _k{Z}}$ or $R_y(2\kappa _k)=e^{i\kappa _k Y}$ and $R_{zyz}(\varvec{\xi }_k)=R_z(\zeta _k+\eta _k)\,R_y(2\varphi _k)\,R_z(\zeta _k-\eta _k)$, with $\varvec{\xi }_k=\{\zeta _k,\eta _k,\varphi _k,\kappa _k\}$. For $P_1$ and $P_2$, these angles are chosen such that ${\tilde{f}}_q$ is a high-precision Chebyshev and Fourier approximation of *f*, respectively.

Although the qubit resemblance could be considered in a direct analogy to QSP for a single qubit, it leads to a more strict class of achievable functions than if we resort to one additional qubit (single-ancilla QSP)^37^. This extra ancilla controls the action of the oracle $O'_1$ through the iterate

13 $$\begin{aligned} V_{0}=\mathbbm {1}\otimes |+\rangle \langle +|+O'_1\otimes |-\rangle \langle -| \end{aligned}$$

on ${\mathbb {H}}_{\mathcal{S}\mathcal{A}}$, where $|\pm \rangle$ are the eigenstates of the Pauli operator *X* for the QSP qubit ancilla. Throughout this section, $\mathbbm {1}$ is the identity operator on ${\mathbb {H}}_{\mathcal{S}\mathcal{A}_{O_1}}$ and *M* denotes the single-qubit Hadamard gate. Let us define the operators $V_{\phi }=V_{0}\left( \mathbbm {1}\otimes e^{i\phi {Z}}\right)$ and ${\bar{V}}_{\phi }=V_{0}^\dagger \left( \mathbbm {1}\otimes e^{i\phi {Z}}\right)$ for a given phase $\phi \in [0,2\pi ]$, which play the role of $R(\theta ,\phi )$ of the previous sub-section with $\theta _\lambda$ playing the role of $\theta$ for each $\lambda$. These operators can be phase iterated to generate

14 $$\begin{aligned} V_{{\varvec{\Phi }_1}}=W_{\text {out}}\left( {\bar{V}}_{\phi _{q}}{V}_{\phi _{q-1}}\cdots {\bar{V}}_{\phi _{2}}{V}_{\phi _{1}} \right) W_{\text {in}} \end{aligned}$$

on ${\mathbb {H}}_{\mathcal{S}\mathcal{A}}$, with ancilla pre- and post-processing unitaries $W_{\text {in}}=\mathbbm {1}\otimes M$ and $W_{\text {out}}: = \mathbbm {1}\otimes \big (M \,e^{i\phi _{q+1}{Z}}\big )$, respectively, with *M* the single-qubit Hadamard matrix. The resulting circuit, $P_1$, is depicted in Fig. 6a,b.

The following pseudocode gives the entire procedure.

**Algorithm 2:**
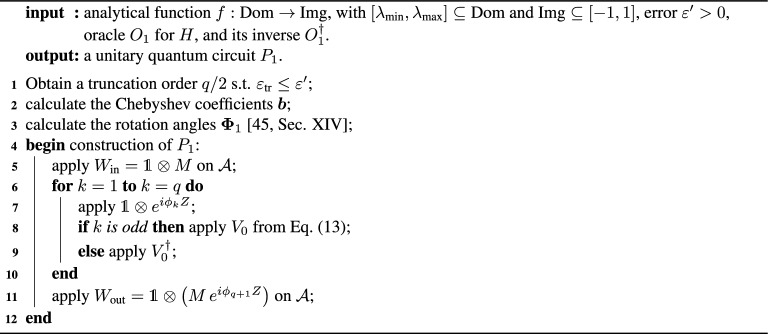
Operator-function design from block-encoded Hamiltonian oracles.

The correctness and complexity of Algorithm 2 are addressed by the following lemma, proven in^45^ (Sec. XVI).

##### Lemma 9

Let *f*, $\varepsilon '$, $O_1$, and $O^{\dagger }_1$ be as in the input of Algorithm 2. Then, for any *q* s.t. $\varepsilon _\text {tr}$ in Eq. (11) is no greater than $\varepsilon '$, there exists $\varvec{\Phi }_1\in {\mathbb {R}}^{q+1}$ such that $V_{\varvec{\Phi }_1}$ in Eq. (14) is a ($\varepsilon ^\prime$,1)-block-encoding of *f*(*H*). The circuit $P_1$ generating $V_{\varvec{\Phi }_1}$ requires a single-qubit ancilla, *q* queries to $O_1$ and $O^{\dagger }_1$ each, and $g_{P_1}={\mathcal {O}}\left( g_{O_1}+|{\mathcal {A}}_{O_1}|\right)$ gates per query, with $g_{O_1}$ the gate complexity of $O_1$. Furthermore, the classical runtime (calculations of ${\varvec{b}}$ and $\varvec{\Phi }_1$) is within complexity ${\mathcal {O}}\big (\text{poly}(q/2)\big )$.

Some final comments about the input function are in place. The restriction of *f* being analytical is needed to determine the truncation order through Eq. (11). In fact, to evaluate the RHS of the equation exactly, one needs in general closed-form expression for *f*. However, if the required truncation order is given in advance, the corresponding Chebyshev coefficients can be obtained from $q/2+1$ evaluations of *f* in specific points (the nodes of the Chebyshev polynomials). In that case, a closed-form expression for *f* is not required and a classical oracle for evaluating it suffices. Moreover, it is important to note that a satisfactory Chebyshev approximation is guaranteed to exist for all bounded and continuous functions^68^. If the Chebyshev expansion is given, then step 1 of Algorithm 2 can obviously be skipped and *f* is not required at all. We further note that Algorithm 2 can also be applied even to non-continuous functions over restricted domains without the discontinuities. This is for instance the case of the inverse function, which can be well-approximated over the sub-domain $[-1,-\delta ]\cup [\delta ,1]$ by a pseudo-inverse polynomial of $\delta$-dependent degree^69^.

**QITE-primitive from a block-encoding oracle.** QITE primitive 1 corresponds to the output of Algorithm 2 for $f(\lambda )=F_{\beta }(\lambda )=e^{-\beta (\lambda -\lambda _{\text{min}})}$. The Chebyshev coefficients can be readily obtained from the Jacobi–Anger expansion^70^

15 $$\begin{aligned} e^{-\beta \lambda }=I_{0}(\beta )+2\sum _{k=1}^{\infty }(-1)^{k}I_{k}(\beta )\,T_{k}({\lambda }), \end{aligned}$$

where $I_{k}(\beta )$ is a modified Bessel function. The proof of Theorem 1 thus follows straightforwardly from Lemma 9.

##### Proof of Theorem 1

The function $F_{\beta }:[\lambda _{\text{min}},\lambda _{\text{max}}]\rightarrow (0,1]$, with $F_{\beta }(\lambda )=e^{-\beta (\lambda -\lambda _{\text{min}})}$ for all $\lambda \in [\lambda _{\text{min}},\lambda _{\text{max}}]$ satisfies all the assumptions of Lemma 9. Hence, on input $f=F_{\beta }$, Algorithm 2 outputs an $(\beta ,\varepsilon ^\prime ,1)$-QITE-primitive. By Eq. (11), the corresponding truncation error is ($q'=q/2$)

16 $$\begin{aligned} \varepsilon _\text {tr}\le \frac{\beta ^{q'+1}}{2^{q'}(q'+1)!}&\le \sqrt{\frac{2}{\pi (q'+1)}}\left( \frac{e\beta }{2(q'+1)}\right) ^{q'+1}\nonumber \\&\le \left( \frac{\beta e}{2q'}\right) ^{q'}, \end{aligned}$$

where Stirling inequality has been invoked and we assumed $e\beta /2\le q'$. (We note also that the first inequality can also be obtained from explicit summation using Eq. (15) and the properties of the Bessel functions^71^.) Then, imposing $\big (\frac{\beta e}{2q'}\big )^{q'}\le \varepsilon ^\prime$ and solving for $q'$^15^ gives the query complexity of Eq. (1). $\square$

Primitive 1 is based on the Jacobi–Anger expansion^70^. This gives a Chebyshev-polynomial series^67,68^ for the exponential function, which can be synthesized with quantum signal processing (see section "Quantum signal processing"). The expansion has been applied to real-time evolution^15,36,37,40^ and even to the QITE propagator $F_{\beta }(H)$^18^, for partition function estimation. However, the algorithm from^18^ performs only a statistical simulation of $F_{\beta }(H)$ based on post-processing and hence cannot simulate QITE on states. In particular, it cannot be used for Gibbs-state sampling, e.g. Moreover, the query complexity from^18^ is ${\mathcal {O}}\big (N+ \beta + \ln (1/\varepsilon ')\big )$, which is worse than Eq. (1) in that it contains the extra term *N* and lacks the denominator in the second term of Eq. (1). Traditionally^40^, the truncation error in the expansion is bounded using properties of the Bessel functions^71^. In contrast, here, we use a generic upper bound (Lemma 9, in Methods) for arbitrary Hermitian-operator functions. This gives the same bound as^40^ for the exponential but holds for any analytical real function, hence being useful in general.

A further remark about the query complexity of P$_1$. The solution for $q'$ satisfying $\big (\frac{\beta e}{2q'}\big )^{q'}\le \varepsilon ^\prime$ given in Ref.^15^ is based on upperbounds for $q'$ in two regimes. When $\beta \ge 2 \ln (1/\varepsilon ')/e^2$, it is shown that $q'\le e^2\beta /2$. On the other hand, it applies that $q'\le 4\ln (1/\varepsilon ')/\ln (e+2\ln (1/\varepsilon ')/(e\beta ))$ for $\beta \le 2 \ln (1/\varepsilon ')/e^2$. Therefore, for any $\beta$,

17 $$\begin{aligned} q=2q'\le 8\left[ \frac{e\beta }{2}+\frac{\ln (1/\varepsilon ')}{\ln (e+2\ln (1/\varepsilon ')/(e\beta ))}\right] \end{aligned}$$

is a valid upperbound for the query complexity. Consequently, the muliplicative factor implied by the ${\mathcal {O}}()$ notation in Eq. (1) is known and modestly equal to 8.

#### Operator function design from real-time evolution oracles

Here, we synthesize an ($\varepsilon ^\prime ,\alpha$)-block-encoding of *f*(*H*) from an oracle for *H* as in Defenition 3. We proceed as in section "Quantum signal processing", but with a circuit $P_2$ generating a perfect block-encoding $V_{\varvec{\Phi }_2}$ of a target Fourier expansion ${\tilde{g}}_q(H)=\sum _{m=-q/2}^{q/2} c_m e^{imHt}$ that $\varepsilon _{\text {tr}}$-approximates $\alpha \, f(H)$, for some $\varepsilon _{\text {tr}}\le \varepsilon '$, $\alpha \le 1$, and a suitable $t>0$. This is done by adjusting $\varvec{\Phi }_2$ according to Lemma 8. The function ${\tilde{g}}_q$ is a Fourier approximation of an intermediary function *g* such that $g(\lambda ,t)=g(x_{\lambda })=\alpha \, f(\lambda )$, for *t* chosen so that $x_{\lambda }=\lambda \, t$ is in the interval of convergence of ${\tilde{g}}_q$ to *g* for all $\lambda \in [\lambda _{\text {min}},\lambda _{\text {max}}]$. The reason for this intermediary step here is to circumvent the well-known Gibbs phenomenon, by virtue of which convergence of a Fourier expansion cannot in general be guaranteed at the boundaries. In turn, the sub-normalization factor $\alpha$ arises because our ${\tilde{g}}_q$ converges to *g* only for $|x_{\lambda }|<{\pi /2}$, whereas Lemma 8 requires that $|{\tilde{g}}_q(x_{\lambda })|\le 1$ for all $|x_{\lambda }|\le \pi$. This forces one to sub-normalize the expansion so as to guarantee normalization over the entire domain. (As in section "Quantum signal processing", the inoffensive sub-normalization factor $(1+\varepsilon _\text {tr})^{-1}$ is neglected.)

More precisely, we employ (see Ref.^47^) a construction from Ref.^14^ that, given $0<\delta \le \pi /2$ and a power series that $\frac{\varepsilon _{\text {tr}}}{4}$-approximates *g*, gives ${\varvec{c}}=\{c_m\}_{|m|\le q/2}$ such that ${\tilde{g}}_q$ $\varepsilon _{\text {tr}}$-approximates *g* for all $x_{\lambda }\in [-\pi /2+\delta ,\pi /2-\delta ]$, if

18 $$\begin{aligned} q\ge \Bigg \lceil \frac{2\pi }{\delta }\ln \left( \frac{4}{\varepsilon _{\text {tr}}}\right) \Bigg \rceil . \end{aligned}$$

For *f* analytical, one can obtain the power series of *g* from a truncated Taylor series of *f* using that $g(x_{\lambda })=\alpha f(\lambda )$. The truncation order *L* can be obtained from the remainder:

19 $$\begin{aligned} \frac{\varepsilon _\text {tr}}{4}\le \frac{\underset{\lambda \in [\lambda _{\text {min}},\lambda _{\text {max}}]}{\max }\left| \alpha \, f^{(L+1)}(\lambda )\right| }{(L+1)!}. \end{aligned}$$

In turn, the conditions $[\lambda _{\text {min}},\lambda _{\text {max}}]\subseteq [-1,1]$ and $x_{\lambda }\in [-\pi /2+\delta ,\pi /2-\delta ]$ lead to the natural choice $t=\pi /2-\delta$. (This renders ${\tilde{g}}_q$ periodic in $x_{\lambda }$ with period $2\pi$.) In addition, in Ref.^47^ the sub-normalization constant $\alpha$ is bounded in terms of the obtained *t* and Taylor coefficients ${\varvec{a}}=\{a_l\}_{0\le l\le L}$ of *f*. It suffices to take $\alpha$ such that

20 $$\begin{aligned} \sum _{l=0}^L\big |a_l/(1-2\,\delta /\pi )^l\big |\le \alpha ^{-1}. \end{aligned}$$

Note that *L* and $\alpha$ are inter-dependent. One way to determine them is to increase *L* and iteratively adapt $\alpha$ until Eqs. (19) and (20) are both satisfied. Alternatively, if the expansion converges sufficiently fast (e.g., if $\lim _{l\rightarrow \infty }|\frac{a_{l+1}}{a_l}|<1-\frac{2\delta }{\pi }$), one can simply substitute *L* in Eq. (20) by $\infty$. This is indeed the case with QITE primitives. There, the substitution introduces a slight increase of unnecessary sub-normalization but makes the resulting $\alpha$ independent of *L*, thus simplifying the analysis. Then, from the obtained ${\varvec{c}}$, one can finally calculate the required $\varvec{\Phi }_2$^47^.

Next, we explicitly show how to generate $V_{\varvec{\Phi }_2}$. The iterate is now taken simply as the oracle itself: $O_2=\mathbbm {1}\otimes |0\rangle \langle 0|+ e^{-iHt}\otimes |1\rangle \langle 1|$. Notice that, in contrast to $O_1$, $O_2$ readily acts as an *SU*(2) rotation on each 2-dimensional subspace $\text {span}\{|\lambda \rangle |0\rangle ,|\lambda \rangle |1\rangle \}$. This relaxes the need for qubitization. The basic QSP blocks for the unitary operator $V_{{\varvec{\Phi }_2}}=W_{\text {out}}\left( {\bar{V}}_{\varvec{\xi }_{q}}{V}_{\varvec{\xi }_{q-1}}\cdots {\bar{V}}_{\varvec{\xi }_{2}}{V}_{\varvec{\xi }_{1}} \right) W_{\text {in}}$ are

21a $$\begin{aligned} {V}_{\varvec{\xi }_k}=\left[ \mathbbm {1}\otimes \left( e^{i\frac{\zeta _k+\eta _k}{2} Z}e^{-i\varphi _k Y}e^{i\frac{\zeta _k-\eta _k}{2} Z}\right) \right] O_2 \left[ \mathbbm {1}\otimes e^{-i\kappa _k Y}\right] , \end{aligned}$$

and

21b $$\begin{aligned} {\bar{V}}_{\varvec{\xi }_k}=\left[ \mathbbm {1}\otimes \left( e^{i\frac{\zeta _k+\eta _k}{2} Z}e^{-i\varphi _k Y}e^{i\frac{\zeta _k-\eta _k}{2} Z}\right) \right] O_2^\dagger \left[ \mathbbm {1}\otimes e^{-i\kappa _k Y}\right] \end{aligned}$$

with ${\varvec{\xi }_k}=\{\zeta _k,\eta _k,\varphi _k,\kappa _k\}$. $V_{\varvec{\xi }_k}$ and ${\bar{V}}_{\varvec{\xi }_k}$ play a similar role to $R_2(x,\omega _k,\varvec{\xi }_k)$ in section "Quantum signal processing" (with $x_\lambda$ inside $O_2$ playing the role of *x* there for each $\lambda$). Here we take

22 $$\begin{aligned} W_{\text {in}}=\mathbbm {1}\otimes \big [e^{i\frac{\zeta _0+\eta _0}{2} Z}e^{-i\varphi _0 Y}e^{i\frac{\zeta _0-\eta _0}{2} Z}e^{-i\kappa _0 Y}\big ] \end{aligned}$$

and $W_{\text {out}}=\mathbbm {1}$. The circuit is depicted in Fig. 6a,c.

The following pseudocode presents the entire procedure.

**Algorithm 3:**
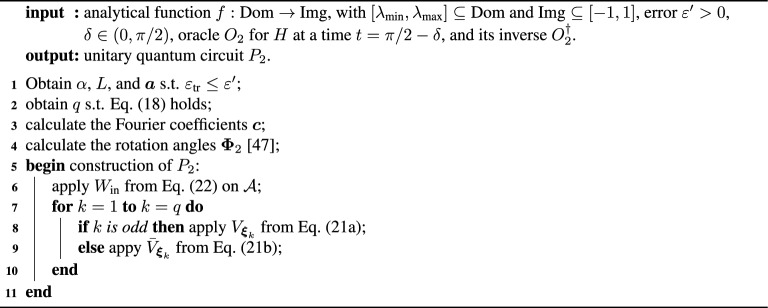
Operator-function design from real-time evolution Hamiltonian oracles.

The correctness and complexity of Algorithm 3 are addressed by the following lemma, proved in Ref.^47^.

##### Lemma 10

Let *f*, $\varepsilon '$, $\delta$, $O_2$, and $O_2^\dagger$ be as in the input of Algorithm 3. Then, for any *q* satisfying Eq. (18) and $\alpha$ satisfying Eq. (20), there exists $\varvec{\Phi }_2\in {\mathbb {R}}^{4(q+1)}$ such that $V_{\varvec{\Phi }_2}$ is an ($\varepsilon ^\prime ,\alpha$)-block-encoding of *f*(*H*). The circuit $P_2$ generating $V_{\varvec{\Phi }_2}$ requires *q*/2 queries to $O_2$ and $O_2^\dagger$ each and $4+g_{O_2}$ gates per query, with $g_{O_2}$ the gate complexity of $O_2$. Moreover, the classical runtime is within complexity ${\mathcal {O}}\big (\text {poly}(L,q/2)\big )$.

Clearly, if a suitable power series for *f* is a-priori available, analyticity of *f* is not required and step 1 in Algorithm 3 can be skipped. Finally, we note that it is always possible to avoid sub-normalization by introducing a periodic extension of *f* that is readily normalized over the entire domain of the Fourier expansion. However, Eq. (18) is then no longer valid and one must assess the query complexity on a case-by-case basis. This can for instance be tackled numerically by variationally optimising the gate sequence ${\mathcal {R}}_2(x,\varvec{\omega },\varvec{\Phi }_2)$ to block-encode the periodic extension of *f*^66^. Either way, clearly, if a normalized Fourier expansion is a-priori available, one can skip steps 1 to 3 in Algorithm 3.

**QITE-primitive from a real-time evolution oracle.** QITE primitive 2 is the output of Algorithm 3 for $f=F_{\beta }$. The proof of Theorem 2 thus follows straight from Lemma 10.

##### Proof of Theorem 2

The function $F_\beta :[\lambda _{\text {min}},\lambda _{\text {max}}]\rightarrow (0,1]$, with $F_{\beta }(\lambda )=e^{-\beta (\lambda -\lambda _{\text{min}})}$, satisfies the assumptions of Lemma 10 with *L* given by Eq. (19) for $\max _{\lambda \in [\lambda _{\text {min}},\lambda _{\text {max}}]}\big |\alpha \, f^{(L+1)}(\lambda )\big |=\alpha \,\beta ^{L+1}$. Its Taylor coefficients are $a_l=\frac{e^{\beta \lambda _{\text{min}}}(-\beta )^l}{l!}$, for all $l\in {\mathbb {N}}$. To obtain $\alpha$, we note that $\sum _{l=0}^{L}|a_l/\big (1-\frac{2\delta }{\pi }\big )^l|\le \sum _{l=0}^{\infty }|a_l/\big (1-\frac{2\delta }{\pi }\big )^l|= e^{\beta \lambda _{\text{min}}} e^{\frac{\beta }{1-2\delta /\pi }}$. Hence, by Eq. (20), we can take $\alpha =e^{-\beta (\lambda _{\text{min}}+\frac{1}{1-2\delta /\pi })}$. Introducing $\gamma =-\beta +\frac{\beta }{1-2\delta /\pi }=\frac{\beta \delta }{\pi /2-\delta }$, we re-write $\alpha =e^{-\beta (1+\lambda _{\text{min}})-\gamma }$. This allows us to specify $e^{-\gamma }$ instead of the Fourier convergence interval, i.e. to subordinate $\delta$ to the desired $\gamma$. This is done by fixing $\delta =\frac{\pi }{2}\frac{1}{1+\frac{\beta }{\gamma }}$. This, together with Eq. (18), leads to Eq. (2). $\square$

In fact, $4(\beta /\lambda +1)\ln (4/\epsilon ')$ is an upper-bound for the query complexity of P$_2$, as can be inferred from Eq. (18). In other words, the multiplicative factor implied in the ${\mathcal {O}}()$ in Eq. (2) notation is actually known to be equal to 4.

### Traditional master QITE algorithms

**Figure 7:**
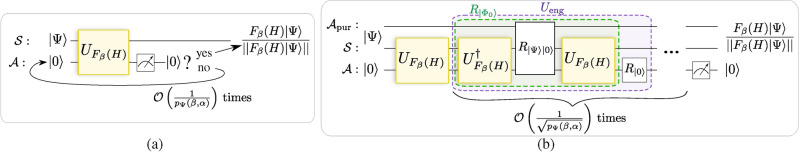
Probabilistic versus coherent master QITE algorithms. (**a**) The probabilistic approach repeatedly applies the unitary $U_{F_{\beta }(H)}$ generated by the primitive (on independent preparations of $|\Psi \rangle |0\rangle \in {\mathbb {H}}_\mathcal{S}\mathcal{A}$) until the post-selection is successful, i.e. the measurement on the ancillas returns $|0\rangle$ as outcome. This takes on average ${\mathcal {O}}\big (1/p_\Psi (\beta ,\alpha )\big )$ repetitions. No a priori knowledge of the input state $|\Psi \rangle$ is required (it can be fully generic, even mixed). (**b**) The coherent approach is based on quantum amplitude amplification. It operates only on pure input states. So, if the input state is mixed, an extra ancillary register ${\mathcal {A}}_{\text{pur}}$ of $|{\mathcal {A}}_{\text{pur}}|=|{\mathcal {S}}|=N$ qubits is required to purify it. This is the case in quantum Gibbs state sampling, where $|\Psi \rangle$ is a purification of the maximally mixed state on ${\mathbb {H}}_{\mathcal {S}}$. The primitive is repeated applied sequentially (on the same preparation of $|\Psi \rangle |0\rangle$), interleaved with reflection operators $R_{|\Phi _0\rangle }$ and $R_{|0\rangle }$ around the states $|\Phi _0\rangle =U_{F_\beta (H)}|\Psi \rangle |0\rangle \in {\mathbb {H}}_\mathcal{S}\mathcal{A}$ and $|0\rangle \in {\mathbb {H}}_{\mathcal {A}}$, respectively. In practice, this requires full a priori knowledge of $|\Psi \rangle$. The total number of repetitions of the primitive is ${\mathcal {O}}\big (1/\sqrt{p_\Psi (\beta ,\alpha )}\big )$, after which the desired output is obtained with probability close to 1. Hence, the coherent master algorithm displays a significantly lower overall query complexity than the probabilistic one. However, in return, the former requires much larger circuit depth than the latter.

The average number of times a QITE primitive is applied in probabilistic and coherent master QITE algorithms is $1/p_{\Psi }(\beta ,\varepsilon ^{\prime }, \alpha )$ and $1/\sqrt{p_{\Psi }(\beta ,\varepsilon ^{\prime }, \alpha )}$, respectively; see Fig. 7. Conveniently, for $\varepsilon \ll 1$, $1/p_{\Psi }(\beta ,\varepsilon ^{\prime }, \alpha )$ can be approximated by the more practical expression $1/p_{\Psi }(\beta ,\alpha )$ up to error ${\mathcal {O}}(\varepsilon )$. This follows from a Taylor expansion. The probabilistic algorithm applies the primitive on independent input state preparations (Fig. 7a) and stops at the moment that the first successful postselection on the ancillas happens. In contrast, the coherent one (Fig. 7b) leverages quantum amplitude amplification^35^, which we briefly discuss next.

The coherent amplification process is realized by repeatedly applying to $|\Phi _0\rangle =U_{F_\beta (H)}|\Psi \rangle |0\rangle$ the unitary operator

23 $$\begin{aligned} U_\text{eng}=R_{|\Phi _0\rangle }\,R_{|0\rangle }, \end{aligned}$$

where $R_{|\Phi _0\rangle }$ and $R_{|0\rangle }$ are respectively the reflection operators around $|\Phi _0\rangle \in {\mathbb {H}}_{\mathcal {S}}\otimes {\mathbb {H}}_{{\mathcal {A}}_{\text{pur}}}\otimes {\mathbb {H}}_{\mathcal {A}}$ and $|0\rangle \in {\mathbb {H}}_{\mathcal {A}}$. The former reflection can in turn be decomposed as $R_{|\Phi _0\rangle }=U_{F_\beta (H)}\,R_{|\Psi \rangle |0\rangle }\,U^{\dagger }_{F_\beta (H)}$, where $U^{\dagger }_{F_\beta (H)}$ is the inverse of the block-encoding $U_{F_\beta (H)}$ of the QITE propagator $F_\beta (H)$ and $R_{|\Psi \rangle |0\rangle }$ the reflection around $|\Psi \rangle |0\rangle$. Unitary $U_\text{eng}$ is sometimes referred to as the amplification engine. Importantly, $U_\text{eng}$ acts on the 2-dimensional subspace spanned by $|\Phi _0\rangle$ and $|\Phi _{\text{target}}\rangle \propto | 0 \rangle \langle 0 |\,|\Phi _0\rangle$ as an SU(2) rotation. For $k\in {\mathbb {N}}$, it gives

24 $$\begin{aligned} U_\text{eng}^k\,|\Phi _0\rangle= & {} \sin [(2k+1)\theta ]\,|\Phi _{\text{target}}\rangle \nonumber \\{} & {} +\cos [(2k+1)\theta ]\,|\Phi _{\perp }\rangle , \end{aligned}$$

where $|\Phi _{\perp }\rangle \propto | 1 \rangle \langle 1 |\,|\Phi _0\rangle$ and $\sin (\theta )=\alpha \Vert F_\beta (H)|\Psi \rangle \Vert$. Hence, taking $k=k_{\text{opt}}={\mathcal {O}} (1/\theta )$ yields $\sin [(2k+1)\theta ]\approx 1$ and therefore probability close to 1 for desired output. For $\theta \ll 1$, this entails $k={\mathcal {O}} (1/\alpha \Vert F_\beta (H)|\Psi \rangle \Vert )={\mathcal {O}} (1/\sqrt{p_\Psi (\beta ,\alpha )})$ repetitions of the primitive, as in Eq. (4).

Finally, since $\alpha ^2\Vert F_\beta (H)|\Psi \rangle \Vert$ is in general unknown, one has no a priori knowledge of $k_{\text{opt}}$. However, fortunately, this can be accounted for with successive attempts with *k* randomly chosen within a range of values that grows exponentially with the number of attempts (see^35^, Theorem 3]). Remarkably, the resulting average number of applications of the primitive remains within ${\mathcal {O}} (1/\sqrt{p_\Psi (\beta ,\alpha )})$.

### Minimum query complexity of QITE primitives based on block-encoding oracles

Our proof strategy for Theorem 3 is analogous to that of the no-fast-forwarding theorem for real-time evolutions^38–40^. That is, it is based on a reduction to QITE of the task of determining the parity $\text{par}({\varvec{x}})=x_{0}\oplus x_{1}\oplus \cdots \oplus x_{N-1}$, with $\oplus$ the bit-wise sum, of an unknown *N*-bit string ${\varvec{x}}=x_{0}\,x_{1}\cdots x_{N-1}$ from a parity oracle $U_{{\varvec{x}}}$ for ${\varvec{x}}$; together with known fundamental complexity bounds for the latter task^72,73^. More precisely, our proof relies on three facts: *i*) No algorithm can find $\text{par}({\varvec{x}})$ from $U_{{\varvec{x}}}$ with fewer than a known number of queries to it^72,73^; *ii*) a QITE primitive querying an oracle for an appropriate Hamiltonian $H_{{\varvec{x}}}$ gives an algorithm to find $\text{par}({\varvec{x}})$; and *iii*) a block-encoding oracle for $H_{{\varvec{x}}}$ can be synthesized from one call to $U_{{\varvec{x}}}$. The three facts are established in the following lemmas.

The first lemma, proven in^72^, lower-bounds the complexity of any quantum circuit able to obtain par$({\varvec{x}})$ from queries to $U_{{\varvec{x}}}$. For our purposes, it can be stated as follows.

#### Lemma 11

Let *C* be a quantum circuit composed of ${\varvec{x}}$-independent gates and *q* times the ${\varvec{x}}$-dependent unitary

25 $$\begin{aligned} U_{{\varvec{x}}} =\sum _{j=0}^{N} |j\rangle \langle j| \otimes X^{x_j} \, \end{aligned}$$

with $\{|j\rangle \}_{j\in [N+1]}$ an orthogonal basis, such that, acting on an ${\varvec{x}}$-independent input state and upon measurement on an ${\varvec{x}}$-independent basis, outputs $\text{par}({\varvec{x}})$ with probability greater than 1/2 for all ${\varvec{x}}\in \{0,1\}^N$. Then, $q\ge \lceil N/2\rceil$.

The second lemma, proven in^45^, Sec. XVII, reduces parity finding to QITE and is the key technical contribution of this section.

#### Lemma 12

Let $\beta >0$, $\varepsilon '>0$, $\alpha \in (0,1]$, and ${\varvec{x}}$ an *N*-bit string such that

26 $$\begin{aligned} \left| \frac{1-e^{-\frac{\beta }{2N}}}{2} \right| ^N>\frac{2\,\varepsilon '}{\alpha } . \end{aligned}$$

Then, there exists $H_{{\varvec{x}}}$, with $\Vert H_{{\varvec{x}}}\Vert \le 1$, such that a $(\beta ,\varepsilon ',\alpha )$-QITE-primitive with calls to a block-encoding oracle for $H_{{\varvec{x}}}$, acting on an ${\varvec{x}}$-independent input state and upon measurement on an ${\varvec{x}}$-independent basis, outputs $\text{par}({\varvec{x}})$ with probability greater than 1/2 for all ${\varvec{x}}\in \{0,1\}^N$.

Finally, the missing link between Lemmas 11 and 12 is a sub-routine to query $H_{{\varvec{x}}}$ given queries to $U_{{\varvec{x}}}$ in Eq. (25). This is provided by the following lemma, proven in^45^, Sec. XVIII.

#### Lemma 13

A block-encoding oracle for $H_{{\varvec{x}}}$ can be generated from a single query to $U_{{\varvec{x}}}$ and ${\mathcal {O}}(N)$ ${\varvec{x}}$-independent gates, for all ${\varvec{x}}\in \{0,1\}^N$. (See^45^ Fig. S10 for circuit.).

#### Proof of Theorem 3

With these three Lemmas, the proof of Theorem 3 is straightforward. First, note that the left-hand side of Eq. (26) decreases monotonically in *N*. Hence, for any fixed $(\beta ,\varepsilon ',\alpha )$, the largest $N\in {\mathbb {N}}$ that satisfies Eq. (26) is $N=\lfloor 2{\tilde{q}}\rfloor$, with ${\tilde{q}}\in {\mathbb {R}}$ defined by Eq. (3). This, together with Lemmas 12 and 13, implies that any $(\beta ,\varepsilon ',\alpha )$-QITE-primitive synthesized from queries to the parity oracle $U_{{\varvec{x}}}$ provides a quantum circuit to determine the parity of any string ${\varvec{x}}$ of length $\lfloor 2{\tilde{q}}\rfloor$. Then, by virtue of Lemma 11, the query complexity $q_{\min }(\beta ,\varepsilon ',\alpha )\in {\mathbb {N}}$ of the primitive cannot be smaller than $q\ge \lceil N/2\rceil =\Big \lceil \frac{\lfloor 2{{\tilde{q}}}\rfloor }{2}\Big \rceil$. Note that this number is the nearest integer to ${\tilde{q}}$. Ergo, $q_{\min }(\beta ,\varepsilon ',\alpha )\ge {\tilde{q}}$. $\square$

Finally, a comment on why Theorem 3 does not hold for QITE primitives based on real-time evolution (RTE) oracles is useful at this point. The reason is that, by virtue of the RTE no-fast-forwarding theorem^38–40^, a single call to an RTE oracle suffices to find the parity with probability greater than 1/2. Hence, it is the query complexity RTE oracles themselves what is lower-bounded by the parity considerations above, but not that of RTE-based QITE primitives. It is an open question whether similar bounds can be obtained for RTE-based QITE primitives by other arguments.

### Supplementary Information

Supplementary Information.

## Data Availability

The datasets generated and/or analyzed during the current study are available from the corresponding author on reasonable request. The programming codes utilized are available at https://doi.org/10.5281/zenodo.5595705.
