# Supplementary material for: Fragmented imaginary-time evolution for early-stage quantum signal processors
Source: Sci Rep. 2023 Oct 25;13:18258. doi: 10.1038/s41598-023-45540-2 (PMC10600201; doi:10.1038/s41598-023-45540-2)
Supplement: Supplementary file 1 — Supplementary Information. [file 41598_2023_45540_MOESM1_ESM.pdf]

Supplementary information for "Fragmented imaginary-time evolution for early-stage quantum signal processors"

Thais L. Silva,[1,2] Márcio M. Taddei,[2,3] Stefano Carrazza,[4,1] and Leandro Aolita[1,2]

[1]*Quantum Research Centre, Technology Innovation Institute, Abu Dhabi, UAE*
[2]*Federal University of Rio de Janeiro, Caixa Postal 68528, Rio de Janeiro, RJ 21941-972, Brazil*
[3]*ICFO - Institut de Ciencies Fotòniques, The Barcelona Institute of Science and Technology, 08860, Castelldefels, Barcelona, Spain*
[4]*TIF Lab, Dipartimento di Fisica, Università degli Studi di Milano and INFN Sezione di Milano, Milan, Italy*

I. OPERATOR-FUNCTION DESIGN FROM IMPERFECT ORACLES

Both algorithms for operator-function design we present assume access to ideal oracles as given by Def. 2 and Def. 3. This is not the case in an experimental scenario where only approximate oracles are available. This leads to a total error in the algorithm that is proportional to the number of approximate oracle calls. The following lemma deals with that error for generic operator functions.

Lemma 1. *(Primitives from imperfect oracles) Let a circuit with q queries to an oracle O for H generate an (ε, α)-block-encoding $V_{f(H)}$ of $f(H)$, for arbitrary f. If O is substituted by $\tilde{O}$, with $\|O - \tilde{O}\| \leq \varepsilon_O$, the circuit generates an $(\tilde{\varepsilon}, \alpha)$-block-encoding $\tilde{V}_{f(H)}$ of $f(H)$ with $\tilde{\varepsilon} \leq \varepsilon + q\,\varepsilon_O$.*

Proof. For $q\,\varepsilon_O \ll 1$, straightforward matrix multiplication shows that the total error is $||V_{f(H)} - \tilde{V}_{f(H)}|| = \mathcal{O}(q\,\varepsilon_O)$. This implies $||f(H) - \langle 0|\tilde{V}_{f(H)}|0\rangle|| \leq \varepsilon_P + q\,\varepsilon_O$ by virtue of the triangle inequality. This concludes the proof. □

As an exemplary application of Lemma 1, we calculate the gate complexity $g_{\tilde{O}_2}$ required to implement an approximate real-time evolution oracle $\tilde{O}_2$ for QITE primitive P_2 by Trotter-Suzuki-like simulation methods. To attain a total error ε', we choose $\varepsilon = \varepsilon'/2$ and $\varepsilon_O = \varepsilon'/(2q)$. For real time t, the gate complexity of state-of-the-art algorithms [1, 2] based on first-order product formulae is $\mathcal{O}(t^2/\varepsilon_O)$. Substituting for ε_O, and then for t and q with the real time and query complexity given in Theorem 2, we obtain the oracle gate complexity $g_{\tilde{O}_2} = \mathcal{O}\left(\frac{\pi^2\beta}{\gamma}\frac{\ln(8/\varepsilon')}{2\,\varepsilon'(1+\gamma/\beta)}\right)$. This scaling is exponentially worse in $1/\varepsilon'$ than what we would get if we simulated O_2 with more sophisticated real-time evolution algorithms [3–7]. However, those algorithms assume more powerful oracles, which require significantly more ancillary qubits. The fact that P_2 can be synthesised with only 1 ancillary qubit throughout and with no qubitization makes it appealing for near-term implementations on intermediate-sized quantum hardware.

II. POST-SELECTION PROBABILITY PROPAGATION ONTO OUTPUT STATE ERROR

Since $\alpha\,F_\beta(H)$ is not unitary, the (operator-norm) error ε' in its approximation by an (α, ε')-block-encoding gets amplified at the post-selection due to state renormalization. The following allows us to control the error in the output state.

Lemma 2. *(Error propagation from block encoding to output state) Let $p_\Psi(\beta, \alpha) \leq 1$ be the post-selection probability of an (α, ε')-block-encoding $U_{F_\beta(H)}$ of $F_\beta(H)$ on an input state $|\Psi\rangle \in \mathbb{H}_\mathcal{S}$. Then, if $\varepsilon' \leq \varepsilon\sqrt{p_\Psi(\beta,\alpha)}/2$ and $\varepsilon \ll 1$, the output-state trace-distance error is $\mathcal{O}(\varepsilon)$.*

Proof. By definition, $\|\langle 0|\; U_{F_\beta(H)}|0\rangle - \alpha\,F_\beta(H)\| \leq \varepsilon'$. Then, for some $|\Xi\rangle \in \mathbb{H}_S$, with $\||\Xi\rangle\| \leq \varepsilon'$, it is

$$\langle 0|\; U_{F_\beta(H)}|0\rangle\; |\Psi\rangle = \alpha\,F_\beta(H)|\Psi\rangle + |\Xi\rangle \tag{S1a}$$

and, so, for some $\epsilon \in \mathbb{R}$, with $|\epsilon| \leq \||\Xi\rangle\| \leq \varepsilon'$, we get

$$\|\langle 0|\; U_{F_\beta(H)}|0\rangle\; |\Psi\rangle\| = \alpha\,\|F_\beta(H)|\Psi\rangle\| + \epsilon. \tag{S1b}$$

Next, we Taylor-expand the output state in terms of ε. To that end, note first that

$$\begin{aligned}\frac{\langle 0|\; U_{F_\beta(H)}|0\rangle\; |\Psi\rangle}{\|\langle 0|\; U_{F_\beta(H)}|0\rangle\; |\Psi\rangle\|} &= \frac{\alpha\,F_\beta(H)|\Psi\rangle + |\Xi\rangle}{\alpha\,\|F_\beta(H)|\Psi\rangle\| + \epsilon} \\ &= \frac{F_\beta(H)|\Psi\rangle}{\|F_\beta(H)|\Psi\rangle\|}\left(1 - \frac{\epsilon}{\alpha\,\|F_\beta(H)|\Psi\rangle\|}\right) \\ &+ \frac{|\Xi\rangle}{\alpha\,\|F_\beta(H)|\Psi\rangle\|} + \mathcal{O}\left(\frac{\varepsilon'\,|\Xi\rangle}{\alpha^2\,\|F_\beta(H)|\Psi\rangle\|^2}\right)\;.\end{aligned} \tag{S2}$$

So, the output state-vector error is upper-bounded as

$$\left\| \frac{\langle 0|\, U_{F_\beta(H)}|0\rangle\, |\Psi\rangle}{\|\langle 0|\, U_{F_\beta(H)}|0\rangle\, |\Psi\rangle\|} - \frac{F_\beta(H)|\Psi\rangle}{\|F_\beta(H)|\Psi\rangle\|} \right\| \leq \frac{2\,\varepsilon'}{\alpha\, \|F_\beta(H)|\Psi\rangle\|} + \mathcal{O}\left(\frac{\varepsilon'^2}{\alpha^2\, \|F_\beta(H)|\Psi\rangle\|^2}\right). \tag{S3}$$

Hence, if $\varepsilon' \leq \varepsilon\, \alpha\, \|F_\beta(H)|\Psi\rangle\|/2 = \varepsilon\, \sqrt{p_\Psi(\beta,\alpha)}/2$, the error in l_2-norm distance of the output state vector is at most $\varepsilon + \mathcal{O}(\varepsilon^2)$, which equals $\mathcal{O}(\varepsilon)$ for $\varepsilon \ll 1$.

With the l_2-norm distance between the two state vectors, we can upper-bound the trace distance between their corresponding rank-1 density operators using well-known inequalities. First, note that the l_2-norm distance between two arbitrary normalised vectors $|\phi\rangle$ and $|\psi\rangle$ can be expressed as

$$\||\phi\rangle - |\psi\rangle\| = \sqrt{2}\sqrt{1 - \mathrm{Re}\langle\psi|\phi\rangle}. \tag{S4}$$

Second, recall that the trace distance

$$\||\phi\rangle\langle\phi| - |\psi\rangle\langle\psi|\|_{\mathrm{tr}} := \frac{1}{2}\mathrm{tr}\sqrt{(|\phi\rangle\langle\phi| - |\psi\rangle\langle\psi|)^2}\,. \tag{S5}$$

between rank-1 density operators $|\phi\rangle\langle\phi|$ and $|\psi\rangle\langle\psi|$ can be written in terms of the overlap as

$$\||\phi\rangle\langle\phi| - |\psi\rangle\langle\psi|\|_{\mathrm{tr}} = \sqrt{1 - |\langle\psi|\phi\rangle|^2}\,. \tag{S6}$$

Then, note that, for all $|\phi\rangle$ and $|\psi\rangle$, the RHS of Eq. (S4) upper-bounds the RHS of Eq. (S6). Hence, $\||\phi\rangle\langle\phi| - |\psi\rangle\langle\psi|\|_{\mathrm{tr}} \leq \||\phi\rangle - |\psi\rangle\|$. This, together with Eq. (S3) gives the promised trace-norm error of the output state. □

III. OPTIMAL SUB-NORMALISATION FOR QITE PRIMITIVE 2

In this appendix, we prove that, for $\varepsilon' \ll 1$,

$$\gamma_\kappa(\beta) := \frac{\beta}{2}\left(\sqrt{1 + \frac{2}{\mu_\kappa\, \beta}} - 1\right). \tag{S7}$$

gives approximately the optimal value for the subnormalization of P_2, such that the overall query complexity given by Eq. (4) is minimized. Notice that, by diminishing the value of γ we increase the success probability of P_2, decresing the number of times it needs to be realized. At the same time, it leads to an increment on the query complexity of P_2 given by Eq. (2). Therefore, the optimal subnormalization is a tradeoff between these two contributions.

From Eqs. (4) and (2), given β, ε, and the master algorithm type κ, we get that the optimal γ minimizes

$$Q_\kappa(\beta,\varepsilon,\gamma) = \frac{e^{2\mu_\kappa\gamma}}{\|F_\beta(H)|\Psi\rangle\|^{2\mu_\kappa}}\left(\frac{\beta}{\gamma} + 1\right)\left[\ln\left(\frac{8}{\|F_\beta(H)|\Psi\rangle\|\varepsilon}\right) + g\gamma\right], \tag{S8}$$

with $g = 1$ introduced for convenience. Here we have used $p_\Psi(\beta,\gamma) = e^{-2\gamma}\|F_\beta(H)|\Psi\rangle\|$ and $\varepsilon' = p_\Psi(\beta,\gamma)\varepsilon/2$.

Instead, let $\gamma = \gamma_\kappa(\beta)$ be such that $Q_\kappa(\beta,\varepsilon,\gamma)|_{g=0}$ is minimized. By solving $\frac{\partial}{\partial\gamma}Q_\kappa(\beta,\varepsilon,\gamma)\big|_{g=0} = 0$ we obtain $\gamma_\kappa(\beta)$ given by Eq. (S7). In order to prove that that expression is a good approximation for the actual optimal value $\gamma_\kappa^{(\mathrm{opt})}(\beta)$, first notice that, with $g = 1$, it is easy to verify that $Q_\kappa(\beta,\varepsilon,\gamma)|_{g=1} > Q_\kappa(\beta,\varepsilon,\gamma_\kappa(\beta))|_{g=1}$ if $\gamma > \gamma_\kappa(\beta)$. Therefore, $\gamma_\kappa^{(\mathrm{opt})}(\beta) < \gamma_\kappa(\beta)$. Defining $\Delta Q := Q_\kappa(\beta,\varepsilon,\gamma_\kappa^{(\mathrm{opt})}(\beta))|_{g=1} - Q_\kappa(\beta,\varepsilon,\gamma_\kappa(\beta))|_{g=1}$, a Taylor expansion of $Q_\kappa(\beta,\varepsilon,\gamma)|_{g=1}$ at $\gamma = \gamma_\kappa(\beta)$ shows that, up to first order in $\Delta\gamma = \gamma_\kappa^{(\mathrm{opt})}(\beta) - \gamma_\kappa(\beta)$, we have

$$\frac{\Delta Q}{Q_\kappa(\beta,\varepsilon,\gamma_\kappa(\beta))|_{g=1}} \simeq \frac{\Delta\gamma}{\ln\left(\frac{8e^{2\gamma_\kappa(\beta)}}{\|F_\beta(H)|\Psi\rangle\|\varepsilon}\right)}, \tag{S9}$$

which is much smaller than 1 provided that $\varepsilon' \ll 1$, since $\gamma_\kappa(\beta) < 1$ and, therefore, $\Delta\gamma < 1$.

IV. PROOF OF THEOREM 4

Let us first prove a convenient auxiliary lemma. Ideally, the input and output states of the l-th fragment of should be $|\Psi_{l-1}\rangle = \frac{F_{\beta_{l-1}}(H)|\Psi\rangle}{\|F_{\beta_{l-1}}(H)|\Psi\rangle\|}$ and $|\Psi_l\rangle = \frac{F_{\Delta\beta_l}(H)|\Psi_{l-1}\rangle}{\|F_{\Delta\beta_l}(H)|\Psi_{l-1}\rangle\|} = \frac{F_{\beta_l}(H)|\Psi\rangle}{\|F_{\beta_l}(H)|\Psi\rangle\|}$, respectively. However, the actual operator that each $P_{\Delta\beta_l,\varepsilon'_l,\alpha_l}$ perfectly block-encodes is

$$\alpha_l\tilde{F}_{\Delta\beta_l}(H) := \langle 0|\, U_{F_{\Delta\beta_l}(H)}|0\rangle = \alpha\, F_{\Delta\beta_l}(H) + E_l, \tag{S10}$$

for some E_l on $\mathbb{H}_{\mathcal{S}}$ with $||E_l|| =: \varepsilon'_l$. Hence, in analogy to Eq. (S1b), the actual input and output states are $|\tilde{\Psi}_{l-1}\rangle = |\Psi_{l-1}\rangle + |\Xi_{l-1}\rangle$ and $|\tilde{\Psi}_l\rangle = |\Psi_l\rangle + |\Xi_l\rangle$, for some “error

states" $|\Xi_{l-1}\rangle$ and $|\Xi_l\rangle$, respectively. (Note that $|\Xi_0\rangle = 0$.) The following lemma controls the output-state error in terms of the errors in the input state and block encoding.

Lemma 3. *(Error propagation from input state and block encoding to output state) For all $l \in [r]$, let $\varepsilon_{l-1} := \||\Xi_{l-1}\rangle\|$ and ε'_l be the input-state and block-encoding errors, respectively; and ε_l the tolerated output-state error. If $\varepsilon_{l-1} + \varepsilon'_l \le \varepsilon_l \|\alpha_l F_{\Delta\beta_l}(H)|\Psi_{l-1}\rangle\|/2$ and $\varepsilon_l \ll 1$, then $\||\Xi_l\rangle\| = \mathcal{O}(\varepsilon_l)$.*

Proof. Similar to the proof of Lemma 2 but where also the input state is approximate. □

Note that the proof of Lemma 2 (and therefore also that of Lemma 3) makes no use of the specific form of F_β (or $F_{\Delta\beta_l}$). Consequently, both lemmas hold for (α, ε')-block-encodings of any operator function, not just the QITE propagator. We are now in a good position to prove Theorem 4.

Proof of Theorem 4. We begin by proving soundness. The proof consists of showing that Eqs. (5) imply that Lemma 3 holds for all $l \in [r]$ and gives $\varepsilon_r := \||\Xi_r\rangle\| = \mathcal{O}(\varepsilon)$. To see this, apply the lemma from $l = 1$ till $l = r$, with $\varepsilon_0 = 0$, and iteratively use the property

$$\begin{aligned} p_{\Psi_{l-1}}(\Delta\beta_l) &= \|F_{\Delta\beta_l}(H)|\Psi_{l-1}\rangle\| \\ &= \frac{\|F_{\beta_l}(H)|\Psi\rangle\|}{\|F_{\beta_{l-1}}(H)|\Psi\rangle\|} \\ &= \frac{p_\Psi(\beta_l)}{p_\Psi(\beta_{l-1})}. \end{aligned} \quad \text{(S11)}$$

We next prove complexity. To this end, we must show the validity of Eq. (6). By Def. 5, the overall query complexity of Algorithm 1 is the sum over the query complexities of each primitive applied. Each primitive $P_{\Delta\beta_l,\varepsilon'_l,\alpha_l}$ has complexity $q(\Delta\beta_l, \varepsilon'_l, \alpha_l)$. Hence, we must show that the average number of times n_l that each $P_{\Delta\beta_l,\varepsilon'_l,\alpha_l}$ is run is given by $\frac{p_\Psi(\beta_{l-1})}{p_\Psi(\beta)\prod_{k=l}^r \alpha_k^2}$. To see this, note that, by definition, it is $n_r := \left({\alpha_r}^2 p_{\Psi_{r-1}}(\Delta\beta_r)\right)^{-1}$, $n_{r-1} := n_r \times \left({\alpha_{r-1}}^2 p_{\Psi_{r-2}}(\Delta\beta_{r-1})\right)^{-1}$, ..., and $n_1 := n_2 \times \left({\alpha_1}^2 p_\Psi(\Delta\beta_1)\right)^{-1}$. Then, use the latter together with Eq. (S11) to get $n_l = \frac{p_\Psi(\beta_{l-1})}{p_\Psi(\beta)\prod_{k=l}^r \alpha_k^2}$ for all $l \in [r]$. □

Interestingly, we note that the only specific detail about F_β that the proof of Theorem 4 uses is the fact that $F_\beta(H) = \prod_{l=1}^r F_{\Delta\beta_l}(H)$ (this is required for Eq. (S11) to hold). Consequently, Theorem 4 applies not only to fragmented QITE but actually also to the fragmentation of any operator function in terms of suitable factors.

V. VALIDITY OF THE QUERY COMPLEXITY BOUNDS OF QITE PRIMITIVES FOR LOW BETA

As discussed in Secs. 2.3 and 2.4, the first steps of fragmented QITE involve small inverse temperatures, often with $\Delta\beta_1 < 1$. However, Eqs. (1) and (2) are in principle only asymptotic upper bounds for the actual query complexities. Hence, it is licit to question how valid they are to access the complexity of the first steps of Alg. 1. In this appendix, we discuss the validity and tightness for all $\beta > 0$ of

$$\tilde{q}_1(\beta, \varepsilon') := 2\left(\frac{e\beta}{2} + \frac{\ln(1/\varepsilon')}{\ln\left(e + 2\ln(1/\varepsilon')/e\beta\right)}\right) \quad \text{(S12)}$$

and

$$\tilde{q}_2(\beta, \varepsilon') := 4\left(\beta/\gamma + 1\right)\ln(4/\varepsilon') \quad \text{(S13)}$$

(without multiplicative factors implied by the big-$\mathcal{O}$ notation) as exact expressions for the query complexities of primitives 1 and 2, respectively. These formulas are the ones used in our numeric experiments. First of all, notice that although these expressions are continuous functions, the actual query complexities used by Algs. 2 and 3 take only even integer values. Thus, we take the value of the query for each round of fragmentation as $2\lceil\tilde{q}_k(\beta, \varepsilon')/2\rceil$, $k = 1, 2$. In particular, for any value of β such that $0 < \tilde{q}_1(\beta, \varepsilon') \le 2$ or $0 < \tilde{q}_2(\beta, \varepsilon') \le 2$ at least two queries to the corresponding oracle is used.

For P_2 there is actually no concern because the reasoning of Sec. 4.2.3 that led to Eq. (2) is valid for any beta. This is because $q_2 = 2\lceil\tilde{q}_2(\beta, \varepsilon')/2\rceil$ is the exact expression furnished by Lemma 37 in Ref. [8], used to prove Lemma 10, for $\alpha F_\beta(\lambda)$. Therefore, although this formula is not strictly tight, because it comes from successive approximations [8], it is exact and valid for any $\beta > 0$ in the sense that making exactly $2\lceil\tilde{q}_2(\beta, \varepsilon')/2\rceil$ queries to the oracle ensures that the error is below the tolerated. Consequently, the value $2\lceil\tilde{q}_2(\beta, \varepsilon')/2\rceil$ is an upper bound for the necessary number of queries without any multiplicative factor that could be implied by the big-$\mathcal{O}$ notation.

P_1, on the other hand, requires a further analysis since, according to Eq. (1), $q_1(\beta, \varepsilon')$ is known to be equal to $\tilde{q}_1(\beta, \varepsilon')$ only in big-$\mathcal{O}$ notation and, particularly, for large β. Next, we numerically show that $\tilde{q}_1(\beta, \varepsilon')$ is, in fact, an

over-estimation of the actual $q_1(\beta,\varepsilon')$ needed to guarantee error ε'.

We notice that the first inequality in Eq. (16) gives a tighter upper bound for the query complexity. Therefore, to attain a target error ε', it is enough that $q_1 = 2\left\lceil \tilde{q}_1(\beta,\varepsilon')/2\right\rceil$ queries yields to a truncation error which satisfies

$$\varepsilon_{\text{tr}} \leq \varepsilon_{\text{up}}^{(q_1)}(\beta) = \frac{\beta^{q_1/2+1}}{2^{q_1/2}(q_1/2+1)!} < \varepsilon'. \tag{S14}$$

As we explain next, Fig. S1 shows that it is, in fact, what happens. In Fig. S1a) we show the interval of β's for which the value $\tilde{q}_1(\beta,\varepsilon')$ is between $q_1 = 18$ and $q_1 = 20$. For any β inside this interval, the number of queries made in P_1 will be $q_1 = 20$. However, in Fig. S1b) when we look at the upper bound for the truncation error with 20 queries, $\varepsilon_{\text{up}}^{(q_1=20)}$, in the same interval, we see that it is much smaller than the tolerated error ε'. Consequently, the truncation error ε_{tr}, which is the actual error of P_1, is also below ε'. This is also observed in Figs. S1c) and S1d), where the interval of β's for which the minimum number of $q_1 = 2$ queries is made and the corresponding $\varepsilon_{\text{tr}}^{(q_1=2)}$ in that interval are shown, respectively. We tested for other values of ε' ranging from 10^{-1} to 10^{-10} and the same behavior is observed. This shows that $\tilde{q}_1(\beta,\varepsilon')$ is valid as the query complexity of P_1 even for small values of β. Moreover, it actually overestimates the minimum query complexity for a given target error ε'.

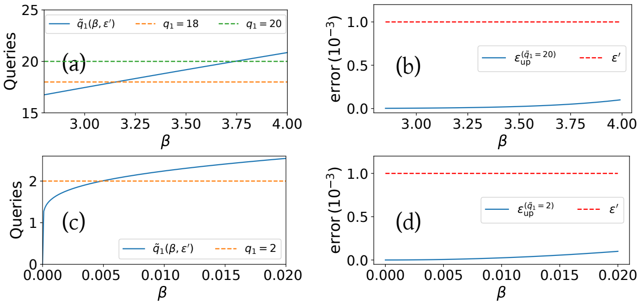

Figure S1. (a) Zoom in on the query complexity function $\tilde{q}_1(\beta,\varepsilon')$ for fixed error $\varepsilon' = 10^{-3}$. For any value of inverse temperature β for which $18 \leq \tilde{q}_1(\beta,\varepsilon') \leq 20$, $q_1 = 20$ queries are made in P_1. (b) the corresponding tighter error bound $\varepsilon_{up}^{(q_1=20)}(\beta)$ from Eq. (S14) for $q_1 = 20$ queries in the same interval of β, which is way below the tolerated error ε'. Parts (c) and (d) show the same thing for the minimum number of queries $q_1 = 2$, showing that $\varepsilon_{up}^{(q_1=2)}(\beta)$ is much smaller than ε' for any β such that $\tilde{q}_1(\beta,\varepsilon') \leq 2$. Therefore (see text), $2\lceil\tilde{q}_1(\beta,\varepsilon')/2\rceil$ is a valid upperbound for the number of queries of P_1 even when β is small.

VI. FRAGMENTED QITE OUTPERFORMS COHERENT QITE

Here we analytically show, for primitive P_1, that there exists an inverse temperature β_c above which fragmented QITE outperforms coherent QITE (based on quantum amplitude amplification) in terms of overall query complexity. Before the proof, it is useful to recall that the success probability (of any QITE primitive) is given by $p_\Psi(\beta) = \sum_\lambda |\langle\lambda|\Psi\rangle|^2 e^{-2\beta(\lambda-\lambda_{\min})}$. Note also that $p_\Psi(\beta) \geq |\langle\lambda_{\min}|\Psi\rangle|^2 = o^2$. Hence,

$$\sqrt{p_\Psi(\beta)} \geq o, \tag{S15}$$

for all $\beta \geq 0$. Moreover, $p_\Psi : [0,\infty) \to [o^2, 1]$ is a monotonically decreasing function. Therefore, it has an inverse function, which we denote as p_Ψ^{-1}.

Proof of Theorem 5. We assume here that Primitive 1 is run using a number of queries equals to the upper-bound that guarantees a given error. A multiplicative factor would evenly affect the complexities of all master algorithms. For simplicity, we omit here the multiplicative factor (shown to be equal to 8 in the proof of Theorem 1) and take the complexity of P_1 directly as $q_1(\beta,\varepsilon') = \frac{e\beta}{2} + \frac{\ln(1/\varepsilon')}{\ln(e+2\ln(1/\varepsilon')/e\beta)}$. This is also justified in the tightness analysis of App. V.

We need to show that there exists a schedule $S_2 = \{\Delta\beta_1, \Delta\beta_2\}$ such that the average overall query complexities satisfy $Q_{S_2}(\beta,\varepsilon) \leq Q_{\text{coh}}(\beta,\varepsilon)$. According to Eqs. (4) and (6), this holds if

$$\sum_{l=1}^{2} \frac{p_\Psi(\beta_{l-1})}{\left(p_\Psi(\beta)\right)^{1/2}}\, q_1(\Delta\beta_l, \varepsilon'_l) \leq q_1(\beta,\varepsilon'), \tag{S16}$$

where ε'_l is given by Eq. (5). It is useful to introduce the upper bound $\tilde{q}_1(\beta,\varepsilon') := \frac{e\beta}{2} + \ln\left(\frac{1}{\varepsilon'}\right)$. (Note that $\tilde{q}_1(\beta,\varepsilon') > q_1(\beta,\varepsilon')$ for all $\beta > 0$.) Then, Eq. (S16) holds if

$$\sum_{l=1}^{2} \frac{p_\Psi(\beta_{l-1})}{\left(p_\Psi(\beta)\right)^{1/2}}\, \tilde{q}_1(\Delta\beta_l, \varepsilon'_l) \leq q_1(\beta,\varepsilon'). \tag{S17}$$

Next, we break this inequality into two inequalities, one for $\Delta\beta_1$ and another for $\Delta\beta_2$. Then, we show that, under the theorem's assumptions, both inequalities can be satisfied.

More precisely, Eq. (S17) is satisfied if the following two inequalities are simultaneously satisfied:

$$\frac{p_\Psi(\beta_{l-1})}{(p_\Psi(\beta))^{1/2}}\,\tilde{q}_1(\Delta\beta_l,\varepsilon'_l) \leq \frac{1}{2}q_1(\beta,\varepsilon'),\;\; \text{for } l=1,2\;; \tag{S18}$$

and we construct an S_2 that fulfills this. First, substituting for $\tilde{q}_1$ and q_1, we find that each fragment must satisfy

$$\begin{aligned}\Delta\beta_l \;&\leq\; \frac{1}{2}\frac{\sqrt{p_\Psi(\beta)}}{p_\Psi(\beta_{l-1})}\left(\beta+\frac{2}{e}\frac{\ln\left(\frac{1}{\varepsilon'}\right)}{\ln\left(e+2\ln(1/\varepsilon')/e\beta\right)}\right)\\ &-\;\frac{2}{e}\ln\left(\frac{1}{\varepsilon'_l}\right),\;\;\text{for } l=1,2.\end{aligned} \tag{S19}$$

For consistency, the right-hand side of Eq. (S19) should be positive for each l, the most critical case being that of $l=1$, when the first term is less positive while the second term is more negative as compared with the case $l=2$. So it suffices to enforce positivity of the right-hand side of Eq. (S19) for $\beta \geq \beta_c$ only for $l=1$. One can directly see that this is already satisfied by plugging the expression for β_c into Eq. (S19) and using Eq. (S15).

Because $\beta = \Delta\beta_1 + \Delta\beta_2$, we are free to choose the size of only one fragment, say $\Delta\beta_1 \equiv \beta_1$. Eq. (S19) imposes an upper on β_1 for $l=1$ and a lower bound for $l=2$. We first find a β_1 that satisfies the lower bound and then show that, for $\beta \geq \beta_c$, the upper bound is automatically satisfied. Using Eq. (5) and $\Delta\beta_2 = \beta - \beta_1$, we re-write Eq. (S19) for $l=2$ as

$$\begin{aligned}0 \;&\leq\; \beta_1 + \left[\frac{\sqrt{p_\Psi(\beta)}}{2\,p_\Psi(\beta_1)}-1\right]\beta\\ &+\;\frac{2}{e}\left[\frac{\sqrt{p_\Psi(\beta)}}{2\,p_\Psi(\beta_1)\ln\left[e+2\ln(1/\varepsilon')/e\beta\right]}-1\right]\ln\left(\frac{1}{\varepsilon'}\right)\\ &+\;\frac{2}{e}\ln\left(\frac{1}{2\sqrt{p_\Psi(\beta_1)}}\right).\end{aligned} \tag{S20}$$

Sufficient to satisfy this inequality is that each term on the right-hand side is positive. Since $\ln\left[e+2\ln(1/\varepsilon')/e\beta\right] > 1$, the third term is always smaller than the second one. Therefore, requiring that

$$\sqrt{p_\Psi(\beta)} \geq 2\,p_\Psi(\beta_1)\ln\left[e+2\ln(1/\varepsilon')/e\beta\right] \tag{S21}$$

ensures that both the second and the third terms are positive. This condition is in turn satisfied if $p_\Psi(\beta_1) \leq \frac{\sqrt{p_\Psi(\beta)}}{2\,\ln\left[e+2\ln(1/\varepsilon')/e\beta\right]}$, which is equivalent to demanding

$$\beta_1 \geq p_\Psi^{-1}\left(\sqrt{p_\Psi(\beta)}/2\ln\left[e+2\ln(1/\varepsilon')/e\beta\right]\right). \tag{S22}$$

Notice that Eq. (S21) also ensures that $\sqrt{p_\Psi(\beta_1)} \leq 1/2$ for $\beta \geq \beta_c$, due to $p_\Psi(\beta) \leq 1/4$ by theorem assumption. This makes the last term in Eq. (S20) also positive. Now, because of Eq. (S15), sufficient to satisfy Eq. (S22) is to demand that

$$\beta_1 \geq p_\Psi^{-1}\left(\frac{o}{2}\frac{1}{\ln[e+2\ln(2/o\,\varepsilon)/e\beta]}\right). \tag{S23}$$

This is our final lower bound on β_1. Its fulfillment guarantees Eq. (S22) and, therefore, also Eq. (S20).

On the other hand, for $l=1$, Eq. (S19) can be re-written as

$$\begin{aligned}\beta_1 \;&\leq\; \frac{\sqrt{p_\Psi(\beta)}}{2}\beta - \frac{2}{e}\ln(4)\\ &+\;\frac{2}{e}\left(\frac{\sqrt{p_\Psi(\beta)}}{2\ln\left[e+2\ln(1/\varepsilon')/e\beta\right]}-1\right)\ln\left(\frac{1}{\varepsilon'}\right),\end{aligned} \tag{S24}$$

where we have used Eq. (5) again and $p_\Psi(\beta_0)=p_\Psi(0)=1$. For β_1 satisfying Eq. (S23), Eq. (S24) is satisfied if

$$\begin{aligned}\beta \;&\geq\; \frac{2}{\sqrt{p_\Psi(\beta)}}\left[p_\Psi^{-1}\left(\frac{o}{2}\frac{1}{\ln[e+2\ln(2/o\,\varepsilon)/e\beta]}\right)\right.\\ &+\;\left.\frac{2}{e}\ln\left(\frac{4}{\varepsilon'}\right)\right] - \frac{\ln\left(\frac{1}{\varepsilon'}\right)}{e\ln\left[e+2\ln(1/\varepsilon')/e\beta\right]},\end{aligned} \tag{S25}$$

which, in turn, by virtue of Eq. (S15), is satisfied if

$$\beta \geq \frac{2}{o}\left[p_\Psi^{-1}\left(\frac{o}{2}\frac{1}{\ln[e+2\ln(2/o\,\varepsilon)/e\beta]}\right)+\frac{2}{e}\ln\left(\frac{8}{o\,\varepsilon}\right)\right]. \tag{S26}$$

The RHS of Eq. (S26) still depends on β. To remove this dependence, we use a β-independent upper bound for the logarithmic term. First, notice that, for Eq. (S26) to hold, $\beta > \frac{4}{e\,o}\ln\left(\frac{8}{o\,\varepsilon}\right)$ must necessarily hold. This, in turn, is equivalent to $\frac{2\ln(2/o\,\varepsilon)}{e\beta} < \frac{o\ln(2/o\,\varepsilon)}{2\ln(8/o\,\varepsilon)}$; and, since $\frac{o\ln(2/o\,\varepsilon)}{2\ln(8/o\,\varepsilon)} < \frac{o}{2} \leq 1/4$, one gets that $\ln[e+2\ln(2/o\,\varepsilon)/e\beta] < \ln[e+1/4] < 1.1$. Consequently, $\frac{o}{2}\frac{1}{\ln[e+2\ln(2/o\,\varepsilon)/e\beta]} > o/2.2$; and, since p_Ψ^{-1} is a monotonically decreasing function, $p_\Psi^{-1}\left(\frac{o}{2}\frac{1}{\ln[e+2\ln(2/o\,\varepsilon)/e\beta]}\right) < p_\Psi^{-1}(o/2.2)$. That latter is the desired β-independent upper bound. Here, we note that the theorem assumption $o < 1/2.2$ ensures that $p_\Psi^{-1}(o/2.2)$ is well

defined, because it guarantees that $o/2.2 > o^2 = p_\Psi^{-1}(\beta \to \infty)$. Then, finally, taking $\beta_c = \frac{2}{o}\left[p_\Psi^{-1}\left(\frac{o}{2.2}\right) + \frac{2}{e}\ln\left(\frac{8}{o\,\varepsilon}\right)\right]$ and demanding that $\beta > \beta_c$ is sufficient to satisfy Eq. (S26). This is our final lower bound on β.

In conclusion, the schedule $S_2 = \{\beta_1, \beta - \beta_1\}$, with β_1 in the range determined by Eqs. (S23) and (S24), and $\beta \geq \beta_c$, satisfies Eq. (S18) for both $l = 1$ and $l = 2$. In particular, in the theorem statement, β_1 is taken precisely as the RHS of Eq. (S23). This proves the last remaining implication. □

VII. DEPENDENCE ON THE SUCCESS PROBABILITY: FRAGMENTED QITE VERSUS COHERENT MASTER ALGORITHM

Theorem 5 establishes that it is possible to find an inverse temperature and a fragmentation schedule for which fragmented QITE outperforms the coherent master QITE algorithm. Nevertheless, it says nothing about scaling advantages of fragmentation. One of the reasons for that is the intricate dependence of the query complexity in Theorem 4 on all the parameters (β, ϵ, $p_\Psi(\beta)$) not allowing for a clear claim of advantage. More importantly, the complexity depends on the particular fragmentation schedule, which in turn depends on the particular Hamiltonian instance.

However, we can numerically compare the advantage got from amplitude amplification with the results for fragmentation. Compared to repeat until success, amplitude amplification gives a quadratic advantage relatively to the probability of success. That is, while the total query complexity of the probabilistic algorithms is given as $q(\beta,\varepsilon')/p_\Psi(\beta)$, the coherent algorithm has total query complexity of $q(\beta,\varepsilon')/\sqrt{p_\Psi(\beta)}$. In an attempt to isolate the dependence on the success probability, in Fig. S2 we show the ratios $Q_{S_r}(\beta,\varepsilon)/q(\beta,\varepsilon')$ and $Q_{\text{coh}}(\beta,\varepsilon)/q(\beta,\varepsilon')$. The plots show results for different Hamiltonian instances and different values of β larger than the observed average β_c. In this way, we observe the behavior of fragmented QITE after the point for which we get advantage over amplitude amplification. We observe the same scaling for the two master algorithms, but the fragmented algorithm presents a better multiplicative factor. Therefore, from this empirical observation we cannot claim any supra-square advantage of fragmentation over RUS. Nevertheless, we reinforce that the advantage obtained in total query complexity comes without a large circuit depth overhead.

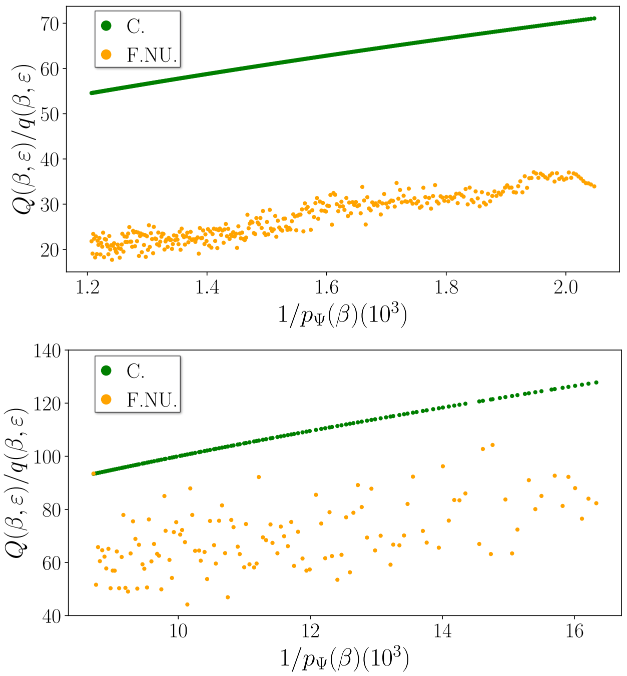

Figure S2. **Dependence of the total query complexities on the probability of success**. Ratio between the total success probability and the query complexity of one run of the probabilistic master algorithm as a function of the inverse of the success probability for two Hamiltonian models. In a) the result for instances of weighted MaxCut for 12 qubits and in b) the result for intances of the Heisenberg fully-connected model with 14 qubits. Each point in the graphs corresponds to a different Hamiltonian instance and one value of inverse temperature above β_c.

VIII. CLOSE-TO-OPTIMALITY OF PRIMITIVE 1 FOR NON-INTERACTING HAMILTONIANS

Let us consider the simple case of a non-interacting qubit Hamiltonian given by

$$H = \sum_j b_j Z_j, \tag{S27}$$

with $b_j \geq 0$ and $\sum_{j=1}^{N} b_j = 1$, such that $\|H\| = 1$. Clearly, the analysis for this case covers also any other Hamiltonian unitarily-equivalent to Eq. (S27). We focus on the task of Gibbs-state sampling (maximally mixed state as input). Hence, $o = 2^{-N/2}$ and the success probability for P_1 is given by $p_\Psi(\beta) = e^{-2\,\beta}\prod_{j=1}^{N}\cosh(2\,\beta\,b_j)$. For simplicity,

we take $b_j = 1/N$ for all j, obtaining

$$p_\Psi(\beta) = e^{-2\,\beta}\cosh(2\,\beta/N)^N. \tag{S28}$$

This is simple enough to obtain a closed-form expression for its inverse function and – so – explicit expressions for the fragmentation schedule of Lemma 5, which we do next.

By virtue of Lemma 5, $\Delta\beta_1 = p_\Psi^{-1}\big(\frac{o}{2}\frac{1}{\ln[e+2\ln(2/o\,\varepsilon)/e\beta]}\big)$. Hence, using that $1 < \ln[e+2\ln(2/o\,\varepsilon)/e\beta] < 1.1$, we obtain $p_\Psi^{-1}\big(\frac{o}{2}\big) = \frac{N}{4}\ln\big(\frac{2^{1/N}}{2^{1/2}-2^{1/N}}\big) < \Delta\beta_1 < p_\Psi^{-1}\big(\frac{o}{2.2}\big) = \frac{N}{4}\ln\big(\frac{(2.2)^{1/N}}{2^{1/2}-(2.2)^{1/N}}\big)$. This, using that, for $N \geq 3$, $\ln\big(\frac{(2.2)^{1/N}}{2^{1/2}-(2.2)^{1/N}}\big) \leq 2.44$ and $\ln\big(\frac{2^{1/N}}{2^{1/2}-2^{1/N}}\big) > 0.88$, gives

$$0.88\,\frac{N}{4} \leq \Delta\beta_1 \leq 2.44\,\frac{N}{4} \tag{S29}$$

and $\beta_c = 2^{N/2+1}\big[2.44\,\frac{N}{4} + \ln\big(8\frac{2^{N/2}}{\varepsilon}\big)\big]$. This, in turn leads to

$$\Delta\beta_2 \geq 2^{N/2+1}\Big[2.44\,\frac{N}{4} + \ln\Big(8\frac{2^{N/2}}{\varepsilon}\Big)\Big] - 2.44\,\frac{N}{4} \tag{S30}$$

for all $\beta \geq \beta_c$. The case $N = 2$ is not covered by Lemma 5 because it has $o = 1/2 > 1/2.2$.

As clear from Eqs. (S29) and (S30), the first fragment is exponentially shorter in N than the second one. Moreover, we show next that, the first fragment satisfies $\beta_1 < 8\ln(4/\varepsilon_1')$. This implies that Primitive 1 performs better (in query complexity) than the one from Ref. [7] at the fragmentation scheme from Lemma 5, as discussed after Theor. 1. In fact, it also implies that Primitive 1's performance is close to optimal (see discussion after Theor. 3) at that first fragment. From Eq. (5), for $r = 2$, we have $\varepsilon_1' = \varepsilon\sqrt{p_\Psi(\beta)}/8$. Now, because $\beta > p_\Psi^{-1}(o/2.2)$, $p_\Psi(\beta) < o/2.2 = 2^{-N/2}/2.2$. This leads to

$$\begin{aligned} 8\ln\left(\frac{4}{\varepsilon_1{}'}\right) &= 8\ln\left(\frac{4\times 8}{\varepsilon\sqrt{p_\Psi(\beta)}}\right) \\ &> 8\ln\left(\frac{4\times 8}{\varepsilon\sqrt{2^{-N/2}/2.2}}\right) \\ &> 8\Big[\ln\big(2^{N/4}\big) + \ln\Big(\frac{21}{\varepsilon}\Big)\Big] \\ &> 2.44\,\frac{N}{4} + 8\ln\Big(\frac{21}{\varepsilon}\Big) \\ &> \beta_1, \end{aligned} \tag{S31}$$

where, in the last equation, we have used Eq. (S29) and the fact that $\beta_1 = \Delta\beta_1$. This finishes the proof.

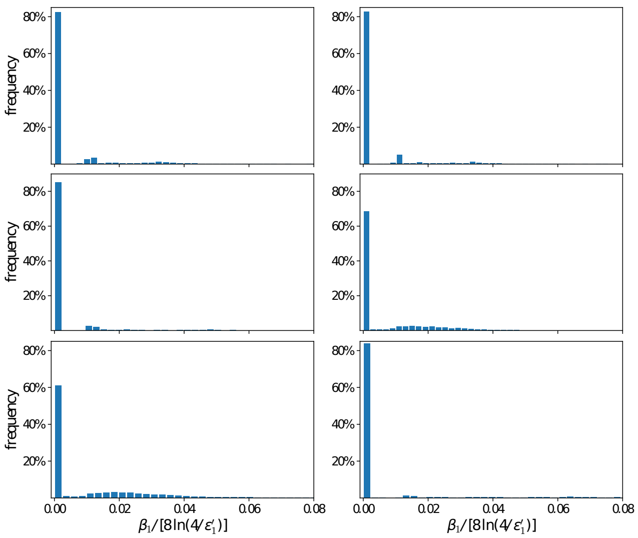

Figure S3. **Histograms of $\beta_1/8\ln(4/\varepsilon_1')$ for the optimized schedules $S_{r,a}$ from Eq.** (7). The upper panels correspond to the quantum spin glass Hamiltonians with $N = 14$ (left) and $N = 15$ (right) qubits, the central ones to the quatum RBM with $N = 4$ (left) and $N = 5$ (right), and the lower ones to the weighted Max-Cut with $N = 12$ (left) and $N = 13$ (right). Similar behaviours are observed for other values of N and ε. For the upper and central panels we took $\varepsilon = 0.001$, whereas for the lower ones $\varepsilon = 0.01$. For each Hamiltonian instance H, we took several different values of β; and each pair (H, β) constitutes a different event in the histogram. For the panels where $N \leq 12$, the histograms are built from 81 uniformly chosen values of β from 0 to 2000 for each of the 1000 Hamiltonian instances in each class, giving a total of 81000 events. Whereas for those where $N > 12$, they are built from 21 uniformly chosen values of β from 0 to 10000 and 100 Hamiltonian instances from each class, giving a total of 2100 events. The results show that β_1 is typically 1000 (and at worst 20) times smaller than $8\ln(4/\varepsilon_1')$. This confirms that the first fragment operates deep into the regime of optimality of P_1 even for uniformly sampled β. If, instead of uniform values of β between 0 and the high values mentioned above, we choose β values close to the corresponding critical inverse temperature, then $\beta_1/8\ln(4/\varepsilon_1')$ concentrates even more at the first column close to zero.

IX. CLOSE-TO-OPTIMALITY OF PRIMITIVE 1 FOR INTERACTING HAMILTONIANS

Here, we study the ratio between $\Delta\beta_1 = \beta_1$ and $8\ln(4/\varepsilon_1')$ for fragmented quantum-Gibbs-state sampling with P_1, for the generic Hamiltonians studied in Sec. 2.4

and the optimal schedules $S_{r,a}$ used for the central panel of Fig. 5. Recall that $\beta_1 \approx 8\ln(4/\varepsilon_1')$ is the point at which the complexity upper bound in Eq. (1) starts outperforming the complexity upper bound derived in [7] for the powerful QITE primitive obtained there, as mentioned after Theo. 1. The results are displayed in Fig. S3, showing the histograms of $\beta_1/8\ln(4/\varepsilon_1')$ for the random instances considered for the three classes of Hamiltonian and for different N. This clearly shows that $\beta_1 \ll 8\ln(4/\varepsilon_1')$, supporting our claim that, for the first fragment, the fragmented master algorithm operates deep into the optimality regime of Primitive 1. Finally, we performed the same analysis for $\Delta\beta_2$ (not shown) and consistently observed that $\Delta\beta_2$ is smaller than $8\ln(4/\varepsilon_2')$ too. That is, also the second fragment operates close to the optimality regime of P_1.

X. FRAGMENTED GIBBS-STATE SAMPLING WITH P_1 ON THE 10% HARDEST INSTANCES

Here, we analyze the performance of P_1 for Gibbs-state sampling but restricted to the 10% hardest Hamiltonian instances from those used to produce Fig. 3. The hardest instances are given by the choices of H with the smallest spectral gap Δ between the first excited and the ground states. This is due to the fact that the inverse temperature required to approximate the ground state (up to any constant target precision) scales as $\beta = \mathcal{O}(1/\Delta)$. That is, the lower the gap is, the higher the query complexity is.

The analysis is shown in Fig. S4. Apart from the average run-time and circuit depth, as in Fig. 3, we also plot the evolution of the average post-selection probability and fidelity with the ground state. Both post-selection probabilities and ground-state fidelities are calculated via brute-force diagonalization of each Hamiltonian. As can be seen in the figure, for Weighted MaxCut and, especially, Quantum Spin Glasses, fragmented QITE becomes superior to coherent QITE well before the value $\beta_{0.9}^{(\text{hard})}$ needed for ground state preparation up to a modest fidelity 0.9. In turn, for Quantum RBMs, fragmented QITE becomes superior to coherent QITE after $\beta_{0.9}^{(\text{hard})}$, but the difference in their query complexity at $\beta_{0.9}^{(\text{hard})}$ is already very small. If, instead, the target fidelity is raised to 0.99, all three classes require inverse temperatures greater than β_{c}. This implies that fragmented QITE is superior to coherent QITE for ranges of β that are highly relevant for ground state preparation.

XI. FRAGMENTED GIBBS-STATE SAMPLERS WITH P_2

Here, we numerically study the performance of Alg. 1 at quantum Gibbs-state sampling, as in Sec. 2.4, but for Primitive 2. In Fig. S5 we show the average runtimes and circuit depths. The schedule optimization algorithms follow the same approach as for P_1. The critical inverse temperatures for P_2 are shown in Fig. S6. For P_2 we achieve good optimization stability only from larger values of N. This can be understood from the fact that for small N the minimum success probability (2^N) is relatively large. Because the subnormalization factors α_k of each fragment only depend on the inverse temperature (not on N) and has a minimum value, the optimization seeks for large β such that the success probability of each fragment approaches 2^N. Finally, in Fig. S7 we show the optimal fragmentation schedules for P_2.

XII. QSP ACHIEVABILITY – METHOD 1

The following Lemma (adapted from Theorem 5 of Ref. [7]) presents the set of conditions for a pair of real polynomials $\mathscr{B}(\cos\theta)$ and $\mathscr{D}(\cos\theta)$ to be achieved as the real/imaginary parts of $B(\cos\theta)$ and $D(\cos\theta)$ of Eq. (8). This conditions have been summarised in Eqs. (9) and (10) in the main text.

Lemma 4. *Given two polynomials $\mathscr{B}(x)$, $\mathscr{D}(x) : [-1,1] \to \mathbb{R}$, and $q \in \mathbb{N}$ even, there exists $\mathbf{\Phi}_1 = (\phi_1, \cdots, \phi_{q+1}) \in \mathbb{R}^{q+1}$ such that $\mathscr{B}(\cos\theta) = Re[B(\cos\theta)]$ (or $\mathscr{B}(\cos\theta) = Im[B(\cos\theta)]$) and $\mathscr{D}(\cos\theta) = Re[D(\cos\theta)]$ (or $\mathscr{D}(\cos\theta) = Im[D(\cos\theta)]$) for all $\theta \in [-\pi,\pi]$, with B and D as in Eq.* (8)*, if and only if $\mathscr{B}$ and $\mathscr{D}$ satisfy:*

(i) $\mathscr{B}(x) = \sum_{k=0}^{q/2} b_k' x^{2k}$ *and* $\mathscr{D}(x) = \sum_{k=0}^{q/2-1} d_k' x^{2k+1}$*;*

(ii) $\forall x \in [-1,1]: \quad \mathscr{B}^2(x) + \left(1-x^2\right)\mathscr{D}^2(x) \leq 1.$

Moreover, the achievable functions can also be expressed as $\mathscr{B}(\cos\theta) = \sum_{k=0}^{q/2} b_k \cos(2k\theta)$ and $\sin\theta\,\mathscr{D}(\cos\theta) = \sum_{k=1}^{q/2} d_k \sin(2k\theta)$.

The last part of the Lemma, presented before in Eq. (10), can be obtained from condition (i) using the properties of the Chebyshev polynomials $T_k(\cos\theta) = \cos(k\theta)$ and $U_k(\cos\theta) = \sin((k+1)\theta)/\sin\theta$. Condition (ii) is equivalent to Eq. (9) in the main text. Given that the desired polynomials are achievable by QSP, the set of rotation angles $\mathbf{\Phi}_1$ can be computed classically in time $\mathcal{O}(\text{poly}(q))$ [9–12].

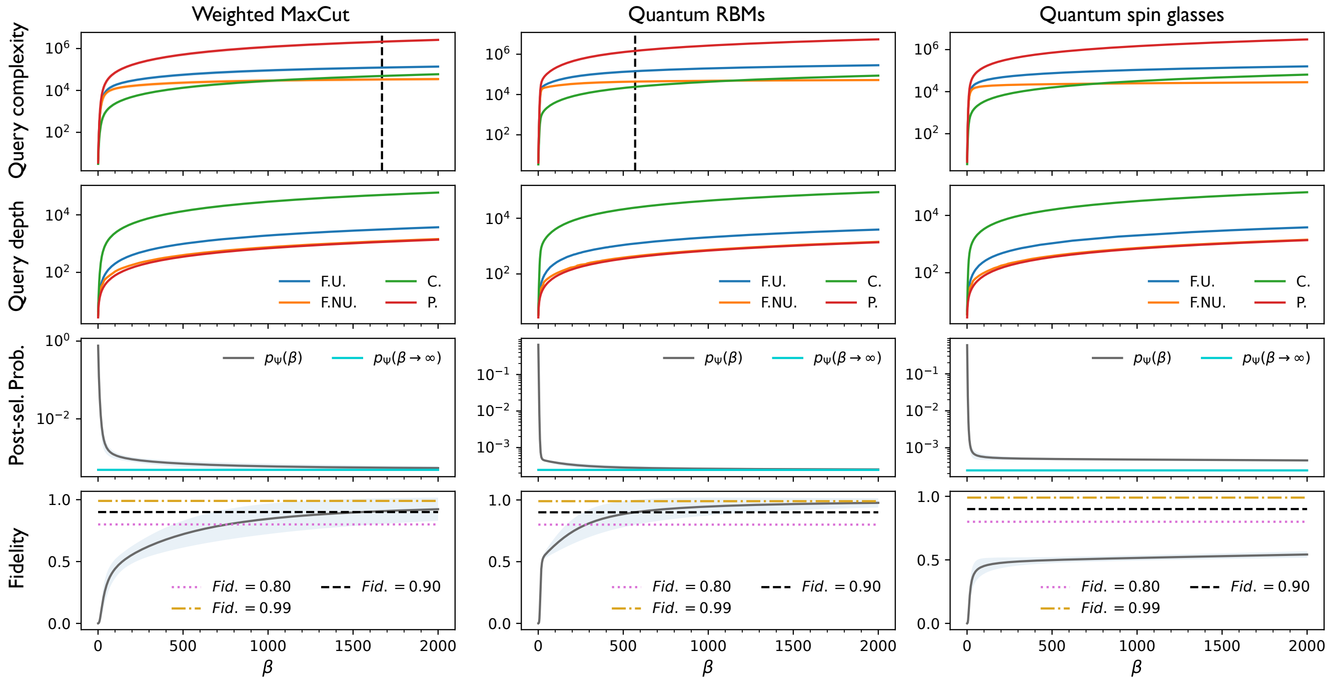

Figure S4. **Quantum Gibbs-state samplers running on Primitive 1 for the 10% hardest Hamiltonian instances: runtime, circuit depth, success probability, and fidelity with the ground state versus inverse temperature**. The Hamiltonian classes are the same as in Fig. 3; and the number of qubits and tolerated error are also $N = 12$ and $\varepsilon = 10^{-3}$, respectively. Out of the 1000 random instances used for each class in Fig. 3, we select the 100 ones with the smallest spectral gap. Solid curves represent the means over that 10%. The color code is the same as in Fig. 3 too: red for probabilistic, green for coherent, blue for fragmentation with uniform schedule S_r for the best r, and orange for fragmentation with a schedule $S_{r,a}$ as in Eq. (7) for the best r and a. From top to bottom: the upper panels show the overall query complexity, the second row the average query depth, the third one the post-selection probability, and the lower pannels the average fidelity between the ideal Gibbs state at inverse temperature β and the ground state in question. The black vertical dashed lines in the first row represent the value $\beta_{0.9}^{(\text{hard})}$ of inverse temperature required for a modest average fidelity of 0.9 with the ground state, defined by the intersection between the black horizontal dashed lines and the blue solid curves in the last row. In the third panel of the first row there is no black vertical dashed line because, for this class, $\beta_{0.9}^{(\text{hard})}$ is way beyond the range of β's shown, as is clear also from the third panel of the last row. In the first row, both for the first and third panels, the critical value β_c at which fragmentation outperforms coherent QITE is below $\beta_{0.9}^{(\text{hard})}$; whereas, for the second panel, $\beta_{0.9}^{(\text{hard})}$ is smaller than β_c but the difference in query complexity at $\beta_{0.9}^{(\text{hard})}$ is already very small. If the target fidelity is instead 0.99, all three classes require inverse temperatures greater than β_c. This implies that fragmented QITE is superior to coherent QITE for highly-relevant ranges of β for ground state preparation, e.g. Interestingly, in addition, the complexities (and therefore also β_c) and depths shown in the first and second rows are very similar to those of the average case in Fig. 3. In the third row, to guide the eye, the cyan solid line shows the asymptotic limit $p_\Psi(\beta \to \infty)$ of the post-selection probability. These plots give a first glimpse of how far away from ground state the Gibbs-state in question is. Finally, in the last row we make this observation more quantitative with the fidelity as figure of merit. There, horizontal dashed lines represent the fidelity values 0.8 (pink), 0.9 (black), and 0.99 (gold).

XIII. CHEBYSHEV EXPANSIONS

In this appendix we briefly approach polynomial approximations to continuous functions using Chebyshev polynomials.

It is known that [13], whenever a function f is continuous and bounded on the interval $[-1, 1]$, it is endowed with a

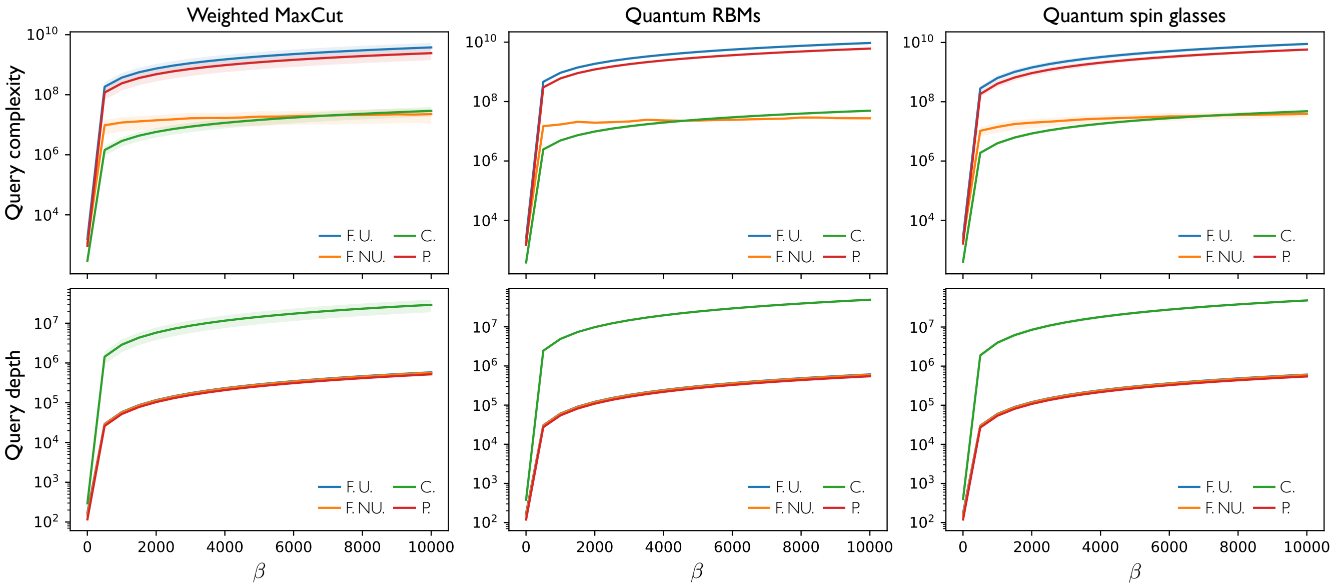

Figure S5. **Runtimes and circuit depths of quantum Gibbs-state samplers running on Primitive 2 versus inverse temperature**. The color code is the same as in Fig. 3: red corresponds to probabilistic, green to coherent, blue to fragmented with uniform schedule S_r for the best r, and orange to fragmented with a schedule $S_{r,a}$ as in Eq. (7) for the best r and a (see also Fig. S7). The Hamiltonian classes are also the same as in Fig. 3. For simplicity, here we use just a subset of 100 random instances from each class out of the 1000 used for Fig. 3. The examples correspond to $N = 12$ qubits and an error $\varepsilon = 10^{-3}$, but qualitatively identical behaviors are observed for all N between 2 and 15 as well as for $\varepsilon = 10^{-2}$ and $\varepsilon = 10^{-1}$. Upper panels: average overall query complexity. As mentioned, also for P_2 does fragmentation with non-uniform schedule outperform coherent QITE, except the critical inverse temperature β_c is now higher than in Fig. 3. The dependance of β_c with N is shown and discussed in Fig. S6. Lower panels: average query depth. As with P_1, also for P_2 does coherent QITE lie orders of magnitude above probabilistic QITE; while fragmented QITE is almost identical to the latter.

convergent Chebyshev series such that

$$f(\lambda) = \sum_{k=0}^{\infty} b_k T_k(\lambda), \tag{S32}$$

with coefficients

$$b_0 = \frac{1}{\pi}\int_{-1}^{1} \frac{f(\lambda)}{\sqrt{1-\lambda^2}} d\lambda \tag{S33}$$

$$b_k = \frac{2}{\pi}\int_{-1}^{1} \frac{f(\lambda)T_k(\lambda)}{\sqrt{1-\lambda^2}} d\lambda, \quad k = 1, 2, \cdots \tag{S34}$$

The truncated version of (S32) up to order q is close to the optimal polynomial approximation of degree q for $f(\lambda)$ in the interval $[-1, 1]$ [13]. The following Lemma (Theorem 2.1 from [14]) gives the maximal error ε of this approximation for a given degree q.

Lemma 5. *Let $\tilde{f}(\lambda)$ be the polynomial approximation on $[-1, 1]$ to a function $f \in C^{(q+1)}[-1, 1]$ obtained by truncating its Chebyshev expansion up to order q. Then*

$$\varepsilon = \max_{-1\leq\lambda\leq 1} |f(\lambda) - \tilde{f}(\lambda)| = \frac{\left|f^{(q+1)}(\xi)\right|}{2^q (q+1)!}, \tag{S35}$$

for some $\xi \in (-1, 1)$.

Another way of obtaining a nearly optimal polynomial approximation of order q is to construct it from Chebyshev polynomials interpolation, which can be done in polynomial time by solving a linear system [14]. The latter construction is based on conveniently choosing a list of points $\lambda_0, \cdots, \lambda_q$, typically related to the zeros or extreme points of $T_{q+1}(\lambda)$, and finding the linear combination of all degree-less-than-q Chebyshev polynomials which interpolates $f(\lambda_0), \cdots, f(\lambda_q)$. The error formula of Eq. (S35) is not exclusive of the Chebyshev truncated series. It is also

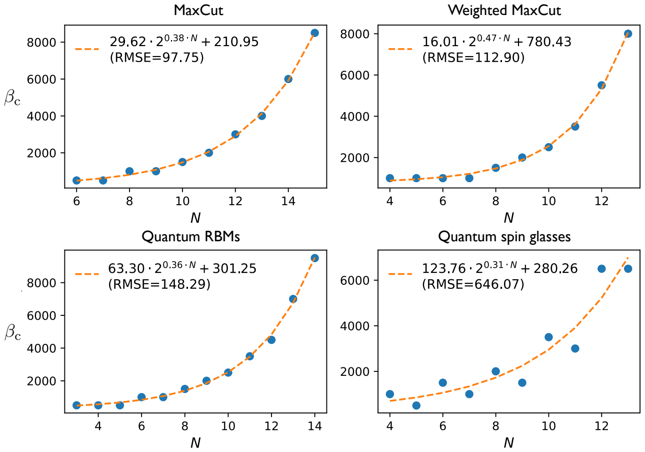

Figure S6. **Critical inverse temperatures for P_2 versus number of qubits**. The tolerated error and Hamiltonian classes are the same as in Fig. 4. Blue dots are the means over 100 instances from each class, whereas dashed orange curves their fits over the same Ansatz as in Fig. 4. The fit results are shown in the insets. The scalings of $\beta_{\rm c}$ with N are similar to those for P_1 but with somewhat higher pre-factors and additive constants. The fact that $\beta_{\rm c}$'s are higher than for P_1 comes from the non-unit factors α_k inside n_l in Eq. (6).

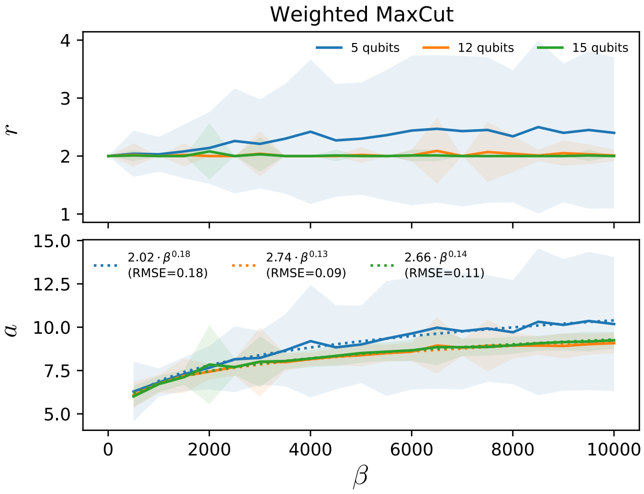

Figure S7. **Optimal fragmentation schedules for P_2 versus inverse temperature**. System sizes and color code are the same as in Fig. 5: $N = 5$ (blue), $N = 12$ (orange), and $N = 15$ (green). Solid curves represent the means over 100 random weighted-MaxCut Hamiltonians, whereas (the thicknesses of) shaded curves are the standard deviations. The error is $\varepsilon = 10^{-3}$. Qualitatively identical behaviors are observed for all N between 2 and 15 as well as for $\varepsilon = 10^{-2}$ and $\varepsilon = 10^{-1}$; and the same holds for the other Hamiltonian classes. For uniform schedules $S_{r,1}$ (not shown in the figure), a constant $r = 2$ is observed to minimize Q_{S_r} but the resulting complexity does not reach $Q_{\rm coh}$ over the domain scanned in Fig. S5. The upper and lower panels respectively show the optimal r and a for non-uniform schedules $S_{r,a}$. The dotted curves in the lower panel represent fits over the Ansatz $a(\beta) = A\,\beta^{\eta}$, with $A, \eta \in \mathbb{R}$. The fit results are shown in the inset. As with P_1, also here is r constant not only with β but also with N, remarkably.

valid for the special interpolating polynomials just described and for the optimal polynomial approximation, the difference being the point ξ where the $(n+1)$-th derivative must be evaluated.

XIV. A RECIPE FOR PULSES – QSP METHOD 1

This appendix is based on the proof of Theorem 3 of Ref. [7]. We restrict ourselves to the case of even q, as this is what we use throughout our operator-function design algorithm. Henceforth, we write $x = \cos\theta$. Here we only show how to get the QSP pulse sequence $\mathbf{\Phi}$ given the complex polynomials $B(x)$ and $D(x)$, such that Eq. (8) is attained. We refer the reader to Theorem 5 of Ref. [7] for a proof of existence of B and D given the real polynomials $\mathscr{B}(x)$ and $\mathscr{D}(x)$ satisfying Lemma 4. The proof of the referred theorem is also an algorithm to calculate B and D whose classical computational runtime scales as $\mathscr{O}(\text{poly}(q))$.

Given two polynomials $B(x)$ and $D(x)$ as in Eq. (8), it can be checked from multiplying single qubit rotation operators that they satisfy:

i) $\deg(B) \leq q$ and $\deg(D) \leq q-1$;

ii) B is even and D is odd;

iii) $\forall x \in [-1,1]: \quad |B(x)|^2 + (1-x^2)|D(x)|^2 = 1.$

Now we build a recurrence which allows one to find a pulse sequence $\mathbf{\Phi} = (\phi_1, \cdots, \phi_{q+1})$ such that, given two polynomials

$$B(x) = \sum_{k=0}^{l/2} b_k x^{2k} \quad \text{and} \quad D(x) = \sum_{k=0}^{l/2-1} d_k x^{2k+1} \qquad \text{(S36)}$$

satisfying the three above conditions – thus $l \leq q$ – they are obtained as the entries of the operator $\mathcal{R}_1(\theta, \mathbf{\Phi}) = e^{i\phi_{q+1}Z} \prod_{k=1}^{q/2} R_1(-\theta, \phi_{2k}) R_1(\theta, \phi_{2k-1})$. Observe that, if

$l = 0$, then actually B is constant and condition iii) implies that $D(x) = 0$ and $|B(x)| = 1$. Thus, we can write $B(x) = e^{i\phi}$ and the pulse sequence $\mathbf{\Phi} = (\phi, 0, \cdots, 0)$ does the job.

Because the pair of polynomials satisfies condition iii), they always define a unitary operator

$$\mathcal{R}_{BD} = \begin{pmatrix} B(x) & i\sqrt{1-x^2}D(x) \\ i\sqrt{1-x^2}D^*(x) & B^*(x) \end{pmatrix}. \quad \text{(S37)}$$

Let us also define the new polynomials $\tilde{B}(x)$ and $\tilde{D}(x)$ by

$$\begin{pmatrix} \tilde{B}(x) & i\sqrt{1-x^2}\tilde{D}(x) \\ i\sqrt{1-x^2}\tilde{D}^*(x) & \tilde{B}^*(x) \end{pmatrix} = \mathcal{R}_{BD} R_1(-\theta, -\phi_1), \quad \text{(S38)}$$

with $\theta = \cos^{-1}(x)$. Performing the operator multiplications, one gets

$$\begin{aligned} \tilde{B}(x) &= e^{-i\phi_1}\left[xB(x) + e^{2i\phi_1}(1-x^2)D(x)\right] \\ \tilde{D}(x) &= e^{-i\phi_1}\left[-B(x) + e^{2i\phi_1}xD(x)\right]. \end{aligned} \quad \text{(S39)}$$

It is easy to check that defining $e^{2i\phi_1} = \frac{b_{l/2}}{d_{l/2-1}}$ makes the higher order terms in both $\tilde{B}(x)$ and $\tilde{D}(x)$ vanish. This is a valid choice for ϕ_1 because, if $l > 0$, condition iii) implies that the polynomial $|B(x)|^2 + (1-x^2)|D(x)|^2$ is constant, and thus each of its coefficients accompanying a greater-than-zero power of x must vanish. In particular, it holds for the highest order term if $\left|\frac{b_{l/2}}{d_{l/2-1}}\right| = 1$. Hence, the coefficients of $B(x)$ and $D(x)$ can be rearranged to give

$$\tilde{B}(x) = \sum_{k=0}^{l/2-1} \tilde{b}_k x^{2k+1} \quad \text{and} \quad \tilde{D}(x) = \sum_{k=0}^{l/2-2} \tilde{d}_k x^{2k}, \quad \text{(S40)}$$

which are an odd degree $l-1$ and an even degree $l-2$ polynomials, respectively. Thus, the right multiplication of $R_1(-\theta, -\phi_1)$ has the power of decreasing the degree of the polynomials in $\mathcal{R}_{BD}$ by one. Because $\tilde{B}(x)$ and $\tilde{D}(x)$ compose a unitary operator, they also satisfy condition iii). Therefore, the aforementioned argument can be used again to verify that $|\tilde{b}_{l/2-1}| = |\tilde{d}_{l/2-2}|$ and define $e^{2i\phi_2} = -\frac{\tilde{b}_{l/2-1}}{\tilde{d}_{l/2-2}}$. Multiplying Eq. (S38) from the right by $R_1(\theta, -\phi_2)$ cancels out the highest order terms again and results in an operator

$$\mathcal{R}_{\tilde{\tilde{B}}\tilde{\tilde{D}}} = \begin{pmatrix} \tilde{\tilde{B}}(x) & i\sqrt{1-x^2}\tilde{\tilde{D}}(x) \\ i\sqrt{1-x^2}\tilde{\tilde{D}}^*(x) & \tilde{\tilde{B}}^*(x) \end{pmatrix} \quad \text{(S41)}$$

whose entries are polynomials with $\deg(\tilde{\tilde{B}}) = l/2 - 2$ and $\deg(\tilde{\tilde{D}}) = l/2 - 3$. This procedure, alternating the sign of θ, can be repeated l times. In each step, the rotation angle ϕ_k is determined from the highest degree coefficients of the polynomials from previous step and a rotation $R_1(\pm\theta, -\phi_k)$ is applied. It decreases the degree of the polynomials by one, until we finally arrive at a constant polynomial which determines the last phase ϕ_{l+1} for the Z rotation which is applied along with no X rotation. If $l < q$, then $\phi_{l+2} = \cdots = \phi_{q+1} = 0$ and we can always choose $q = l$ and not waste pulses. This algorithm provides the exact pulses using $\mathcal{O}(q^2)$ multiplications and additions.

XV. QUBITIZATION

As mentioned in the main text, it is possible to transform a block-encoding oracle of a Hamiltonian into a new block-encoding under which each eigenvalue of H is associated with an invariant subspace with dimension two. This transformation is called qubitization of the oracle and is the subject of this appendix.

Consider a unitary U_H perfectly block-encoding a Hamiltonian H using $|\mathcal{A}_{U_H}|$ ancillas. Recall that $|\lambda\rangle \in \mathbb{H}_\mathcal{S}$ is the λ-th eigenstate of H. Then the oracle O_1 of Def. 2 obtained from the action of U_H controled by a single-qubit ancilla $\mathcal{A}_{\text{ctrl}}$ is also a perfect block-encoding of H. The total number of ancillas used by this oracle is $|\mathcal{A}_{O_1}| = |\mathcal{A}_{U_H}| + |\mathcal{A}_{\text{ctrl}}|$. Denoting $|0_\lambda\rangle = |\lambda\rangle|0\rangle \in \mathbb{H}_{\mathcal{S}A_{O_1}}$, the action of O_1 produces $O_1|0_\lambda\rangle = \lambda|0_\lambda\rangle + \sqrt{1-\lambda^2}|0_\lambda^\perp\rangle$, with $|0_\lambda^\perp\rangle \in \mathbb{H}_{\mathcal{S}\mathcal{A}_{O_1}}$ and $\langle 0_\lambda|0_\lambda^\perp\rangle = 0$. Although $O_1|0_\lambda\rangle$ is in the subspace spanned by $\{|0_\lambda\rangle, |0_\lambda^\perp\rangle\}$, further applications of O_1 will not in general produce higher powers of H as the state will leak out of this subspace. In order to avoid this, a convenient oracle for producing Hamiltonian functions would preserve the subspaces $\mathbb{H}_\lambda := \text{span}\{|0_\lambda\rangle, |0_\lambda^\perp\rangle\} \subset \mathbb{H}_{\mathcal{S}\mathcal{A}_{O_1}}$ for each eigenvalue λ. It was shown in ref. [6] that it is always possible to obtain from O_1 its qubitized version O_1' which also block-encodes the Hamiltonian, i.e. $\langle 0|O_1'|0\rangle = H$ with $|0\rangle \in \mathbb{H}_{\mathcal{S}\mathcal{A}_{O_1}}$, and can be represented as

$$O_1' = \bigoplus_\lambda \left[e^{-i\theta_\lambda}|0_{\lambda+}\rangle\langle 0_{\lambda+}| + e^{i\theta_\lambda}|0_{\lambda-}\rangle\langle 0_{\lambda-}|\right], \quad \text{(S42)}$$

in the subspace $\bigoplus_\lambda \mathbb{H}_\lambda$ of $\mathbb{H}_{\mathcal{S}\mathcal{A}_{O_1}}$. Here $\theta_\lambda = \cos^{-1}(\lambda)$, and $|0_{\lambda\pm}\rangle = \left(|0_\lambda\rangle \pm i|0_\lambda^\perp\rangle\right)/\sqrt{2}$, such that $\langle 0_{\lambda+}|0_{\lambda-}\rangle = 0$. As can be noticed, for any λ, the subspace $\mathbb{H}_\lambda$ is invariant under O_1'. Furthermore, in that two-dimension subspace, O_1' is

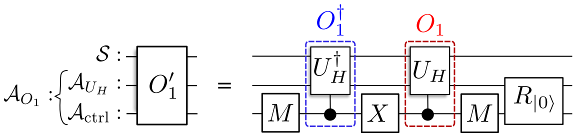

Figure S8. Circuit implementing the transformation of the Hamiltonian oracle O_1 of Def. 2 into its qubitized version O_1'. It requires one use of O_1 and one of its inverse, three single qubit gates applied to the control ancilla $\mathcal{A}_{\mathrm{ctrl}}$, and $\mathcal{O}(|\mathcal{A}_{O_1}|)$ additional gates to implement the reflection $R_{|0\rangle}$ about the ancilla state $|0\rangle \in \mathbb{H}_{\mathcal{A}_{O_1}}$.

isomorphic to the qubit rotation $e^{-i\theta_\lambda Y_\lambda}$, with Y_λ the second Pauli operator in $\mathbb{H}_\lambda$.

Applying O_1' uses only one query to O_1 and one to $O_1^\dagger$, and $\mathcal{O}(|\mathcal{A}_{O_1}|)$ additional quantum gates. The precise prescription given in [6] for obtaining O_1' as in Eq. (S42) is: take the operators $U_H' = O_1 \otimes |+\rangle\langle +| + O_1^\dagger \otimes |-\rangle\langle -|$, $R_{|0\rangle} = \mathbb{1}_\mathcal{S} \otimes \left(2|0\rangle\langle 0| - \mathbb{1}_{\mathcal{A}_{O_1}}\right)$, and $S = \mathbb{1}_{\mathcal{S}\mathcal{A}_{U_H}} \otimes (MXM)$ on $\mathbb{H}_{\mathcal{S}\mathcal{A}_{O_1}}$, then

$$O_1' = R_{|0\rangle} S U_H'. \tag{S43}$$

A equivalent circuit representation of O_1' is found in Fig. S8. We refer the reader to Ref. [6] for a demonstration of the equality between Eqs. (S42) and (S43).

XVI. PROOF OF LEMMA 9

In order to prove Lemma 9, we first announce and prove the following lemma, which states how to implement operator Chebyshev series in general.

Lemma 6. *(Chebyshev series from block-encoding Hamiltonian oracles) Given $\tilde{f}_q(\lambda) = \sum_{k=0}^{q/2} b_k T_k(\lambda)$, with $b_k \in \mathbb{R}$, such that $|\tilde{f}_q(\lambda)| \leq 1$ for all $\lambda \in [-1,1]$, there is a $\mathbf{\Phi}_1 = (\phi_1, \cdots, \phi_{q+1}) \in \mathbb{R}^{q+1}$, such that the unitary operator defined in Eq.* (14) *and implemented by Alg. 2 is a perfect block-encoding of $\tilde{f}_q(H)$, i.e.*

$$\langle 0|V_{\mathbf{\Phi}_1}|0\rangle = \sum_\lambda \tilde{f}_q(\lambda)\, |\lambda\rangle\langle\lambda|, \tag{S44}$$

with $|0\rangle$ in $\mathbb{H}_\mathcal{A}$. Moreover, the pulse sequence can be obtained classically in time $\mathcal{O}(poly(q))$.

Proof of Lemma 6. Recalling that the identity operator does not produce any leakage of states out of the initial subspace they belong to, the identity operator in $\mathbb{H}_{\mathcal{S}\mathcal{A}_{O_1}}$ can be effectively represented on the subspace $\bigoplus_\lambda \mathbb{H}_\lambda$ as $\mathbb{1} = \bigoplus_\lambda \left(|0_{\lambda+}\rangle\langle 0_{\lambda+}| + |0_{\lambda-}\rangle\langle 0_{\lambda-}|\right)$. Using this representation and Eq. (S42), it is straightforward to write the operator V_0 of Eq. (13) as

$$V_0 = \bigoplus_\lambda e^{-i\frac{\theta_\lambda}{2}} |0_{\lambda+}\rangle\langle 0_{\lambda+}| \otimes e^{i\frac{\theta_\lambda}{2}X} + e^{i\frac{\theta_\lambda}{2}} |0_{\lambda-}\rangle\langle 0_{\lambda-}| \otimes e^{-i\frac{\theta_\lambda}{2}X}, \tag{S45}$$

where X is the first Pauli operator for an extra single-qubit ancilla we will denote by $\mathcal{A}_e$. From V_0 one can also obtain a convenient representation for $V_\phi = V_0\left(\mathbb{1} \otimes e^{i\phi Z}\right)$ in $\bigoplus_\lambda \mathbb{H}_\lambda \otimes \mathbb{H}_{\mathcal{A}_e}$, it reads

$$V_\phi = \bigoplus_\lambda \left(e^{-i\frac{\theta_\lambda}{2}} |0_{\lambda+}\rangle\langle 0_{\lambda+}| \otimes R_1\left(\frac{\theta_\lambda}{2}, \phi\right) + e^{i\frac{\theta_\lambda}{2}} |0_{\lambda-}\rangle\langle 0_{\lambda-}| \otimes R_1\left(-\frac{\theta_\lambda}{2}, \phi\right)\right), \tag{S46}$$

with the qubit rotations acting on $\mathcal{A}_e$ previously defined as $R_1\left(\pm\frac{\theta_\lambda}{2}, \phi\right) = e^{\pm i\frac{\theta_\lambda}{2}X} e^{i\phi Z}$. If the operator defined as $\bar{V}_{\phi'} = V_0^\dagger\left(\mathbb{1}_{\mathcal{S}\mathcal{A}_O} \otimes e^{i\phi' Z}\right)$ is applied in the sequence, it removes the undesired phase factors $e^{\pm i\frac{\theta_\lambda}{2}}$. A direct consequence of Eq. (S46) is that the operator $V_{\mathbf{\Phi}_1}$ defined in Eq. (14) can be represented in $\bigoplus_\lambda \mathbb{H}_\lambda \otimes \mathbb{H}_{\mathcal{A}_e}$ as

$$V_{\mathbf{\Phi}_1} = \bigoplus_\lambda \left[|0_{\lambda+}\rangle\langle 0_{\lambda+}| \otimes \left(M\,\mathcal{R}_1\left(\frac{\theta_\lambda}{2}, \mathbf{\Phi}_1\right) M\right) + |0_{\lambda-}\rangle\langle 0_{\lambda-}| \otimes \left(M\,\mathcal{R}_1\left(-\frac{\theta_\lambda}{2}, \mathbf{\Phi}_1\right) M\right)\right], \tag{S47}$$

where $\mathcal{R}_1\left(\pm\frac{\theta_\lambda}{2}, \mathbf{\Phi}_1\right)$ is given in Eq. (8). In order to obtain functions with vanishing imaginary part, one can initialize and post-select the ancillas in the state $|0\rangle \in \mathbb{H}_\mathcal{A}$, which corresponds in $\mathbb{H}_\mathcal{S}$ to applying the operator

$$\langle 0|V_{\mathbf{\Phi}_1}|0\rangle = \sum_\lambda \mathrm{Re}\left[B\left(\cos\frac{\theta_\lambda}{2}\right)\right] |\lambda\rangle\langle\lambda|, \tag{S48}$$

where $B\left(\cos\frac{\theta_\lambda}{2}\right)$ is the the first matrix element of $\mathcal{R}_1\left(\frac{\theta_\lambda}{2}, \mathbf{\Phi}_1\right)$. Because of the halved angle, the achievable functions can be written, according to Lem. 4, as

$$\mathrm{Re}\left[B\left(\cos\frac{\theta_\lambda}{2}\right)\right] = \sum_{k=0}^{q/2} b_k \cos(k\theta_\lambda) = \sum_{k=0}^{q/2} b_k T_k(\lambda). \tag{S49}$$

The only real polynomial of interest, out of the four composing the QSP operator $\mathcal{R}_1\left(\frac{\theta_\lambda}{2},\mathbf{\Phi}_1\right)$, is $\mathrm{Re}\left[B\left(\cos\frac{\theta_\lambda}{2}\right)\right]$, the other three can be determined as to keep achievability. Provided that $\mathrm{Re}\left[B\left(\cos\frac{\theta_\lambda}{2}\right)\right]$ has the form in Eq. (S49), the only remaining condition is that $\left|\mathrm{Re}\left[B\left(\cos\frac{\theta_\lambda}{2}\right)\right]\right| \leq 1$. With the choice $\mathscr{D}(\lambda)=0$ and $\mathscr{B}(\lambda)=\tilde{f}_q(\lambda)$, condition (9) is satisfied, assuring the existence of such $\mathbf{\Phi_1}$.

□

We are now able to prove Lemma 9, which is a direct consequence of Lemmas 6 and 5, and of the qubitization construction.

Proof of Lemma 9. If the QSP sequence $\mathbf{\Phi}_1$ is taken as to reproduce the coefficients of the finite Chebyshev expansion of a continuous target function f (see App. XIII)

$$\tilde{f}_q(\lambda)=\sum_{k=0}^{q/2} b_k T_k(\lambda), \tag{S50}$$

with $f(\lambda)=\tilde{f}_\infty(\lambda)$, then Eq. (S44) means that $V_{\mathbf{\Phi}_1}$ is a perfect block-encoding of $\tilde{f}_q(H)$ a (1,ε')-block-encoding of $f(H)$, with ε' given by the truncation error $\max_{\lambda\in[-1,1]}|f(\lambda)-\tilde{f}_q(\lambda)|$. $\tilde{f}_q(\lambda)$ can be the truncated Chebyshev expansion of $f(\lambda)$ or an interporlating series. f is assumed to be an analytical function and, therefore, ε' is related to the series order $q/2$ according to Lemma 5. The achievability condition (9) is not a strong limitation as f is bounded and we can always redefine $f'(\lambda)=\frac{f(\lambda)}{f_{max}}$, with $f_{max}=\max_{-1\leq\lambda\leq 1} f(\lambda)$. Note that, even though $|\mathscr{B}(\lambda)|\leq 1+\varepsilon'$, it can be rescaled as to satisfy Eq. (9) [7].

Each one of the q operators V_{ϕ_k} or $\tilde{V}_{\phi_k}$ in $V_{\mathbf{\Phi}_1}$ calls the qubitized oracle once which means calling the oracle O_1 twice. Thus $2q$ queries are necessary in total. Moreover the number of gates per query is basicaly given by the number of gates used for qubitization times a constant factor due to the controlled action of O_1'. The control ancilla contributes with a constant small number of gates, such that the total number of gates per query is $\mathcal{O}(g_{O_1}+|\mathcal{A}_{O_1}|)$. □

XVII. PROOF OF LEMMA 12

Our proof is by explicitly construction of $H_{\boldsymbol{x}}$. We use the same 2-sparse Hamiltonians used in the proof of the no-fast-forwarding theorem [3, 15, 16]. Recall that a Hamiltonian matrix is d-sparse if its columns (or rows) contain at most d non-null entries each. As a warm-up, let us first introduce necessary notation for the proof.

Consider the $(N+1)$-dimensional symmetric subspace $\mathbb{H}_{\mathcal{S}_\mathrm{s}}\subset\mathbb{H}_{\mathcal{S}}$ spanned by kets $\{|j\rangle_\mathrm{s}\}_{j\in[N+1]}$, where $|j\rangle_\mathrm{s}$ is the permutationally-invariant superposition of j qubits in state $|1\rangle$ and $N-j$ ones in $|0\rangle$. Let us define the ($\boldsymbol{x}$-independent) Hamiltonian

$$H_0 := \sum_{i=1}^{N}\frac{X_i}{4\,N}\ , \tag{S51}$$

where X_i is the first Pauli operator of the i-th qubit in $\mathcal{S}$. Notice that $\|H_0\|\leq 1$. Since H_0 is proportional to the total angular momentum operator along the x direction, it couples each $|j\rangle_\mathrm{s}$ to $|j-1\rangle_\mathrm{s}$ and $|j+1\rangle_\mathrm{s}$ (or to one of them, depending on the value of j); and, importantly, it leaves $\mathbb{H}_{\mathcal{S}_\mathrm{s}}$ invariant. Besides, the following overlap will be useful too:

$$\left|\langle N|_\mathrm{s}\,F_\beta(H_0)|0\rangle_\mathrm{s}\right| = \left|\frac{1-e^{-\frac{\beta}{2N}}}{2}\right|^N\ , \tag{S52}$$

where the equality follows from the identity $e^{-\beta H_0}=\prod_{i=1}^N e^{-\frac{\beta X_i}{4N}}=\prod_{i=1}^N\left(\cosh(\frac{\beta}{4N})\mathbb{1}-\sinh(\frac{\beta}{4N})X_i\right)$ and the fact that $|N\rangle_\mathrm{s}$ and $|0\rangle_\mathrm{s}$ are both product states.

Proof of Lemma 12. We begin by defining the Hamiltonian $H_{\boldsymbol{x}}$ that encodes $\mathrm{par}(\boldsymbol{x})$. It acts on the $(2N+2)$-dimensional Hilbert space $\mathbb{H}_{\mathcal{S}_\mathrm{s}}\otimes\mathbb{H}_{\mathcal{W}_\mathrm{p}}$, where $\mathcal{W}_\mathrm{p}$ is a single-qubit ancillary write register onto which our QITE-based parity-finding algorithm will write $\mathrm{par}(\boldsymbol{x})$. $H_{\boldsymbol{x}}$ is given by

$$H_{\boldsymbol{x}} := \sum_{j=0}^{N}\frac{\sqrt{(N-j)(j+1)}}{4\,N}\times\left(|j{+}1\rangle\langle j|_\mathrm{s}{+}\mathrm{H.c.}\right)\otimes {X_\mathrm{p}}^{x_j}\ , \tag{S53}$$

where X_p is the first Pauli matrix on $H_{\mathcal{A}_\mathrm{p}}$ and the short-hand notation $|N+1\rangle := 0$ is introduced. Note that $H_{\boldsymbol{x}}$ couples each $|j\rangle_\mathrm{s}$ to $|j-1\rangle_\mathrm{s}$ and $|j+1\rangle_\mathrm{s}$, as H_0 in Eq. (S51), and flips the state of the write register conditioned on the value of the j-th bit x_j of $\boldsymbol{x}$ (see Fig. S9). As a result, $\mathbb{H}_{\mathcal{S}_\mathrm{s}}\otimes\mathbb{H}_{\mathcal{W}_\mathrm{p}}$ is divided into two halves not coupled to one another. Importantly, $|0\rangle_\mathrm{s}|0\rangle_\mathrm{p}$ is always coupled to $|N\rangle_\mathrm{s}|\mathrm{par}(\boldsymbol{x})\rangle_\mathrm{p}$ and never to the wrong-parity state $|N\rangle_\mathrm{s}|\mathrm{par}(\boldsymbol{x})\oplus 1\rangle_\mathrm{p}$. In addition, $H_{\boldsymbol{x}}$ is 2-sparse and $\|H_{\boldsymbol{x}}\|\leq 1$ for all $\boldsymbol{x}\in\{0,1\}^N$.

The following algorithm estimates the parity with a single application of a QITE-primitive on the fixed computational state $|0\rangle_\mathrm{s}|0\rangle_\mathrm{p}|0\rangle_{\mathcal{A}}$ and computational-basis measurements.

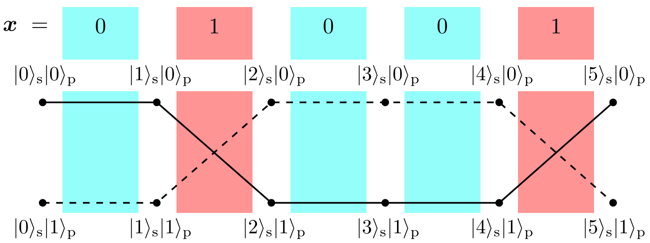

Figure S9. Graph of the couplings present in Hamiltonian $H_{\boldsymbol{x}}$ for $\boldsymbol{x} = 01001$. It encodes the string $\boldsymbol{x}$ in that a flipped-qubit coupling occurs conditioned on x_j, e.g. $|j\rangle_{\rm s}|1\rangle_{\rm p}$ is coupled to $|j+1\rangle_{\rm s}|1\rangle_{\rm p}$ or $|j+1\rangle_{\rm s}|0\rangle_{\rm p}$ if $x_j = 0$ or $x_j = 1$, respectively. Notice that initial state $|0\rangle_{\rm s}|0\rangle_{\rm p}$ is indirectly coupled to $|N\rangle_{\rm s}|{\rm par}(\boldsymbol{x})\rangle_{\rm p} = |5\rangle_{\rm s}|0\rangle_{\rm p}$ (solid lines), not to $|N\rangle_{\rm s}|{\rm par}(\boldsymbol{x})\oplus 1\rangle_{\rm p} = |5\rangle_{\rm s}|1\rangle_{\rm p}$ (dashed lines).

Algorithm: Bit-string parity from QITE

input : $\beta \neq 0$, $\varepsilon' \geq 0$, $\alpha \in (0, 1]$, an $(\beta, \varepsilon', \alpha)$-QITE-primitive P querying a block-encoding oracle for $H_{\boldsymbol{x}}$, an input state $|0\rangle_{\rm s}|0\rangle_{\rm p}|0\rangle_{\mathcal{A}}$

output: ${\rm par}(\boldsymbol{x})$ with probability $> 1/2$

apply P on $|0\rangle_{\rm s}|0\rangle_{\rm p}|0\rangle_{\mathcal{A}}$;
measure $\mathcal{A}$ in the computational basis;
if *outcome is* $|0\rangle_{\mathcal{A}}$ **then** proceed;
else output ${\rm par}(\boldsymbol{x}) = 0$ or 1 with probability $1/2$; end algorithm;
measure $\mathcal{S}_{\rm s}$ on basis $\{|j\rangle_{\rm s}\}_j$;
if *outcome is* $|N\rangle_{\rm s}$ **then** proceed;
else output ${\rm par}(\boldsymbol{x}) = 0$ or 1 with probability $1/2$; end algorithm;
measure $\mathcal{W}_{\rm p}$ in the computational basis and output the outcome;

The measurement on $\mathcal{A}$ is the usual QITE post-selection heralding whether $F_\beta(H_{\boldsymbol{x}})$ has been applied on $|0\rangle_{\rm s}|0\rangle_{\rm p}$. The measurement on $\mathcal{S}_{\rm s}$ heralds whether the fully-excited state $|N\rangle_{\rm s}$ has been obtained and, hence, whether the desired state $|{\rm par}(\boldsymbol{x})\rangle_{\rm p}$ has been prepared in the write register $\mathcal{W}_{\rm p}$. Let us analyze the overall success probability.

Consider first the restricted, simplified case of $\varepsilon' = 0$. Applying a $(\beta, 0, \alpha)$-QITE-primitive on $|0\rangle_{\rm s}|0\rangle_{\rm p}|0\rangle_{\mathcal{A}}$ and then measuring $|0\rangle_{\mathcal{A}}$ on $\mathcal{A}$ prepares a state whose overlap with $|N\rangle_{\rm s}|{\rm par}(\boldsymbol{x})\rangle_{\rm p}|0\rangle_{\mathcal{A}}$ is given by Eq. (S52), as a straightforward calculation shows, which is strictly greater than 0. In turn, its overlap with $|N\rangle_{\rm s}|{\rm par}(\boldsymbol{x})\oplus 1\rangle_{\rm p}|0\rangle_{\mathcal{A}}$ is zero. Hence, conditioned on a correct post-selection on $\mathcal{A}$ and on measuring $|N\rangle_{\rm s}$ too, the success probability of obtaining the correct parity on $\mathcal{W}_{\rm p}$ is unit. If any other measurement outcome is obtained, the algorithm simply makes a coin toss that guesses the correct parity with probability $1/2$. Therefore, for any non-null post-selection probability, the overall success probability of getting ${\rm par}(\boldsymbol{x})$ is strictly greater than $1/2$.

For $\varepsilon' > 0$, not any non-null overlap with $|N\rangle_{\rm s}|{\rm par}(\boldsymbol{x})\rangle_{\rm p}|0\rangle_{\mathcal{A}_P}$ suffices any longer, since the overlap with $|N\rangle_{\rm s}|{\rm par}(\boldsymbol{x})\oplus 1\rangle_{\rm p}|0\rangle_{\mathcal{A}_P}$ is now also nonzero. More precisely, we need to demand that, conditioned on measuring $|0\rangle_{\mathcal{A}_P}$ and $|N\rangle_{\rm s}$, the correct-parity output has greater probability than the incorrect one:

$$\left|\langle N|_{\rm s}\langle {\rm par}(\boldsymbol{x})|_{\rm p}\langle 0|_{\mathcal{A}_P}|\tilde{\Psi}_\beta(\boldsymbol{x})\rangle\right| > \left|\langle N|_{\rm s}\langle {\rm par}(\boldsymbol{x})\oplus 1|_{\rm p}\langle 0|_{\mathcal{A}_P}|\tilde{\Psi}_\beta(\boldsymbol{x})\rangle\right| \ , \quad \text{(S54)}$$

where $|\tilde{\Psi}_\beta(\boldsymbol{x})\rangle$ is the state after the QITE primitive. Since the primitive generates an (α, ε')-block-encoding of the QITE propagator $F_\beta(H_{\boldsymbol{x}})$, we following bounds must hold

$$\left|\langle N|_{\rm s}\langle {\rm par}(\boldsymbol{x})|_{\rm p}\langle 0|_{\mathcal{A}_P}|\tilde{\Psi}_\beta(\boldsymbol{x})\rangle\right| \geq \alpha|\langle N|_{\rm s}\langle {\rm par}(\boldsymbol{x})|_{\rm p}\, F_\beta(H_{\boldsymbol{x}})|0\rangle_{\rm s}|0\rangle_{\rm p}| - \varepsilon' \ , \quad \text{(S55a)}$$

and

$$\left|\langle N|_{\rm s}\langle {\rm par}(\boldsymbol{x})\oplus 1|_{\rm p}\langle 0|_{\mathcal{A}_P}\,|\tilde{\Psi}_\beta(H_{\boldsymbol{x}})\rangle\right| \leq \varepsilon' \ . \quad \text{(S55b)}$$

From these, we see that Eq. (S54) is fulfilled if we impose

$$\alpha|\langle N|_{\rm s}\langle {\rm par}(\boldsymbol{x})|_{\rm p}\, F_\beta(H_{\boldsymbol{x}})|0\rangle_{\rm s}|0\rangle_{\rm p}| - \varepsilon' > \varepsilon' \ . \quad \text{(S56)}$$

Using again the fact that $|\langle N|_{\rm s}\langle {\rm par}(\boldsymbol{x})|_{\rm p}\, F_\beta(H_{\boldsymbol{x}})|0\rangle_{\rm s}|0\rangle_{\rm p}|$ equals the overlap in Eq. (S52), one can straightforwardly see that Eq. (S56) is equivalent to Eq. (26). □

Finally, we notice that the factor 4 in the denominator of Eq. (S53) is not necessary for the above proof argument. However, it guarantees that $\|H_{\boldsymbol{x}}\| \leq 1$, which is necessary for $H_{\boldsymbol{x}}$ to be block-encodable, as required for Lemma 13.

XVIII. PROOF OF LEMMA 13

The proof is constructive. We design a quantum circuit (see Fig. S10) that generates a block-encoding $U_{H_{\boldsymbol{x}}}$ of $H_{\boldsymbol{x}}$ from a single application of $U_{\boldsymbol{x}}$.

Proof of Lemma 13. We first need to define the total angular-momentum ladder operators $J_\pm$. In our notation, $J_+|j\rangle_s := \sqrt{(N-j)(j+1)}|j+1\rangle_s$ and $J_-|j\rangle_s := \sqrt{(N-j+1)j}|j-1\rangle_s$. Now, using Eqs. (25) and (S53), note that

$$H_{\boldsymbol{x}} = \frac{J_+ U_{\boldsymbol{x}} + U_{\boldsymbol{x}} J_-}{4\,N}\,. \tag{S57}$$

The ladder operators can also be written in terms of Pauli operators as $J_\pm = \sum_{i=0}^{N-1} X_i \pm iY_i$. Since they are a linear combination of $2\,N$ unitaries, standard methods [6] can be used to produce a block encoding $U_{J_\pm}$ of $J_\pm/(2N)$ using an ancillary register $\mathcal{A}_{O_J}$ with $|\mathcal{A}_{O_J}| = \mathcal{O}\big(\log(N)\big)$ qubits. This requires no query to $U_{\boldsymbol{x}}$.

Then, the circuit in Fig. S10 shows how to generate a perfect block-encoding $U_{H_{\boldsymbol{x}}}$ of $H_{\boldsymbol{x}}$ from one query to U_{J_+}, one query to U_{J_-}, and one query to $U_{\boldsymbol{x}}$, and using a single-qubit ancila $\mathcal{A}_{O'}$. Finally, a block-encoding oracle controlled-$U_{H_{\boldsymbol{x}}}$ for $H_{\boldsymbol{x}}$ can be obtained by replacing every gate in the figure with its version controlled by an extra single-qubit ancilla $\mathcal{A}_{O''}$ (not represented in Fig. S10). In the notation of Def. 2, it reads $\mathcal{A}_{O_1} = \{\mathcal{A}_{O_J}, \mathcal{A}_{O'}, \mathcal{A}_{O''}\}$. This extra control does not alter the number of queries to $U_{\boldsymbol{x}}$. □

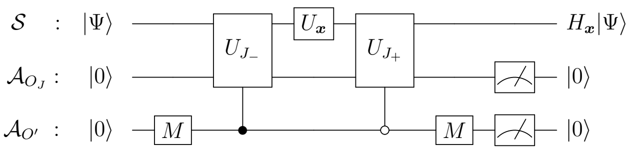

Figure S10. Generation of a block-encoding $U_{H_{\boldsymbol{x}}}$ of $H_{\boldsymbol{x}}$ from a single query to $U_{\boldsymbol{x}}$. $\mathcal{A}_{O_J}$ is the ancillary register required to block-encode $J_\pm$ and $\mathcal{A}_{O'}$ is the single-qubit ancilla required to create the linear combination in Eq. (S57).

[1] E. Campbell, Random compiler for fast hamiltonian simulation, Phys. Rev. Lett. **123**, 070503 (2019).
[2] A. M. Childs, A. Ostrander, and Y. Su, Faster quantum simulation by randomization, Quantum **3** (2019).
[3] D. W. Berry, A. M. Childs, and R. Kothari, Hamiltonian simulation with nearly optimal dependence on all parameters, 2015 IEEE 56th Annual Symposium on Foundations of Computer Science , 792 (2015), arXiv:1501.01715.
[4] D. W. Berry, A. M. Childs, R. Cleve, R. Kothari, and R. D. Somma, Simulating Hamiltonian Dynamics with a Truncated Taylor Series, Physical Review Letters **114**, 090502 (2015), arXiv:arXiv:1412.4687.
[5] G. H. Low and I. L. Chuang, Optimal hamiltonian simulation by quantum signal processing, Phys. Rev. Lett. **118**, 010501 (2017).
[6] G. H. Low and I. L. Chuang, Hamiltonian Simulation by Qubitization, Quantum **3**, 163 (2019).
[7] A. Gilyén, Y. Su, G. H. Low, and N. Wiebe, Quantum singular value transformation and beyond: Exponential improvements for quantum matrix arithmetics, in *Proceedings of the 51st Annual ACM SIGACT Symposium on Theory of Computing*, STOC 2019 (Association for Computing Machinery, New York, NY, USA, 2019) p. 193.
[8] J. van Apeldoorn, A. Gilyén, S. Gribling, and R. de Wolf, Quantum SDP-Solvers: Better upper and lower bounds, Quantum **4**, 230 (2020).
[9] G. H. Low, T. J. Yoder, and I. L. Chuang, Methodology of resonant equiangular composite quantum gates, Phys. Rev. X **6**, 041067 (2016).
[10] J. Haah, Product Decomposition of Periodic Functions in Quantum Signal Processing, Quantum **3**, 190 (2019).
[11] R. Chao, D. Ding, A. Gilyen, C. Huang, and M. Szegedy, Finding angles for quantum signal processing with machine precision (2020), arXiv:2003.02831 [quant-ph].
[12] Y. Dong, X. Meng, K. B. Whaley, and L. Lin, Efficient phase factor evaluation in quantum signal processing (2020), arXiv:2002.11649 [quant-ph].
[13] W. Fraser, A survey of methods of computing minimax and near-minimax polynomial approximations for functions of a single independent variable, Journal of the Association for Computing machinery **12**, 295 (1965).
[14] D. Elliott, D. Paget, G. Phillips, and P. Taylor, Error of truncated Chebyshev series and other near minimax polynomial approximations, Journal of Approximation Theory **50**, 49 (1987).
[15] D. W. Berry, G. Ahokas, R. Cleve, and B. C. Sanders, Efficient Quantum Algorithms for Simulating Sparse Hamiltonians, Communications in Mathematical Physics **270**, 359 (2007).
[16] D. W. Berry, A. M. Childs, R. Cleve, R. Kothari, and R. D. Somma, Exponential improvement in precision for simulating sparse Hamiltonians, in *Proceedings of the forty-sixth annual ACM symposium on Theory of computing* (ACM, New York, NY, USA, 2014) pp. 283–292, arXiv:1312.1414.
